# Synthesis and Fungicidal Evaluation of Pyridylpyrazole Derivatives Bearing a Benzoxazinone Scaffold via a Metal-Free Approach

**DOI:** 10.3390/ijms27135903

**Published:** 2026-06-30

**Authors:** Bin-Ran Zeng, Jing-Ya Liu, Rui-Han Hu, Xiao-Hua Du

**Affiliations:** Catalytic Hydrogenation Research Center, Zhejiang Key Laboratory of Green Pesticides and Cleaner Production Technology, Zhejiang Green Pesticide Collaborative Innovation Center, Zhejiang University of Technology, Hangzhou 310014, China; 221122010358@zjut.edu.cn (B.-R.Z.); 211123010228@zjut.edu.cn (J.-Y.L.); 211122010185@zjut.edu.cn (R.-H.H.)

**Keywords:** pyridylpyrazole derivatives, metal-free synthesis, structure–activity relationship, fungicidal activity

## Abstract

To develop novel, green, and highly effective fungicides, a series of benzoxazinone-functionalized pyridylpyrazole derivatives (**8a**–**8z**) were designed and synthesized via a metal-catalyst-free route using a pyraziflumid intermediate. The synthetic procedure included diazotization, cyclization, hydrolysis, and methanesulfonyl chloride-mediated dehydrative cyclization. The optimal conditions (3.0 equiv pyridine in water, BF_4_^−^ as the counterion, 25 °C, 3 h) afforded a maximum yield of 63.7%. Bioassays showed that **8p** exhibited 92.0% inhibition against maize rust (*Puccinia polysora*) and **8q** exhibited 90.5% inhibition against cucumber anthracnose (*Colletotrichum orbiculare*) at 50 mg/L. Substituents on the aromatic and pyridyl moieties jointly regulated fungicidal activity, with distinct pathogen-dependent selectivity. Docking results revealed that **8j** bound to succinate dehydrogenase (SDHI) with a binding energy of −11.8 kcal/mol, and the polar interaction between its carbonyl group and Lys163 was key to enhancing binding affinity. This study provides a scientific basis for the green synthesis and rational design of pyridylpyrazole-based fungicides.

## 1. Introduction

Nitrogen-containing heterocycles constitute a fundamental class of privileged scaffolds in modern agrochemical discovery, owing to their remarkable ability to interact with diverse biological targets through hydrogen bonding, π–π stacking, and hydrophobic interactions. Among these, pyridine and pyrazole rings have emerged as particularly valuable pharmacophores, as they are frequently encountered in commercially successful fungicides, herbicides, and insecticides [1,2]. The strategic combination of these two heterocycles into a single molecular framework—giving rise to pyridylpyrazole derivatives—has proven to be a productive approach for generating novel crop protection agents with enhanced potency and selectivity [3,4,5,6,7].

SDHI fungicides represent a compelling illustration of this strategy. Starting from the lead structure of boscalid, systematic modifications of both the acid and amine moieties have led to the development of highly effective commercial varieties such as fluxapyroxad, bixafen, and penthiopyrad [8,9,10,11]. These successes underscore the exceptional structural tunability and design potential inherent in pyridylpyrazole-based skeletons [12,13,14]. Nevertheless, two major fungal diseases—maize rust and cucumber anthracnose—continue to pose serious threats to global crop production. Maize rust can cause substantial yield losses under favorable epidemic conditions, while cucumber anthracnose spreads rapidly in warm, humid environments, infecting leaves, stems, and fruits and severely constraining the safe production of this important vegetable crop [15,16,17,18]. The development of highly effective, low-toxicity fungicides targeting these pathogens therefore remains an urgent priority [19].

From a synthetic perspective, conventional methods for constructing the pivotal pyridine–pyrazole C–C bond have predominantly relied on transition-metal-catalyzed cross-coupling reactions, particularly Suzuki–Miyaura coupling [20,21,22,23,24]. While these protocols are highly reliable and exhibit broad substrate scope, they carry inherent drawbacks that are particularly problematic in the context of green and sustainable pesticide development. These include high catalyst costs, elevated reaction temperatures, and—most critically—the risk of heavy metal residues in the final products. Such residues not only complicate purification but also raise significant environmental and regulatory barriers for agricultural applications [25,26]. These limitations underscore the urgent need for a metal-free alternative. In response, the present study develops a strategy that avoids these issues entirely by eliminating the reliance on transition metals, thereby offering a more sustainable and intrinsically safer approach for constructing this pharmaceutically relevant scaffold.

We report herein an efficient, metal-free strategy for the construction of pyridylpyrazole derivatives. The key C–C bond is forged under mild conditions via direct cyclization of substituted anilines—after diazotization—with substituted pyridines, without the need for any metal catalyst. Notably, nitrogen gas is the sole byproduct, rendering the process intrinsically cleaner than traditional cross-coupling approaches [27]. An additional advantage of this methodology is its ability to simultaneously access isomers with different pyridine substitution patterns, offering a level of synthetic flexibility that is difficult to achieve with metal-catalyzed protocols. Following this key C–C bond formation, the resulting intermediates are subjected to hydrolysis and subsequent dehydrative cyclization with various substituted anthranilic acids in the presence of methanesulfonyl chloride [28,29]. By systematically varying the type, position, and number of substituents on the benzene ring, we have rationally constructed a library of pyridylpyrazole derivatives bearing a benzoxazinone moiety (**8a**–**8z**). The bioassay results reveal that most synthesized compounds exert prominent activities against maize rust and cucumber anthracnose, with distinct structure–activity relationships established for both pathogens. This work not only confirms the practicability of sustainable metal-free synthetic pathways but also offers valuable structural references for the rational design of next-generation agricultural fungicides featuring high potency and favorable environmental compatibility.

## 2. Results and Discussion

### 2.1. Synthetic Optimization of Novel Pyridylpyrazole Derivatives

To improve the synthetic efficiency and reduce the cost of pyridyl-pyrazole compounds, a metal-free strategy distinct from conventional Suzuki coupling was developed. The target compounds were accessed by reacting substituted pyrazoles, after diazotization to their tetrafluoroborate salts, with substituted pyridines. This approach exhibits broad substrate scope and allows for diverse structural variations, thereby markedly enhancing the efficiency of constructing this compound class.

**Scheme 1:**
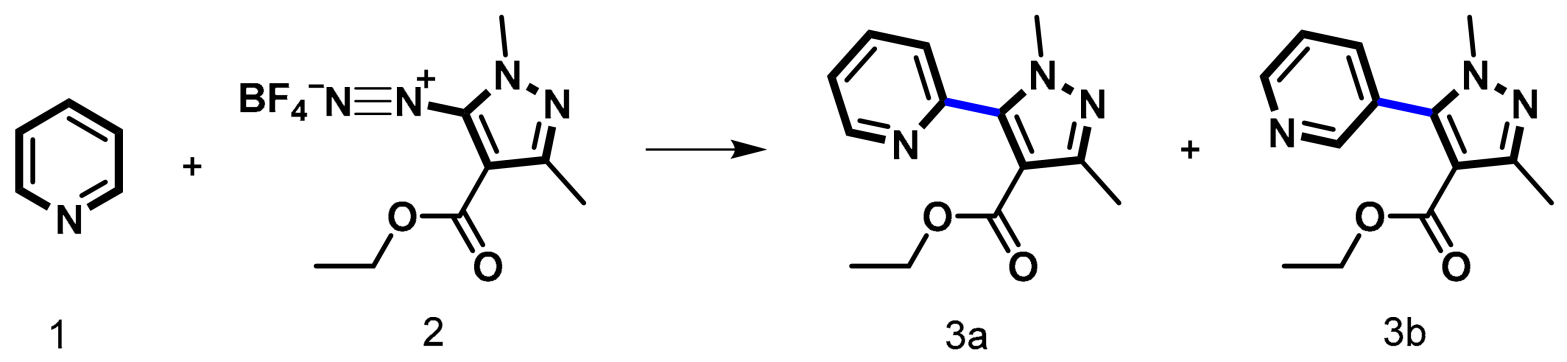
Synthetic route for the model pyridyl-pyrazole compounds (The blue lines represent the newly formed bonds).

With compound **1** (the active intermediate of pyraziflumid) and pyridine as model substrates (Scheme 1), the effects of solvent and diazonium salt counterion were first investigated, and the results are summarized in Table 1.

As shown in Table 1, deionized water afforded the best result, giving the ortho-isomer in 56.4% yield. The yields obtained in acetonitrile, DMSO, and DMF were 49.6%, 53.5%, and 49.7%, respectively. In contrast, significantly lower yields were observed in ethanol, dichloromethane, ethyl acetate, and toluene; a side reaction similar to the above reaction occurred when toluene was used as a solvent, resulting in the formation of phenylpyrazole byproduct. With deionized water selected as the solvent, the effect of different diazonium salt counteranions (BF_4_^−^, H_2_PO_4_^−^, Cl^−^, and HSO_4_^−^) on the reaction yield was subsequently compared. The BF_4_^−^ anion provided the highest yield (56.4%), followed by HSO_4_^−^ (37.4%), whereas H_2_PO_4_^−^ and Cl^−^ gave only 11.8% and 22.5% yields, respectively. To further optimize the reaction conditions, three factors—pyridine amount, reaction time, and reaction temperature—were investigated using an orthogonal experimental design, and the results are presented in Table 2.

**Figure 1:**
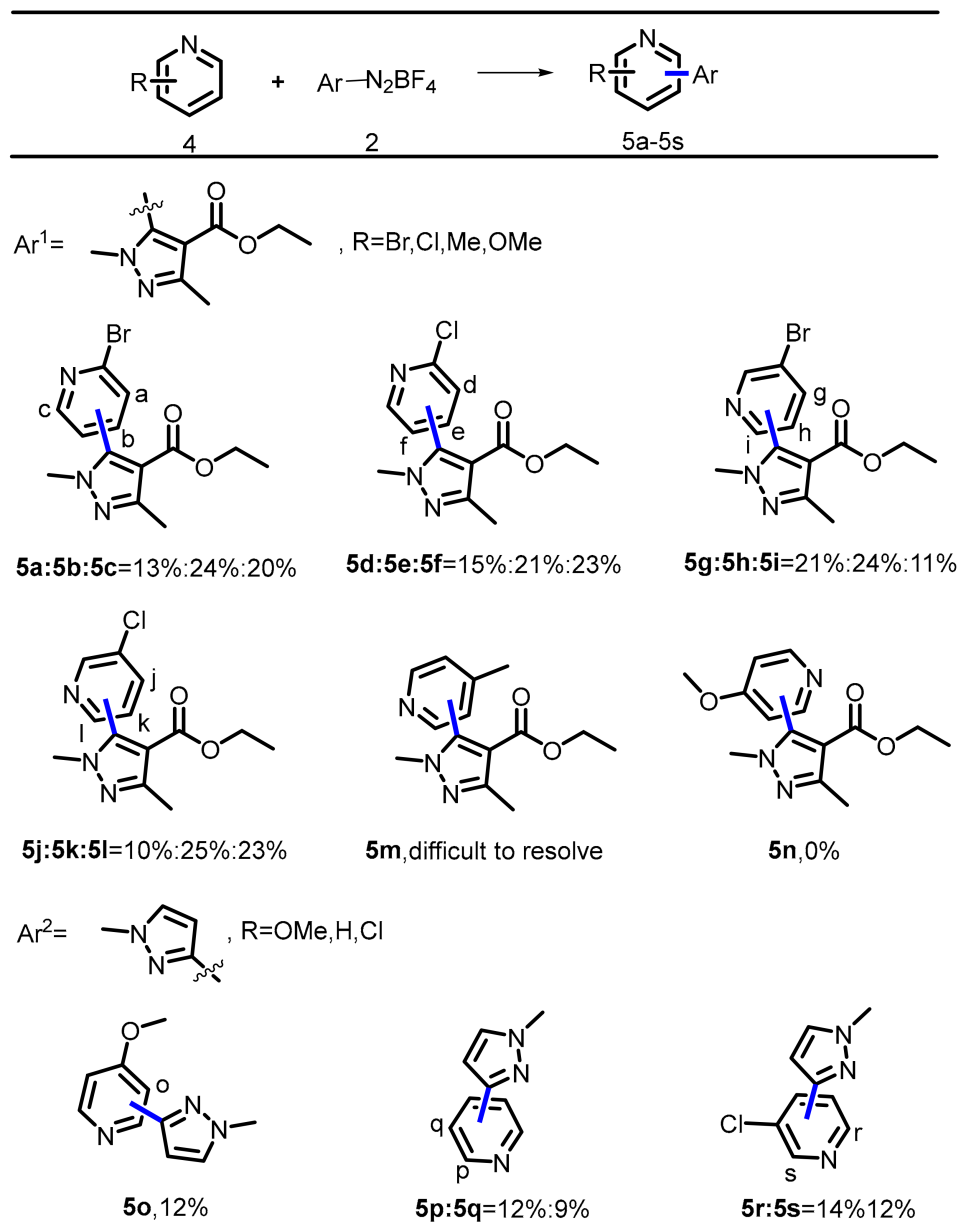
Synthesis of compounds **5a**–**5s** (The blue lines represent the newly formed bonds).

Range analysis based on Table 2 indicated that the influencing order of each factor on the reaction yield was pyridine dosage > reaction temperature > reaction time. Pyridine dosage acted as the predominant factor, followed by reaction temperature. Accordingly, the optimal reaction conditions were determined as 3.0 equivalents of pyridine at 25 °C for 3 h. To verify the general applicability of this synthetic protocol, substrate scope exploration was performed under the optimized conditions, and a series of pyridylpyrazole derivatives (**5a**–**5s**) were successfully synthesized. The corresponding reaction route and product structures are illustrated in Figure 1.

Following the synthetic route outlined in Scheme 1, we first reacted pyridines bearing substituents at different positions and successfully obtained a series of novel pyridylpyrazole compounds (the Ar^1^ series, Figure 1). To evaluate the generality of this methodology, various substituted pyridines were further treated with 3-amino-1-methylpyrazole, affording the corresponding target products (the Ar^2^ series, Figure 1). Notably, the reaction proceeds under mild, metal-free conditions with nitrogen as the sole byproduct, highlighting a clear advantage in green synthesis over classical Suzuki coupling. In addition, this protocol allows multiple target compounds to be prepared and isolated in a single run, which significantly improves synthetic efficiency and is particularly beneficial for accelerating the discovery of novel agrochemicals.

This work systematically investigates the regioselectivity of the metal-free C–H arylation between substituted pyridines and pyrazole diazonium salts, elucidating the interplay of substituent position, steric hindrance, and electronic effects. The results demonstrate that steric hindrance of the pyridine substituent is the dominant factor governing regioselectivity, whereas electronic effects primarily modulate substrate reactivity and exert only a secondary influence on product distribution. For halogen-substituted pyridines, regioselectivity depends strongly on both the position and the size of the halogen. 2-Bromopyridine undergoes preferential arylation at C4 due to the pronounced steric bulk of bromine, while 2-chloropyridine tends to react at C5 owing to the smaller atomic radius of chlorine, illustrating the steric tuning of selectivity. Both 3-bromopyridine and 3-chloropyridine give exclusively C5-arylated products, which is attributed to steric shielding of C3 and C4 by the halogen at C3. Substrates with electronically distinct substituents further reveal the synergy between steric and electronic effects. 4-Methylpyridine affords the arylated product **5m**. The obtained regioisomers possess similar polarity and thus cannot be readily separated, which indicates that the introduction of a methyl group barely alters the overall polarity of pyridinyl-pyrazole isomers. Notably, 4-methoxypyridine shows no reactivity (**5n**, 0%). This can be rationalized by the strong electron-donating ability of the methoxy group, which markedly increases the electron density on the pyridine ring and suppresses the electrophilic arylation process. These trends are further corroborated in reactions with the less sterically demanding 1-methylpyrazole diazonium salt (Ar^2^). 3-Methoxypyridine gives the C4-arylated product **5o** in 12% yield. Unsubstituted pyridine and 3-chloropyridine both exhibit low regioselectivity, with only a slight preference for specific sites.

In summary, this study establishes a regioselectivity model dominated by steric hindrance and synergistically modulated by electronic effects. These findings provide valuable guidance for the rational design of pyridine C–H functionalization and lay a theoretical foundation for the targeted synthesis and structure–activity relationship studies of pyridinyl-pyrazole derivatives, which represent promising lead scaffolds for novel pesticides.

### 2.2. Synthesis of Target Antifungal Compounds

**Figure 2:**
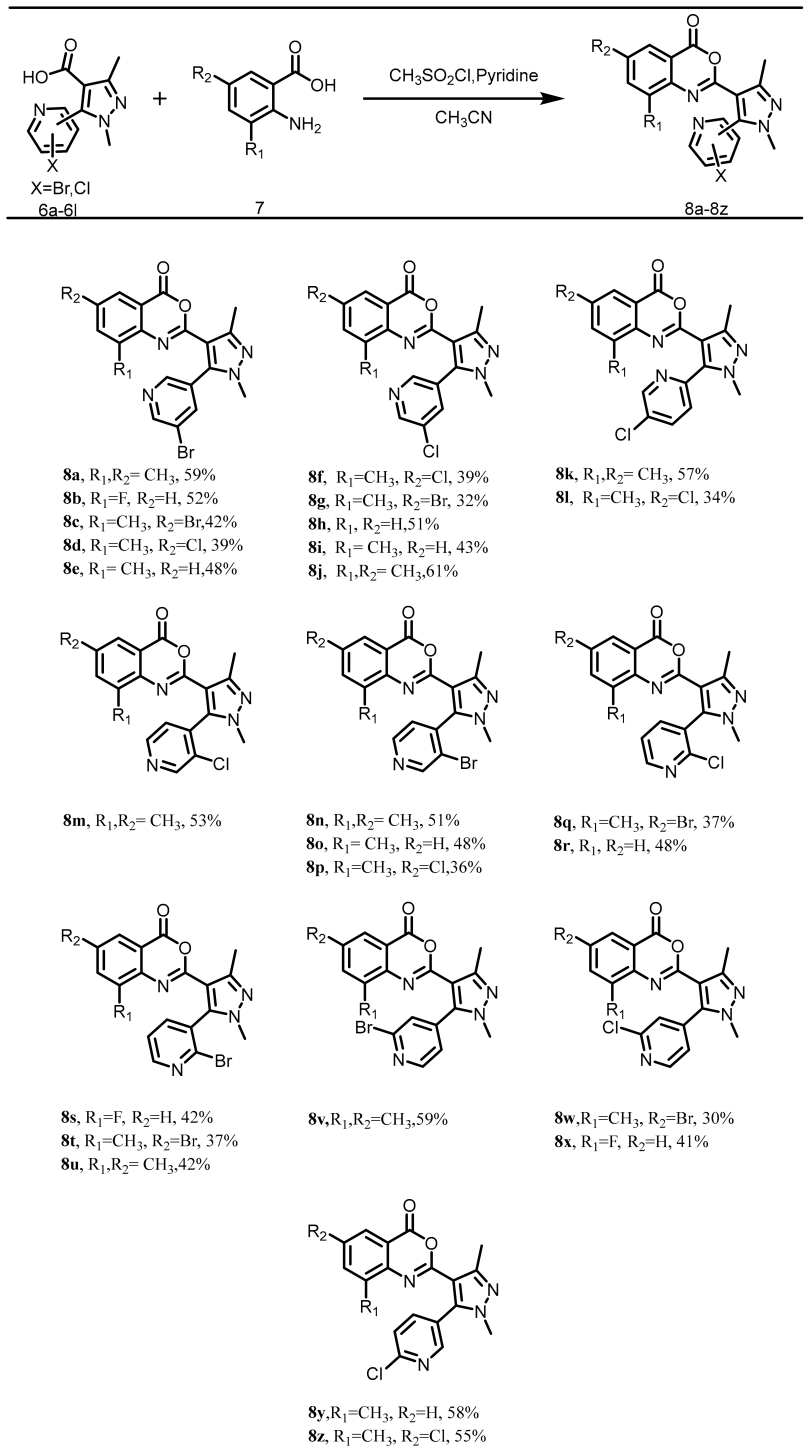
Synthesis of compounds **8a**–**8z**.

The target compounds were designed with a benzoxazinone skeleton as the core structure. A series of derivatives **8a**–**8z** were synthesized by introducing substituents with different electronic effects and steric hindrance onto the benzene ring of anthranilic acid and the pyridine ring of the pyrazole side chain. The synthetic route is illustrated in Figure 2. Using the novel pyridylpyrazole compounds in Figure 1 as starting materials, intermediates **6a**–**6l** were obtained via alkaline hydrolysis followed by acidification. The crude products were used directly in the next step without further purification. Subsequently, intermediates **6a**–**6l** reacted with various substituted anthranilic acids in the presence of methanesulfonyl chloride to afford the target compounds **8a**–**8z**.

### 2.3. Determination of In Vitro and In Vivo Fungicidal Activities

To gain more accurate fungicidal activity data, we carried out both in vitro and in vivo bioassays in this study. The fungicidal performance of the tested compounds against maize rust and cucumber anthracnose is listed in Table 3. Preliminary bioactivity evaluation demonstrated that most compounds possessed good inhibitory effects on the two plant diseases.

Based on the bioactivity results against maize rust, substituents on the anthranilic acid-derived benzene core markedly affected the fungicidal activity of target compounds. The electronic effect and steric hindrance of aromatic substituents collectively governed the binding capacity between test molecules and the pathogen active site. Compounds with methyl at R_1_ and methyl or chlorine at R_2_ exhibited the best comprehensive activity. The methyl group at R_1_ enhanced hydrophobic interaction inside the binding pocket, and chlorine at R_2_ offered moderate steric hindrance and mild electron-withdrawing effect, which fitted well with the target cavity of maize rust. Compound **8j** with two methyl groups on the benzene ring gave a 100% inhibition rate at 200 mg/L. Compound **8p** (R_1_ = CH_3_, R_2_ = Cl) exhibited inhibition rates of 97.0% and 92.0% at 200 mg/L and 50 mg/L, respectively, demonstrating excellent fungicidal performance across different concentrations. Benzene ring modified with hydrogen or strong electron-withdrawing groups resulted in reduced activity, proving that methyl and chlorine were favorable substituents for anti-maize rust activity. As for the pyridine side chain, para-chloropyridine and ortho-chloropyridine were typical privileged moieties. Such heterocyclic fragments worked synergistically with methyl and chlorine substituents on the anthranilic acid benzene ring to elevate the inhibitory activity.

A totally different SAR trend was observed for cucumber anthracnose, suggesting remarkable pathogen selectivity derived from core ring substitution. Dimethyl substitution on the benzene ring caused poor activity, because the bulky hydrophobic methyl groups brought extra steric hindrance and disturbed molecular binding with the target site. By contrast, neutral hydrogen and halogen atoms were beneficial to fungicidal activity. Among these halogens, fluorine possessed weak electron-withdrawing ability, whereas chlorine and bromine belonged to moderately electron-withdrawing groups. All these substituents had small spatial volume, which kept suitable molecular polarity and promoted the interaction between compounds and targets. Compound **8q** (R_2_ = Br) showed the highest activity with an inhibition rate of 90.5% at 50 mg/L. Compounds **8b**, **8d**, **8h, 8r** and **8w** also maintained inhibition rates above 80% under the same condition, which validated that H, F and Br were ideal substituents on the anthranilic acid benzene ring. Furthermore, para-chloropyridine was identified as a key privileged side chain, which could effectively improve the activity against cucumber anthracnose for most core derivatives.

In summary, the fungicidal activity of these benzoxazinone derivatives is controlled by the electronic effects and steric hindrance of substituents on the core skeleton, along with the heterocyclic structure of the side chain. The two pathogens showed distinct structural preferences, which can be attributed to the structural differences in their target binding pockets. This work clarifies the key active sites and privileged structural fragments and provides a theoretical foundation for the further rational design of highly selective fungicidal lead compounds.

Table 4 summarizes the toxicity regression equations, correlation coefficients (R), and EC_50_ values of the target compounds against corn rust. We carried out bioassays across multiple concentration gradients including low concentrations; the full inhibition rate data for each concentration are available in the Supporting Information (Table S2). The EC_50_ values were 23.49 mg/L (**8a**), 22.38 mg/L (**8j**), 16.83 mg/L (**8l**), and 21.65 mg/L (**8p**), all of which were higher than that of the positive control azoxystrobin (EC_50_ = 1.411 mg/L, as reported in [30]). Although compound **8l** showed the best activity among the tested analogs (EC_50_ = 16.83 mg/L), its potency was still significantly lower than that of the commercial fungicide. The correlation coefficients (R) of all regression equations exceeded 0.95, confirming a strong linear relationship between log concentration and the probit of inhibition rate and validating the reliability of the bioassay results.

### 2.4. Docking Analysis

**Figure 3:**
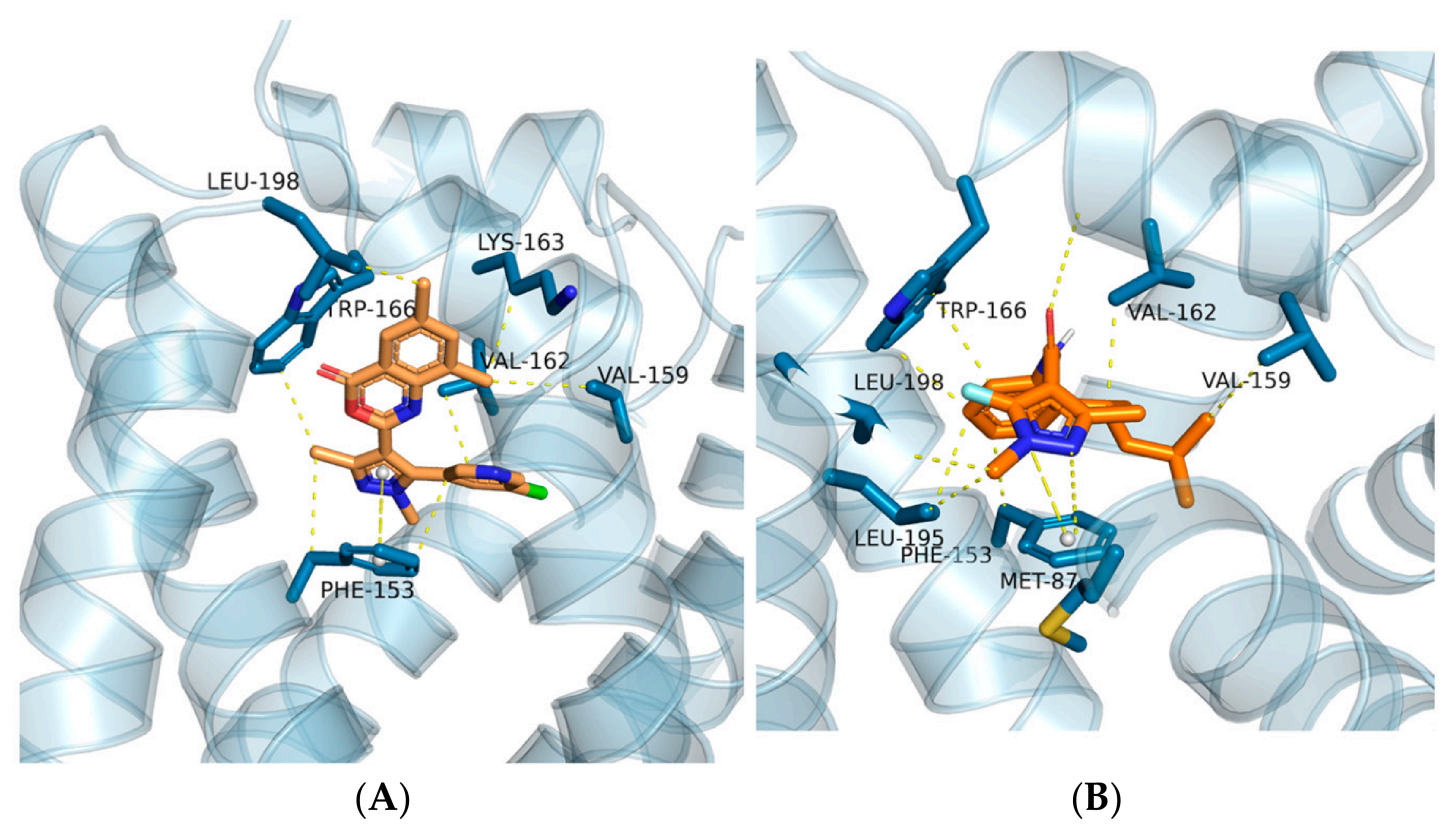
(**A**) Binding mode of compound **8j** with target protein. (**B**) Binding mode of pyraziflumid with target protein. (Dashed lines: hydrogen bonds; green: Cl; blue: N; red: O; light blue: F).

In this study, molecular docking was performed using the Lamarckian genetic algorithm implemented in AutoDock 4.2 software. The crystal structure of the target protein was retrieved from the Protein Data Bank (PDB ID: 9QDL), and the structures of small molecules were drawn using ChemDraw20.0. Both the target protein and ligand structures were processed with AutoDockTools, including the addition of hydrogen atoms and Gasteiger–Hückel charges, merging of non-polar hydrogens, and assignment of rotatable bonds. All σ bonds between heavy atoms in the ligand were set as rotatable, while the template molecule was treated as a rigid structure. During the docking procedure, a grid box of 120 × 120 × 120 grid points with a spacing of 0.375 Å was centered on the binding cavity of the protein. Each ligand was subjected to 100 independent docking runs at the binding site. The Lamarckian genetic algorithm generated 120 random orientations and conformations of the ligand. Each generation underwent a maximum of 2,500,000 energy evaluations, and the top 10 conformations were selected for propagation. The crossover rate was set to 0.8, the mutation rate to 0.02, and the optimization was terminated after 27,000 generations. All other parameters were kept at their default settings in AutoDock 4.2. Upon completion, all docking poses were subjected to cluster analysis, and the binding conformation with the best docking score (the lowest energy value) was selected from the optimal cluster to determine the preferred binding site of the template molecule (pyraziflumid). The binding mode with the target protein is shown in Figure 3.

Furthermore, the optimal binding site was identified from the docking results obtained with AutoDock 4.2, and a docking grid box center was defined based on this site (x center = 129.038, y center = 133.595, z center = 109.148). A grid box of 40 × 40 × 40 grid points with a spacing of 0.375 Å was set at the protein cavity. Based on this grid box, virtual screening of the 26 compounds synthesized in this study was performed using Vina (PyMOL2.6) docking software. Each ligand was subjected to 20 independent docking runs at the binding site, yielding five diverse conformations for analysis [31,32].

To further explore the mode of action of these compounds, molecular virtual screening was performed targeting SDH protein using AutoDock Vina. Combined analysis of binding energy data and in vitro bioassay results revealed that the binding capacity of these compounds to SDH protein was significantly positively correlated with their fungicidal activity. Virtual screening results demonstrated that all target compounds (**8a**–**8z**) could spontaneously bind to the target protein and possessed potential fungicidal activity, which was further verified by subsequent biological assays. Among all derivatives, compound **8j** exhibited the strongest binding affinity and excellent inhibitory activity against the maize rust pathogen. It achieved 100% inhibition at 200 mg/L and maintained an inhibition rate of 62.0% at 50 mg/L, indicating good agreement between the docking binding energy and the actual fungicidal effect. Compound **8p**, with a moderate docking binding energy of −9.7 kcal/mol, displayed the most potent activity at low concentration, reaching an inhibition rate of 92.0% at 50 mg/L. These results indicated that the biological activity of the compounds was not solely determined by molecular binding energy; other factors including cell membrane permeability and physicochemical properties also played important roles. In addition, compounds **8l** and **8v**, with favorable binding energies, exhibited satisfactory inhibitory effects against maize rust, whereas compounds **8h** and **8r**, with the weakest binding affinity, showed relatively poor activity. Overall, the docking scores of this series of compounds were significantly positively correlated with their activity against maize rust.

Molecular docking results showed that compound **8j** possessed the optimal binding performance, with a binding energy of −11.8 kcal/mol, which was markedly superior to that of the reference fungicide fluxapyroxad (−8.9 kcal/mol). Both compounds could bind to the conserved hydrophobic pocket of SDH and interact with amino acid residues Phe153, Trp166, Val159, Val162, and Leu198 via hydrophobic and π–π stacking interactions. The difference in their binding capacity was in good agreement with the calculated docking energy values. The superior binding affinity of compound **8j** was mainly attributed to the stable polar interaction formed between its carbonyl group and Lys163 residue, while the terminal chlorine atom also conferred favorable lipophilicity to the molecule. In contrast, fluxapyroxad had a binding energy of only −8.9 kcal/mol. Although it could be inserted deeper in the binding cavity and form additional hydrophobic interactions with Leu195 and Met87 residues, it failed to establish a stable polar interaction with Lys163. Moreover, the electron-withdrawing effect of the fluorine atom further weakened its interactions with surrounding residues, ultimately resulting in lower binding stability than compound **8j**. Collectively, these findings confirm that the polar interaction with Lys163 is a key factor for improving the binding affinity of this compound series, and the extra hydrophobic interactions of the reference drug cannot compensate for the lack of this critical polar anchoring effect.

Structure–activity relationship analysis indicated that the benzoxazinone scaffold provided a stable foundation for target protein binding, and the electronic and steric effects of the side-chain substituents could remarkably alter the molecular binding mode. The spatial configuration of the substituent in compound **8j** matched well with the binding cavity of the target protein, which facilitated the formation of hydrogen bonds and hydrophobic interactions, thereby further strengthening molecular binding capability.

## 3. Materials and Methods

### 3.1. Instrumentation

All reagents and materials were commercially available and used as received without further purification. Melting points were determined using a B-545 melting point analyzer (BÜCHI Labortechnik AG, Flawil, St. Gallen, Switzerland) without calibration. All products were purified by column chromatography on 200–300 mesh silica gel, using petroleum ether and ethyl acetate as eluents. ^1^H NMR and ^13^C NMR spectra were recorded on a Bruker AV-400 or AV-500 MHz spectrometer (Bruker Corporation, Billerica, MA, USA) with CDCl_3_ as the solvent. Mass spectrometry (MS) analysis was performed on an Agilent 6545 Q-TOF liquid chromatography-mass spectrometer (Agilent Technologies, Santa Clara, CA, USA).

### 3.2. Synthesis

All substituted pyridines and 3-amino-1-methylpyrazole were commercially available and used without further purification. Compound **2** was synthesized following a reported protocol, whereas all other target compounds are novel and have not been described in the literature to date.

#### 3.2.1. General Synthesis of Compound **2**

Ethyl cyanoacetate and trimethyl orthoacetate were heated to 115 °C with distillation. After removing the light fraction, suction filtration afforded the methylenated product. At 0 °C, methylhydrazine sulfate and 2 eq triethylamine were added sequentially to anhydrous ethanol under stirring until complete dissolution. The methylenated product was then added, and the mixture was gradually heated to 80 °C. Upon reaction completion, the mixture was extracted with dichloromethane. The solvent was evaporated under reduced pressure, and the residue was left to stand to obtain the aminated product. The aminated product was dissolved in 2.5 eq of 40% aqueous tetrafluoroboric acid. A 25% aqueous sodium nitrite solution was added dropwise, and the reaction lasted for 2 h [33]. The solid was collected by suction filtration, washed with anhydrous ethanol and air-dried to yield compound **2**.

#### 3.2.2. General Synthesis of Compound **5a**–**5l**

Compound **2** (0.05 mol) and the corresponding substituted pyridine (65 mL) were charged into a reaction flask. The mixture was stirred at 50 °C until the reaction was complete (monitored by TLC). After cooling to room temperature, water (100 mL) was added, and the mixture was extracted with dichloromethane. The organic phase was washed successively with dilute aqueous HCl and saturated brine, dried over anhydrous Na_2_SO_4_, and concentrated under reduced pressure. The crude product was purified via silica gel column chromatography to afford the target compounds **5a**–**5l**.

#### 3.2.3. General Synthesis of Compound **6a**–**6l**

Compound **5** (6 mmol) was placed in a 100 mL two-necked flask and dissolved in 20 mL of ethanol under stirring. Subsequently, NaOH (24 mmol) and an appropriate amount of water were added, and the mixture was heated to reflux for 4 h. After cooling naturally to room temperature, concentrated hydrochloric acid was slowly dropped to adjust the pH of the reaction mixture to 2–3. The volatile solvent was evaporated under reduced pressure, and the obtained residue was redissolved in anhydrous methanol. Insoluble impurities were separated via filtration, and the filter cake was repeatedly rinsed with anhydrous methanol. All filtrates were collected and concentrated to yield carboxylic acid intermediates **6a**–**6l**. The obtained products were utilized directly for the subsequent reaction without additional purification.

#### 3.2.4. General Synthetic Procedure for Compounds **8a**–**8z**

Compound **6** (3 mmol) and substituted anthranilic acids (3.2 mmol) were placed in a 50 mL three-necked round-bottom flask, and acetonitrile (30 mL) was added with stirring. After the mixture was cooled to below 10 °C, 3-methylpyridine (15.6 mmol) was added dropwise. The mixture was stirred for 1 h, and then a solution of methanesulfonyl chloride (7.2 mmol) in acetonitrile was introduced. Stirring was continued below 10 °C for another 1 h, and the reaction mixture was then warmed to room temperature and stirred until the reaction was complete. The reaction mixture was poured into 100 mL dilute HCl, stirred vigorously for 30 min, and filtered to give the crude solid. The crude product was purified by silica gel column chromatography using EA and PE (V_EA_:V_PE_ = 1:3) to obtain pure compounds **8a**–**8z**.

***Ethyl 1,3-dimethyl-5-(pyridin-2-yl)-1H-pyrazole-4-carboxylate (3a)***-yellow oil, yield 15%, ^1^H NMR (400 MHz, DMSO-d*6*) δ 8.68 (dd, J = 4.9, 1.7 Hz, 1H), 8.61 (dd, J = 2.3, 0.9 Hz, 1H), 7.90 (dt, J = 7.8, 2.0 Hz, 1H), 7.53 (ddd, J = 7.9, 4.9, 0.9 Hz, 1H), 4.03–3.98 (m, 2H), 3.61 (d, J = 2.0 Hz, 3H), 2.38 (d, J = 2.4 Hz, 3H), 0.99 (t, J = 7.1 Hz, 3H). ^13^C NMR (101 MHz, Chloroform-*d*) δ 163.55, 151.26, 150.27, 150.16, 143.46, 137.76, 126.44, 123.16, 111.41, 59.90, 37.11, 14.13, 14.07. LCMS (ESI): calculated for C_13_H_15_N_3_O_2_[M + H]^+^ 246.1238, found 246.1232.

***Ethyl 1,3-dimethyl-5-(pyridin-3-yl)-1H-pyrazole-4-carboxylate (3b)***-yellow oil, yield 11%, ^1^H NMR (400 MHz, DMSO-d*6*) δ 8.70 (ddd, J = 4.9, 1.7, 1.0 Hz, 1H), 7.92 (td, J = 7.8, 1.8 Hz, 1H), 7.60 (dt, J = 7.8, 1.2 Hz, 1H), 7.49 (ddd, J = 7.7, 4.8, 1.2 Hz, 1H), 4.01 (q, J = 7.1 Hz, 2H), 3.64 (s, 3H), 2.37 (s, 3H), 1.00 (t, J = 7.1 Hz, 3H). ^13^C NMR (101 MHz, Chloroform-*d*) δ 163.52, 151.21, 150.30, 150.20, 143.46, 137.67, 128.98, 123.06, 111.36, 59.86, 37.07, 14.11, 14.04. LCMS (ESI): calculated for C_13_H_15_N_3_O_2_[M + H]^+^ 246.1232, found 246.1228.

***Ethyl 5-(2-bromopyridin-3-yl)-1,3-dimethyl-1H-pyrazole-4-carboxylate (5a)***-white solid, yield 13%, m.p. 133.8–134.1 °C, ^1^H NMR (400 MHz, Chloroform-*d*) δ 7.66 (t, J = 7.7 Hz, 1H), 7.55 (t, J = 7.5 Hz, 2H), 4.15 (q, J = 7.1 Hz, 2H), 3.79 (s, 3H), 2.49 (s, 3H), 1.16 (t, J = 7.1 Hz, 3H). ^13^C NMR (101 MHz, Chloroform-*d*) δ 163.28, 150.25, 149.69, 143.07, 141.06, 138.00, 127.87, 125.51, 110.76, 59.68, 37.40, 13.91, 13.88. LCMS (ESI): calculated for C_13_H_14_BrN_3_O_2_[M + Na]^+^ 346.0169, found 346.0170.

***Ethyl 5-(2-bromopyridin-4-yl)-1,3-dimethyl-1H-pyrazole-4-carboxylate (5b)***-yellow oil, yield 24%, ^1^H NMR (400 MHz, Chloroform-*d*) δ 8.31 (d, J = 2.2 Hz, 1H), 7.64–7.52 (m, 2H), 4.09 (q, J = 7.1 Hz, 2H), 3.65 (s, 3H), 2.46 (s, 3H), 1.11 (td, J = 7.1, 3.0 Hz, 3H). ^13^C NMR (101 MHz, Chloroform-*d*) δ 163.14, 150.48, 142.86, 142.04, 139.90, 134.48, 127.52, 125.44, 111.30, 59.82, 36.97, 13.97, 13.95. LCMS (ESI): calculated for C_13_H_14_BrN_3_O_2_[M + Na]^+^ 346.0169, found 346.0173.

***Ethyl 5-(6-bromopyridin-2-yl)-1,3-dimethyl-1H-pyrazole-4-carboxylate (5c)***-yellow oil, yield 20%, ^1^H NMR (400 MHz, Chloroform-*d*) δ 8.47 (dd, J = 4.8, 2.0 Hz, 1H), 7.60 (dd, J = 7.6, 2.0 Hz, 1H), 7.38 (dd, J = 7.6, 4.8 Hz, 1H), 4.04 (p, J = 7.0 Hz, 2H), 3.60 (s, 3H), 2.50 (d, J = 0.9 Hz, 3H), 1.01 (t, J = 7.1 Hz, 3H). ^13^C NMR (101 MHz, Chloroform-*d*) δ 163.15, 151.10, 150.77, 143.29, 143.16, 140.14, 129.58, 122.41, 111.54, 59.81, 36.96, 13.96, 13.94.LCMS(ESI): calculated for C_13_H_14_BrN_3_O_2_[M + Na]^+^ 346.0169, found 346.0176.

***Ethyl 5-(2-chloropyridin-3-yl)-1,3-dimethyl-1H-pyrazole-4-carboxylate (5d)***-white solid, yield 15%, m.p. 123.2–124.2 °C, ^1^H NMR (400 MHz, Chloroform-*d*) δ 7.74 (t, J = 7.8 Hz, 1H), 7.47 (d, J = 7.5 Hz, 1H), 7.38 (d, J = 8.0 Hz, 1H), 4.11 (q, J = 7.1 Hz, 2H), 3.75 (s, 3H), 2.45 (s, 3H), 1.13 (t, J = 7.1 Hz, 3H). ^13^C NMR (101 MHz, Chloroform-*d*) δ 163.49, 150.86, 150.46, 149.49, 143.40, 138.64, 125.38, 124.36, 110.99, 59.89, 37.62, 14.13, 14.09. LCMS (ESI): calculated for C_13_H_14_ClN_3_O_2_[M + Na]^+^ 302.0675, found 302.0674.

***Ethyl 5-(2-chloropyridin-4-yl)-1,3-dimethyl-1H-pyrazole-4-carboxylate (5e)***-yellow oil, yield 21%, ^1^H NMR (400 MHz, Chloroform-*d*) δ 8.46 (dd, J = 4.9, 2.0 Hz, 1H), 7.63 (dd, J = 7.6, 2.0 Hz, 1H), 7.33 (dd, J = 7.6, 4.8 Hz, 1H), 4.01 (qq, J = 7.3, 3.6 Hz, 2H), 3.58 (s, 3H), 2.46 (s, 3H), 0.98 (t, J = 7.1 Hz, 3H). ^13^C NMR (101 MHz, Chloroform-d) δ 163.07, 151.01, 150.82, 150.49, 141.80, 140.68, 126.49, 122.07, 111.50, 59.71, 36.81, 13.87, 13.85. LCMS (ESI): calculated for C_13_H_14_ClN_3_O_2_[M + Na]^+^ 302.0675, found 302.0680.

***Ethyl 5-(2-chloropyridin-5-yl)-1,3-dimethyl-1H-pyrazole-4-carboxylate (5f)***-yellow oil, yield 23%, ^1^H NMR (400 MHz, Chloroform-*d*) δ 7.73 (t, J = 7.8 Hz, 1H), 7.46 (d, J = 7.5 Hz, 1H), 7.37 (d, J = 8.0 Hz, 1H), 4.11 (q, J = 7.1 Hz, 2H), 3.74 (s, 3H), 2.45 (s, 3H), 1.12 (t, J = 7.1 Hz, 3H). 13C NMR (101 MHz, Chloroform-*d*) δ 163.24, 152.23, 151.10, 150.18, 142.12, 140.28, 134.55, 125.13, 123.78, 111.40, 59.89, 37.01, 14.03. LCMS (ESI): calculated for C_13_H_14_ClN_3_O_2_[M + Na]^+^ 302.0675, found 302.0677.

***Ethyl 5-(3-bromopyridin-4-yl)-1,3-dimethyl-1H-pyrazole-4-carboxylate (5g)***-white solid, yield 21%, m.p. 123.6–124.4 °C, ^1^H NMR (400 MHz, Chloroform-*d*) δ 8.74 (t, J = 2.0 Hz, 1H), 7.89 (dt, J = 8.3, 2.0 Hz, 1H), 7.44 (dd, J = 8.4, 1.5 Hz, 1H), 4.12 (qd, J = 7.1, 1.5 Hz, 2H), 3.72 (d, J = 1.6 Hz, 3H), 2.45 (d, J = 1.6 Hz, 3H), 1.14 (td, J = 7.2, 1.5 Hz, 3H). ^13^C NMR (101 MHz, Chloroform-*d*) δ 163.53, 150.43, 150.41, 147.58, 143.90, 138.60, 128.03, 121.16, 110.72, 59.87, 37.53, 14.18, 14.13. LCMS (ESI): calculated for C_13_H_14_BrN_3_O_2_[M + Na]^+^ 346.0169, found 346.0178.

***Ethyl 5-(5-bromopyridin-3-yl)-1,3-dimethyl-1H-pyrazole-4-carboxylate (5h)***-yellow oil, yield 24%, ^1^H NMR (400 MHz, Chloroform-*d*) δ 8.75 (d, J = 2.2 Hz, 1H), 8.49 (d, J = 1.8 Hz, 1H), 7.85 (t, J = 2.0 Hz, 1H), 4.10 (q, J = 7.1 Hz, 2H), 3.67 (s, 3H), 2.49 (s, 3H), 1.10 (t, J = 7.1 Hz, 3H). ^13^C NMR (101 MHz, Chloroform-*d*) δ 163.23, 151.39, 151.34, 148.41, 141.71, 140.19, 127.82, 120.12, 111.63, 60.00, 37.22, 14.07, 14.05. LCMS (ESI): calculated for C_13_H_14_BrN_3_O_2_[M + Na]^+^ 346.0169, found 346.0181.

***Ethyl 5-(5-bromopyridin-2-yl)-1,3-dimethyl-1H-pyrazole-4-carboxylate (5i)***-yellow oil, yield 11%, ^1^H NMR (400 MHz, Chloroform-*d*) δ 8.86 (s, 1H), 8.62 (d, J = 4.9 Hz, 1H), 7.23 (d, J = 4.9 Hz, 1H), 4.06 (pd, J = 7.1, 4.3 Hz, 2H), 3.58 (s, 3H), 2.50 (s, 3H), 1.01 (t, J = 7.1 Hz, 3H). ^13^C NMR (101 MHz, Chloroform-*d*) δ 162.98, 152.25, 151.12, 148.22, 142.60, 140.23, 125.95, 122.30, 111.40, 59.94, 36.99, 13.87, 13.84. LCMS (ESI): calculated for C_13_H_14_BrN_3_O_2_[M + Na]^+^ 346.0169, found 346.0184.

***Ethyl 5-(3-chloropyridin-4-yl)-1,3-dimethyl-1H-pyrazole-4-carboxylate (5j)***-yellow oil, yield 10%, ^1^H NMR (400 MHz, Chloroform-*d*) δ 8.74 (s, 1H), 8.60 (d, J = 4.9 Hz, 1H), 7.24 (d, J = 4.8 Hz, 1H), 4.06 (qd, J = 7.1, 3.0 Hz, 2H), 3.59 (s, 3H), 2.50 (s, 3H), 1.04 (d, J = 7.2 Hz, 3H). ^13^C NMR (101 MHz, Chloroform-*d*) δ 163.05, 151.25, 149.92, 147.71, 141.07, 138.02, 131.93, 125.82, 111.66, 60.00, 37.00, 13.92, 13.89. LCMS (ESI): calculated for C_13_H_14_ClN_3_O_2_[M + Na]^+^ 302.0675, found 302.0665.

***Ethyl 5-(5-chloropyridin-3-yl)-1,3-dimethyl-1H-pyrazole-4-carboxylate (5k***)-yellow oil, yield 25%, ^1^H NMR (400 MHz, Chloroform-*d*) δ 8.66 (d, J = 2.3 Hz, 1H), 8.45 (d, J = 1.8 Hz, 1H), 7.71 (t, J = 1.9 Hz, 1H), 4.11 (q, J = 7.1 Hz, 2H), 3.68 (s, 3H), 2.49 (s, 3H), 1.10 (t, J = 7.1 Hz, 3H). ^13^C NMR (101 MHz, Chloroform-*d*) δ 163.26, 151.40, 149.23, 148.09, 141.80, 137.43, 131.66, 127.46, 111.66, 60.02, 37.23, 14.08, 14.06. LCMS (ESI): calculated for C_13_H_14_ClN_3_O_2_[M + Na]^+^ 302.0675, found 302.0668.

***Ethyl 5-(5-chloropyridin-2-yl)-1,3-dimethyl-1H-pyrazole-4-carboxylate (5l)***-white solid, yield 23%, m.p. 124.1–124.9 °C, ^1^H NMR (400 MHz, Chloroform-*d*) δ 8.64 (d, J = 2.4 Hz, 1H), 7.73 (dd, J = 8.4, 2.5 Hz, 1H), 7.49 (d, J = 8.4 Hz, 1H), 4.11 (q, J = 7.1 Hz, 2H), 3.71 (s, 3H), 2.45 (s, 3H), 1.14 (t, J = 7.1 Hz, 3H). ^13^C NMR (101 MHz, Chloroform-*d*) δ 163.55, 150.41, 148.25, 147.22, 143.89, 135.74, 132.33, 127.62, 110.74, 59.87, 37.51, 14.17, 14.12. LCMS (ESI): calculated for C_13_H_14_ClN_3_O_2_[M + Na]^+^ 302.0675, found 302.0671.

***4-methoxy-3-(1-methyl-1H-pyrazol-5-yl)pyridine (5o)***-yellow oil, yield 12%, ^1^H NMR (400 MHz, DMSO-*d6*) δ 8.88 (s, 1H), 8.38 (d, J = 5.8 Hz, 1H), 7.75 (d, J = 2.3 Hz, 1H), 7.14 (d, J = 5.8 Hz, 1H), 6.70 (d, J = 2.2 Hz, 1H), 3.93 (s, 3H), 3.91 (s, 3H). ^13^C NMR (101 MHz, DMSO-d6) δ 161.81, 149.77, 148.02, 144.15, 131.54, 118.10, 107.44, 106.91, 55.62, 38.69. LCMS (ESI): calculated for C_10_H_11_N_3_O[M + Na]^+^ 212.0795, found 212.0798.

***2-(1-methyl-1H-pyrazol-3-yl)pyridine (5p)***-yellow oil, yield 12%, ^1^H NMR (400 MHz, DMSO-*d6*) δ 8.55 (ddd, J = 4.9, 1.8, 1.0 Hz, 1H), 7.89 (dt, J = 7.9, 1.2 Hz, 1H), 7.82–7.72 (m, 2H), 7.27 (ddd, J = 7.5, 4.8, 1.3 Hz, 1H), 6.79 (d, J = 2.2 Hz, 1H), 3.91 (s, 3H). LCMS (ESI): calculated for C_9_H_9_N_3_[M + Na]^+^ 182.0689, found 182.0695.

***3-(1-methyl-1H-pyrazol-3-yl)pyridine (5q)***-yellow oil, yield 9%, ^1^H NMR (400 MHz, DMSO-*d6*) δ 9.18–8.86 (m, 1H), 8.53 (ddd, J = 31.3, 4.6, 1.6 Hz, 1H), 8.13 (dt, J = 8.0, 1.9 Hz, 1H), 7.91–7.73 (m, 1H), 7.42 (ddd, J = 8.0, 4.8, 0.9 Hz, 1H), 6.81 (d, J = 2.3 Hz, 1H), 3.91 (s, 3H). LCMS (ESI): calculated for C_9_H_9_N_3_[M + Na]^+^ 182.0689, found 182.0697.

***5-chloro-2-(1-methyl-1H-pyrazol-3-yl)pyridine (5r)***-yellow oil, yield 14%, ^1^H NMR (400 MHz, DMSO-d6) δ 8.60 (dd, J = 2.2, 1.0 Hz, 1H), 7.97–7.88 (m, 2H), 7.80 (d, J = 2.3 Hz, 1H), 6.79 (d, J = 2.2 Hz, 1H), 3.92 (s, 3H). ^13^C NMR (101 MHz, DMSO-*d6*) δ 148.17, 137.13, 133.21, 120.77, 104.57. LCMS (ESI): calculated for C_9_H_8_ClN_3_[M + Na]^+^ 216.0299, found 216.0297.

***3-chloro-2-(1-methyl-1H-pyrazol-3-yl)pyridine (5s)***-yellow oil, yield 12%, ^1^H NMR (400 MHz, DMSO-*d6*) δ 8.57 (dd, J = 4.5, 1.5 Hz, 1H), 7.98 (dd, J = 8.1, 1.5 Hz, 1H), 7.80 (d, J = 2.2 Hz, 1H), 7.37 (dd, J = 8.1, 4.6 Hz, 1H), 6.77 (d, J = 2.3 Hz, 1H), 3.93 (s, 3H). LCMS (ESI): calculated for C_9_H_8_ClN_3_[M + Na]^+^ 216.0299, found 216.0295.

***2-[5-(5-Bromopyridin-3-yl)-1,3-dimethyl-1H-pyrazol-4-yl]-6,8-dimethyl-4H-3,1-benzoxazin-4-one (8a)***-white solid, yield 59%, m.p. 178.6–179.1 °C, ^1^H NMR (400 MHz, Chloroform-*d*) δ 8.81 (s, 1H), 8.58 (s, 1H), 7.96 (t, J = 2.0 Hz, 1H), 7.82–7.73 (m, 1H), 7.40–7.35 (m, 1H), 3.73 (s, 3H), 2.68 (s, 3H), 2.38 (s, 3H), 2.12 (s, 3H). ^13^C NMR (101 MHz, Chloroform-*d*) δ 154.23, 152.80, 151.46, 150.03, 148.58, 143.46, 140.73, 140.39 (2C), 138.61, 137.44, 135.26, 129.35, 125.71 (2C), 116.37, 37.37, 21.32, 17.01, 14.91. LCMS (ESI): calculated for C_20_H_17_^79^BrN_4_O_2_[M+H]^+^ 425.0608, found 425.0607, calculated for C_20_H_17_^81^BrN_4_O_2_[M+H]^+^ 427.0588, found 427.0575.

***2-[5-(5-Bromopyridin-3-yl)-1,3-dimethyl-1H-pyrazol-4-yl]-8-fluoro-4H-3,1-benzoxazin-4-one (8b)***-white solid, yield 52%, m.p. 211.1–211.6 °C, ^1^H NMR (400 MHz, Chloroform-*d*) δ 8.82 (d, J = 2.3 Hz, 1H), 8.56 (d, J = 1.8 Hz, 1H), 8.05 (t, J = 2.1 Hz, 1H), 7.90 (dt, J = 7.8, 1.2 Hz, 1H), 7.44 (ddd, J = 9.5, 8.1, 1.4 Hz, 1H), 7.34 (td, J = 8.0, 4.6 Hz, 1H), 3.75 (s, 3H), 2.68 (s, 3H). ^13^C NMR (101 MHz, Chloroform-*d*) δ 156.23, 151.59, 150.48, 157.84, 154.35, 148.34, 141.33, 140.69, 136.33, 128.07–126.88 (m), 123.82, 122.32, 120.21, 118.26, 111.14, 37.38, 14.66. LCMS (ESI): calculated for C_18_H_12_^79^BrFN_4_O_2_[M + Na]^+^ 437.0020, found 437.0037, calculated for C_18_H_12_^81^BrFN_4_O_2_[M + Na]^+^ 439.0000, found 439.0018.

***2-[5-(5-Bromopyridin-3-yl)-1,3-dimethyl-1H-pyrazol-4-yl]-6-bromo-8-methyl-4H-3,1-benzoxazin-4-one (8c)***-yellow solid, yield 42%, m.p. 186.7–187.1 °C, ^1^H NMR (400 MHz, Chloroform-*d*) δ 8.80 (d, J = 2.2 Hz, 1H), 8.56 (d, J = 1.9 Hz, 1H), 8.04 (d, J = 2.3 Hz, 1H), 7.94 (t, J = 2.0 Hz, 1H), 7.63–7.58 (m, 1H), 3.72 (s, 3H), 2.64 (s, 3H), 2.10 (s, 3H). ^13^C NMR (101 MHz, Chloroform-*d*) δ 158.18, 153.37, 151.55, 150.27, 148.45, 144.56, 141.09, 140.22, 139.87, 137.78, 128.32, 127.94, 120.52, 120.18, 117.74, 111.41, 37.38, 16.89, 14.96. LCMS (ESI): calculated for C_19_H_14_^79^Br_2_N_4_O_2_[M + Na]^+^ 510.9376, found 510.9381, calculated for C_19_H_14_^81^Br_2_N_4_O_2_[M + Na]^+^ 512.9356, found 512.9370.

***2-[5-(5-Bromopyridin-3-yl)-1,3-dimethyl-1H-pyrazol-4-yl]-6-chloro-8-methyl-4H-3,1-benzoxazin-4-one (8d)***-yellow solid, yield 39%, m.p. 185.6–185.9 °C, ^1^H NMR (400 MHz, Chloroform-*d*) δ 8.81 (d, J = 2.2 Hz, 1H), 8.56 (d, J = 1.9 Hz, 1H), 7.95 (t, J = 2.1 Hz, 1H), 7.89 (t, J = 2.8 Hz, 1H), 7.50–7.44 (m, 1H), 3.72 (s, 3H), 2.65 (d, J = 3.0 Hz, 3H), 2.11 (d, J = 2.3 Hz, 3H). ^13^C NMR (101 MHz, Chloroform-*d*) δ 158.40, 153.26, 151.55, 150.26, 148.46, 144.22, 141.07, 140.25, 137.66, 137.11, 132.47, 127.96, 125.22, 120.53, 117.49, 111.41, 37.38, 16.98, 14.95. LCMS (ESI): calculated for C_19_H_14_^79^BrClN_4_O_2_[M + H]^+^ 445.0662, found 445.0661, calculated for C_19_H_14_^81^BrClN_4_O_2_[M + H]^+^ 447.0041, found 447.0036.

***2-[5-(5-Bromopyridin-3-yl)-1,3-dimethyl-1H-pyrazol-4-yl]-8-methyl-4H-3,1-benzoxazin-4-one (8e)***-white solid, yield 48%, m.p. 210.3–210.8 °C, ^1^H NMR (400 MHz, Chloroform-*d*) δ 8.84–8.77 (m, 1H), 8.61–8.53 (m, 1H), 7.98–7.91 (m, 2H), 7.52 (dt, J = 7.1, 1.3 Hz, 1H), 7.27 (d, J = 7.7 Hz, 1H), 3.72 (s, 3H), 2.67 (s, 3H), 2.14 (s, 3H). ^13^C NMR (101 MHz, Chloroform-*d*) δ 159.51, 152.98, 151.47, 150.14, 148.51, 145.54, 140.90, 140.32, 137.20, 135.46, 128.09, 127.12, 126.06, 120.52, 116.55, 111.74, 37.36, 17.09, 14.91. LCMS (ESI): calculated for C_19_H_15_^79^BrN_4_O_2_[M + H]^+^ 411.0452, found 411.0451, calculated for C_19_H_15_^81^BrN_4_O_2_[M + H]^+^ 413.0431, found 413.0432.

***2-[5-(5-chloropyridin-3-yl)-1,3-dimethyl-1H-pyrazol-4-yl]-6-chloro-8-methyl-4H-3,1-benzoxazin-4-one (8f)***-yellow solid, yield 39%, m.p. 185.1–185.7 °C, ^1^H NMR (400 MHz, Chloroform-*d*) δ 8.71 (d, J = 2.3 Hz, 1H), 8.53 (d, J = 1.9 Hz, 1H), 7.93–7.88 (m, 1H), 7.80 (t, J = 2.1 Hz, 1H), 7.48 (dd, J = 2.4, 1.0 Hz, 1H), 3.73 (s, 3H), 2.67 (s, 3H), 2.12 (s, 3H). ^13^C NMR (101 MHz, Chloroform-*d*) δ 158.40, 153.25, 150.29, 149.43, 148.14, 144.22, 141.09, 137.64, 137.49, 137.11, 132.48, 132.00, 127.56, 125.22, 117.49, 111.41, 37.37, 16.94, 14.95. LCMS (ESI): calculated for C_19_H_14_C_l2_N_4_O_2_[M + Na]^+^ 423.0387, found 423.0405.

***2-[5-(5-chloropyridin-3-yl)-1,3-dimethyl-1H-pyrazol-4-yl]-6-bromo-8-methyl-4H-3,1-benzoxazin-4-one (8g)***-yellow solid, yield 32%, m.p. 183.4–184.1 °C, ^1^H NMR (400 MHz, Chloroform-*d*) δ 8.71 (d, J = 2.3 Hz, 1H), 8.53 (d, J = 1.9 Hz, 1H), 8.10–8.03 (m, 1H), 7.80 (t, J = 2.1 Hz, 1H), 7.63 (dd, J = 2.2, 1.0 Hz, 1H), 3.73 (s, 3H), 2.66 (s, 3H), 2.11 (s, 3H). ^13^C NMR (101 MHz, Chloroform-*d*) δ 158.28, 153.42, 150.36, 149.45, 148.13, 144.64, 141.14, 139.96, 137.82, 137.54, 132.05, 128.43,127.60, 120.27, 117.83, 111.52, 37.42, 16.90, 15.00. LCMS (ESI): calculated for C_19_H_14_^79^BrClN_4_O_2_[M + Na]^+^ 466.9881, found 466.9891, calculated for C_19_H_14_^81^BrClN_4_O_2_[M + Na]^+^ 468.9861, found 468.9873.

***2-[5-(5-Chloropyridin-3-yl)-1,3-dimethyl-1H-pyrazol-4-yl]-4H-3,1-benzoxazin-4-one (8h)***-white solid, yield 51%, m.p. 207.1–207.4 °C, ^1^H NMR (400 MHz, Chloroform-*d*) δ 8.72 (d, J = 2.3 Hz, 1H), 8.55 (d, J = 1.9 Hz, 1H), 8.11 (dd, J = 7.9, 1.5 Hz, 1H), 7.86 (t, J = 2.1 Hz, 1H), 7.71 (ddd, J = 8.1, 7.3, 1.5 Hz, 1H), 7.41 (td, J = 7.6, 1.1 Hz, 1H), 7.32 (dd, J = 8.2, 1.1 Hz, 1H), 3.75 (s, 3H), 2.68 (s, 3H). ^13^C NMR (101 MHz, Chloroform-*d*) δ 159.08, 153.97, 150.34, 149.47, 148.36, 147.14, 141.00, 137.86, 136.51, 131.71, 128.54, 127.76, 127.20, 126.81, 116.76, 111.45, 37.44, 14.76. LCMS (ESI): calculated for C_18_H_13_ClN_4_O_2_[M + Na]^+^ 375.0620, found 375.0634.

***2-[5-(5-Chloropyridin-3-yl)-1,3-dimethyl-1H-pyrazol-4-yl]-8-methyl-4H-3,1-benzoxazin-4-one (8i)***-white solid, yield 43%, m.p. 210.6–210.9 °C, ^1^H NMR (400 MHz, Chloroform-*d*) δ 8.70 (d, J = 2.3 Hz, 1H), 8.54 (d, J = 1.9 Hz, 1H), 7.94 (dd, J = 7.9, 1.5 Hz, 1H), 7.82 (t, J = 2.1 Hz, 1H), 7.55–7.50 (m, 1H), 7.26 (t, J = 7.6 Hz, 1H), 3.72 (s, 3H), 2.67 (s, 3H), 2.14 (s, 3H). ^13^C NMR (101 MHz, Chloroform-*d*) δ 159.54, 152.99, 150.18, 149.35, 148.18, 145.55, 140.92, 137.62, 137.23, 135.46, 132.00, 127.70, 127.15, 126.09, 116.56, 111.77, 37.37, 17.06, 14.92. LCMS (ESI): calculated for C_19_H_15_ClN_4_O_2_[M + Na]^+^ 389.0776, found 389.0778.

***2-[5-(5-Chloropyridin-3-yl)-1,3-dimethyl-1H-pyrazol-4-yl]-6,8-dimethyl-4H-3,1-benzoxazin-4-one (8j)***-white solid, yield 61%, m.p. 177.4–177.9 °C, ^1^H NMR (400 MHz, Chloroform-*d*) δ 8.71 (s, 1H), 8.55 (s, 1H), 7.82 (d, J = 1.8 Hz, 1H), 7.79–7.75 (m, 1H), 7.38–7.34 (m, 1H), 3.73 (s, 3H), 2.68 (s, 3H), 2.37 (s, 3H), 2.12 (s, 3H). ^13^C NMR (101 MHz, Chloroform-*d*) δ 159.78, 152.31, 150.04, 149.35, 148.25, 143.45, 140.74, 138.60 (2C), 137.65, 137.43, 135.23, 125.70 (2C), 116.36, 111.92, 37.39, 21.31, 16.96, 14.90. LCMS (ESI): calculated for C_20_H_17_ClN_4_O_2_[M + Na]^+^ 403.0933, found 403.0957.

***2-[5-(5-Chloropyridin-2-yl)-1,3-dimethyl-1H-pyrazol-4-yl]-6,8-dimethyl-4H-3,1-benzoxazin-4-one (8k)***-white solid, yield 57%, m.p. 177.1–177.8 °C, ^1^H NMR (400 MHz, Chloroform-*d*) δ 8.72 (dd, J = 2.5, 0.8 Hz, 1H), 7.81 (dd, J = 8.4, 2.5 Hz, 1H), 7.74 (d, J = 1.9 Hz, 1H), 7.56 (dd, J = 8.3, 0.8 Hz, 1H), 7.40–7.34 (m, 1H), 3.79 (s, 3H), 2.66 (s, 3H), 2.37 (s, 3H), 2.23 (s, 3H). ^13^C NMR (101 MHz, Chloroform-*d*) δ 159.94, 152.51, 149.69, 148.72, 147.54, 143.60, 142.73, 138.52, 137.28, 136.17, 135.19, 132.56, 127.48, 125.63, 116.33, 111.16, 37.66, 21.30, 17.00, 14.82. LCMS (ESI): calculated for C_20_H_17_ClN_4_O_2_[M + Na]^+^ 403.0933, found 403.0949.

***2-[5-(5-chloropyridin-2-yl)-1,3-dimethyl-1H-pyrazol-4-yl]-6-chloro-8-methyl-4H-3,1-benzoxazin-4-one (8l)***-yellow solid, yield 34%, m.p. 183.4–184.1 °C, ^1^H NMR (400 MHz, Chloroform-d) δ 8.73 (dd, J = 2.5, 0.7 Hz, 1H), 7.91 (d, J = 2.4 Hz, 1H), 7.87–7.80 (m, 1H), 7.57–7.50 (m, 2H), 3.78 (s, 3H), 2.67 (s, 3H), 2.25 (s, 3H). ^13^C NMR (101 MHz, Chloroform-d) δ 158.68, 153.52, 150.05, 148.88, 147.40, 144.47, 143.15, 137.68, 137.14, 136.29, 132.77, 132.43, 127.34, 125.28, 117.57, 110.80, 37.69, 17.05, 14.91. LCMS (ESI): calculated for C_19_H_15_ClN_4_O_2_[M + Na]^+^ 423.0387, found 423.0408.

***2-[5-(3-Chloropyridin-4-yl)-1,3-dimethyl-1H-pyrazol-4-yl]-6,8-dimethyl-4H-3,1-benzoxazin-4-one (8m)***-white solid, yield 53%, m.p. 178.5–178.9 °C, ^1^H NMR (400 MHz, Chloroform-*d*) δ 8.80 (s, 1H), 8.66 (d, J = 4.7 Hz, 1H), 7.77–7.70 (m, 1H), 7.35–7.30 (m, 2H), 3.66 (s, 3H), 2.69 (s, 3H), 2.35 (s, 3H), 2.00 (s, 3H). ^13^C NMR (101 MHz, Chloroform-d) δ 159.75, 152.05, 150.11, 149.84, 148.05, 143.47, 139.97, 138.49 (2C), 138.22, 137.30, 135.16, 125.62 (2C), 116.20, 111.83, 37.09, 21.27, 16.55, 14.78. LCMS (ESI): calculated for C_20_H_17_ClN_4_O_2_[M + Na]^+^ 403.0933, found 403.0951.

***2-[5-(3-Bromopyridin-4-yl)-1,3-dimethyl-1H-pyrazol-4-yl]-6,8-dimethyl-4H-3,1-benzoxazin-4-one (8n)***-white solid, yield 51%, m.p. 179.4–179.8 °C, ^1^H NMR (400 MHz, Chloroform-*d*) δ 8.85–8.80 (m, 1H), 7.96 (dd, J = 8.3, 2.4 Hz, 1H), 7.77–7.72 (m, 1H), 7.51 (dd, J = 8.4, 0.8 Hz, 1H), 7.39–7.36 (m, 1H), 3.79 (s, 3H), 2.66 (s, 3H), 2.38 (s, 3H), 2.23 (s, 3H). 13C NMR (101 MHz, Chloroform-*d*) δ 159.93, 152.52, 150.92, 149.71, 147.94, 143.61, 142.76, 139.07, 138.53, 137.30, 135.21, 127.90, 125.65, 121.37, 116.36, 111.19, 37.67, 21.30, 16.99, 14.78. LCMS (ESI): calculated for C_20_H_17_^79^BrN_4_O_2_[M + Na]^+^ 447.0428, found 447.0434, calculated for C_20_H_17_^81^BrN_4_O_2_[M + Na]^+^ 449.0407, found 449.0418.

***2-[5-(3-Bromopyridin-4-yl)-1,3-dimethyl-1H-pyrazol-4-yl]-8-methyl-4H-3,1-benzoxazin-4-one (8o)***-white solid, yield 48%, m.p. 209.2–209.7 °C, ^1^H NMR (400 MHz, Chloroform-*d*) δ 8.83 (dd, J = 2.4, 0.8 Hz, 1H), 7.95 (ddd, J = 9.5, 8.2, 2.0 Hz, 2H), 7.60–7.46 (m, 2H), 7.27 (t, J = 7.6 Hz, 1H), 3.78 (s, 3H), 2.67 (s, 3H), 2.26 (s, 3H). ^13^C NMR (101 MHz, Chloroform-*d*) δ 159.69, 153.20, 150.92, 149.85, 147.86, 145.72, 142.95, 139.07, 137.15, 135.45, 127.85, 127.03, 126.03, 121.40, 116.57, 111.05, 37.65, 17.09, 14.82. LCMS (ESI): calculated for C_19_H_15_^79^BrN_4_O_2_[M + Na]^+^ 433.0271, found 433.0290, calculated for C_19_H_15_^81^BrN_4_O_2_[M + Na]^+^ 435.0251, found 435.0269.

***2-[5-(3-Bromopyridin-4-yl)-1,3-dimethyl-1H-pyrazol-4-yl]-6-chloro-8-methyl-4H-3,1-benzoxazin-4-one (8p)***-yellow solid, yield 36%, m.p. 188.7–189.4 °C, ^1^H NMR (400 MHz, Chloroform-*d*) δ 8.83 (dd, J = 2.4, 0.7 Hz, 1H), 7.98 (dd, J = 8.3, 2.4 Hz, 1H), 7.91 (dd, J = 2.4, 0.7 Hz, 1H), 7.52–7.46 (m, 2H), 3.78 (s, 3H), 2.66 (s, 3H), 2.24 (s, 3H). ^13^C NMR (101 MHz, Chloroform-*d*) δ 158.65, 153.49, 151.05, 150.04, 147.75, 144.46, 143.16, 139.19, 137.68, 137.15, 132.45, 127.76, 125.28, 121.56, 117.57, 110.81, 37.70, 17.03, 14.89. LCMS (ESI): calculated for C_19_H_14_^79^BrClN_4_O_2_[M + Na]^+^ 466.9881, found 466.9893, calculated for C_19_H_14_^81^BrClN_4_O_2_[M + Na]^+^ 468.9861, found 468.9873.

***2-[5-(2-Chloropyridin-3-yl)-1,3-dimethyl-1H-pyrazol-4-yl]-6-bromo-8-methyl-4H-3,1-benzoxazin-4-one (8q)***-yellow solid, yield 37%, m.p. 185.4–185.7 °C, ^1^H NMR (400 MHz, Chloroform-*d*) δ 8.06 (dd, J = 2.3, 0.7 Hz, 1H), 7.82 (t, J = 7.8 Hz, 1H), 7.65 (dd, J = 2.4, 0.9 Hz, 1H), 7.49 (ddd, J = 15.5, 7.9, 0.9 Hz, 2H), 3.80 (s, 3H), 2.65 (s, 3H), 2.24 (d, J = 0.9 Hz, 3H). ^13^C NMR (101 MHz, Chloroform-*d*) δ 158.42, 153.52, 151.48, 150.01, 149.64, 144.84, 142.73, 139.88, 139.12, 137.81, 128.39, 125.10, 124.80, 120.11, 117.84, 110.97, 37.74, 17.00, 14.92. LCMS (ESI): calculated for C_19_H_14_^79^BrClN_4_O_2_[M + Na]^+^ 466.9881, found 466.9891, calculated for C_19_H_14_^81^BrClN_4_O_2_[M + Na]^+^ 468.9861, found 468.9872.

***2-[5-(2-Chloropyridin-3-yl)-1,3-dimethyl-1H-pyrazol-4-yl]-4H-3,1-benzoxazin-4-one (8r)***-white solid, yield 48%, m.p. 206.8–207.4 °C, ^1^H NMR (400 MHz, Chloroform-*d*) δ 8.54 (dd, J = 4.9, 2.0 Hz, 1H), 8.05 (dd, J = 7.9, 1.5 Hz, 1H), 7.72 (dd, J = 7.6, 2.0 Hz, 1H), 7.66 (td, J = 7.8, 1.5 Hz, 1H), 7.46 (dd, J = 7.6, 4.8 Hz, 1H), 7.39–7.33 (m, 1H), 7.25 (d, J = 7.7 Hz, 1H), 3.68 (s, 3H), 2.68 (s, 3H). ^13^C NMR (101 MHz, Chloroform-*d*) δ 158.97, 153.87, 150.98, 150.08, 147.20, 143.49, 142.27, 140.45, 136.31, 129.29, 128.35, 127.49, 126.69, 122.56, 116.56, 111.18, 37.09, 14.67. LCMS (ESI): calculated for C_18_H_13_ClN_4_O_2_[M + Na]^+^ 375.0620, found 375.0634.

***2-[5-(2-Bromopyridin-3-yl)-1,3-dimethyl-1H-pyrazol-4-yl]-8-fluoro-4H-3,1-benzoxazin-4-one (8s)***-white solid, yield 42%, m.p. 210.4–210.9 °C, ^1^H NMR (400 MHz, Chloroform-*d*) δ 7.90 (dt, J = 7.9, 1.2 Hz, 1H), 7.75–7.64 (m, 2H), 7.61 (dd, J = 7.8, 1.1 Hz, 1H), 7.44 (ddd, J = 9.6, 8.1, 1.4 Hz, 1H), 7.38–7.32 (m, 1H), 3.84 (s, 3H), 2.67 (s, 3H). ^13^C NMR (101 MHz, Chloroform-*d*) δ 158.10, 156.39, 154.65, 150.20, 149.55, 142.89, 141.58, 138.52, 136.62, 128.56, 127.55, 126.10, 123.92, 122.33, 118.42, 110.67, 37.89, 14.72. LCMS (ESI): calculated for C_18_H_12_^79^BrFN_4_O_2_[M+Na]^+^ 437.0020, found 437.0036, calculated for C_18_H_12_^81^BrFN_4_O_2_[M+Na]^+^ 439.0000, found 439.0018.

***2-[5-(2-Bromopyridin-3-yl)-1,3-dimethyl-1H-pyrazol-4-yl]-6-bromo-8-methyl-4H-3,1-benzoxazin-4-one (8t)***-yellow solid, yield 37%, m.p. 186.5–186.9 °C. ^1^H NMR (400 MHz, Chloroform-*d*) δ 8.09–8.02 (m, 1H), 7.71 (t, J = 7.8 Hz, 1H), 7.66 (dd, J = 2.3, 1.0 Hz, 1H), ·7.62 (dd, J = 8.0, 1.0 Hz, 1H), 7.54 (dd, J = 7.5, 1.0 Hz, 1H), 3.80 (s, 3H), 2.65 (s, 3H), 2.24 (s, 3H). ^13^C NMR (101 MHz, Chloroform-*d*) δ 158.41, 153.52, 150.05, 149.99, 144.84, 142.64, 141.91, 139.88, 138.72, 137.82, 128.55, 128.39, 125.47, 120.12, 117.84, 110.97, 37.75, 17.02, 14.92. LCMS (ESI): calculated for C_19_H_14_^79^Br_2_N_4_O_2_[M+Na]^+^ 510.9376, found 510.9394, calculated for C_19_H_14_^81^Br_2_N_4_O_2_[M+Na]^+^ 512.9356, found 512.9373.

***2-[5-(2-Bromopyridin-3-yl)-1,3-dimethyl-1H-pyrazol-4-yl]-6,8-dimethyl-4H-3,1-benzoxazin-4-one (8u)***-white solid, yield 42%, m.p. 178.5–178.9 °C, ^1^H NMR (400 MHz, Chloroform-*d*) δ 7.77–7.72 (m, 1H), 7.70 (t, J = 7.7 Hz, 1H), 7.58 (ddd, J = 12.8, 7.8, 1.0 Hz, 2H), 7.37 (d, J = 2.5 Hz, 1H), 3.81 (s, 3H), 2.66 (s, 3H), 2.37 (s, 3H), 2.23 (s, 3H). ^13^C NMR (101 MHz, Chloroform-*d*) δ 159.89, 152.41, 150.21, 149.64, 143.63, 142.20, 141.74, 138.63, 138.50, 137.26, 135.21, 128.36, 125.63 (2C), 116.36, 111.33, 37.74, 21.30, 17.08, 14.82. LCMS (ESI): calculated for C_20_H_17_^79^BrN_4_O_2_[M + Na]^+^ 447.0428, found 447.0447, calculated for C_20_H_17_^81^BrN_4_O_2_[M + Na]^+^ 449.0407, found 449.0429.

***2-[5-(2-Bromopyridin-4-yl)-1,3-dimethyl-1H-pyrazol-4-yl]-6,8-dimethyl-4H-3,1-benzoxazin-4-one (8v)***-white solid, yield 59%, m.p. 177.9–178.4 °C, ^1^H NMR (400 MHz, Chloroform-*d*) δ 8.42 (dd, J = 2.2, 1.1 Hz, 1H), 7.79–7.72 (m, 1H), 7.71–7.62 (m, 2H), 7.41–7.34 (m, 1H), 3.72 (s, 3H), 2.67 (s, 3H), 2.37 (s, 3H), 2.15 (s, 3H). ^13^C NMR (101 MHz, Chloroform-*d*) δ 159.80, 152.36, 150.85, 150.08, 143.42, 143.14, 141.05, 140.10, 138.61, 137.42, 135.26, 128.03, 125.95, 125.67, 116.32, 111.85, 37.28, 21.31, 16.89, 14.86. LCMS (ESI): calculated for C_20_H_17_^79^BrN_4_O_2_[M + Na]^+^ 447.0428, found 447.0448, calculated for C_20_H_17_^81^BrN_4_O_2_[M + Na]^+^ 449.0407, found 449.0409.

***2-[5-(2-Chloropyridin-4-yl)-1,3-dimethyl-1H-pyrazol-4-yl]-6-bromo-8-methyl-4H-3,1-benzoxazin-4-one (8w)***-yellow solid, yield 30%, m.p. 184.6–184.9 °C, ^1^H NMR (400 MHz, Chloroform-*d*) δ 8.57 (dd, J = 4.9, 2.0 Hz, 1H), 8.04 (dd, J = 2.3, 0.8 Hz, 1H), 7.74 (dd, J = 7.5, 2.0 Hz, 1H), 7.61 (dd, J = 2.3, 1.0 Hz, 1H), 7.44 (dd, J = 7.6, 4.8 Hz, 1H), 3.68 (s, 3H), 2.68 (s, 3H), 2.04 (d, J = 0.8 Hz, 3H). ^13^C NMR (101 MHz, Chloroform-*d*) δ 158.48, 153.34, 153.02, 150.14, 148.08, 144.41, 141.85, 140.31, 137.61, 136.99, 132.26, 125.28, 125.18, 117.38, 111.39, 37.15, 16.92, 16.55, 14.81. LCMS (ESI): calculated for C_19_H_14_^79^BrClN_4_O_2_[M + Na]^+^ 466.9881, found 466.9893, calculated for C_19_H_14_^81^BrClN_4_O_2_[M + Na]^+^ 468.9861, found 468.9874.

***2-[5-(2-Chloropyridin-4-yl)-1,3-dimethyl-1H-pyrazol-4-yl]-8-fluoro-4H-3,1-benzoxazin-4-one (8x)***-white solid, yield 41%, m.p. 210.1–210.7 °C, ^1^H NMR (400 MHz, Chloroform-*d*) δ 8.58 (dd, J = 4.9, 1.9 Hz, 1H), 7.86 (dt, J = 7.9, 1.2 Hz, 1H), 7.79 (dd, J = 7.6, 2.0 Hz, 1H), 7.46–7.38 (m, 2H), 7.31 (td, J = 8.0, 4.7 Hz, 1H), 3.70 (s, 3H), 2.70 (s, 3H). ^13^C NMR (101 MHz, Chloroform-*d*) δ 157.97, 156.28, 154.47, 150.94, 150.55, 141.52, 141.11, 136.63, 136.51, 127.50, 126.12, 123.88, 122.38, 122.22, 118.31, 111.11, 37.14, 14.74. LCMS (ESI): calculated for C_18_H_12_ClFN_4_O_2_[M + Na]^+^ 393.0526, found 393.0540.

***2-[5-(2-Chloropyridin-5-yl)-1,3-dimethyl-1H-pyrazol-4-yl]-8-methyl-4H-3,1-benzoxazin-4-one (8y)***-white solid, yield 58%, m.p. 209.3–209.9 °C, ^1^H NMR (400 MHz, Chloroform-*d*) δ 8.47–8.43 (m, 1H), 7.96–7.90 (m, 1H), 7.75 (dd, J = 8.2, 2.4 Hz, 1H), 7.54–7.48 (m, 2H), 7.25 (t, J = 7.6 Hz, 1H), 3.71 (s, 3H), 2.65 (s, 3H), 2.17 (s, 3H). ^13^C NMR (101 MHz, Chloroform-*d*) δ 159.51, 153.02, 152.45, 150.42, 150.17, 145.50, 141.23, 140.37, 137.20, 135.46, 127.10, 126.01, 125.47, 124.22, 116.49, 111.67, 37.24, 16.97, 14.87. LCMS (ESI): calculated for C_19_H_15_ClN_4_O_2_[M + Na]^+^ 389.0776, found 389.0789.

***2-[5-(2-chloropyridin-5-yl)-1,3-dimethyl-1H-pyrazol-4-yl]-6-chloro-8-methyl-4H-3,1-benzoxazin-4-one (8z)***-yellow solid, yield 55%, m.p. 192.2–192.7 °C, ^1^H NMR (400 MHz, Chloroform-*d*) δ 8.43 (dd, J = 2.4, 0.7 Hz, 1H), 7.87 (dd, J = 2.5, 0.7 Hz, 1H), 7.74 (dd, J = 8.2, 2.4 Hz, 1H), 7.51 (dd, J = 8.2, 0.8 Hz, 1H), 7.47 (dd, J = 2.5, 1.0 Hz, 1H), 3.71 (s, 3H), 2.64 (s, 3H), 2.14 (s, 3H). ^13^C NMR (101 MHz, Chloroform-*d*) δ 158.44, 153.33, 152.59, 150.42, 150.34, 144.22, 141.44, 140.28, 137.69, 137.14, 132.47, 125.38, 125.21, 124.28, 117.47, 111.39, 37.30, 16.90, 14.95. LCMS (ESI): calculated for C_19_H_14_C_l2_N_4_O_2_[M + Na]^+^ 423.0387, found 423.0406.

### 3.3. Fungicidal Activity Assay

The fungal strains used in this study were preserved in the Fungicide Laboratory of the National Engineering Research Center for Pesticides, Nankai University, which was also responsible for conducting the bioassays.

For the bioassay of cucumber anthracnose: (1) Preparation of PDA medium: Briefly, 200 g of fresh peeled potatoes were cut into small pieces, then boiled in 1000 mL distilled water for 30 min. The resulting liquid was filtered through two layers of gauze. Afterwards, 20 g of glucose and 20 g of agar powder were supplemented, and the total volume was made up to 1 L with distilled water. The mixture was heated slowly until agar was fully dissolved, then autoclaved at 121 °C for 20 min and kept at room temperature for subsequent use. (2) For sample preparation, 30 mg of each target compound was first dissolved in DMSO and then diluted with sterile 0.1% Tween-80 aqueous solution to prepare a 500 mg/L stock solution. Mycelial growth inhibition assay was adopted in this study. In a sterile Petri dish, 1 mL of the above stock solution was added and then mixed thoroughly with 9 mL melted PDA medium. The plate was gently swirled and left to cool down for solidification. A 5 mm mycelial plug was excised from the edge of actively growing colonies with a sterile cork borer and placed onto the drug-containing medium surface. All plates were incubated at 25 °C under dark conditions, and the colony diameter was recorded after 3 days of cultivation. Each treatment was conducted with four biological replicates. Meanwhile, a blank control group was set correspondingly, using 0.1% sterile Tween-80 solution containing the same amount of DMSO without any tested compound.

For the bioassay of maize rust: (1) Inoculation and cultivation: Fresh spores of maize rust were suspended in sterile water to prepare a spore suspension at a concentration of 1 × 105 spores/mL. At 24 h after spray application of the test compound, seedlings were uniformly inoculated with the spore suspension under gentle agitation, then allowed to air-dry naturally. All plants were incubated in a constant-temperature chamber at 25 °C for 7–8 d. (2) Each test compound (30 mg) was dissolved in DMSO to afford a 30,000 ppm stock solution. On the basis of the preliminary bioactivity, a series of working concentrations were prepared by further dilution with sterile aqueous 0.1% Tween-80 solution. The resultant solutions were evenly sprayed onto maize seedlings at the two-leaf stage with consistent leaf size until complete leaf wetness was achieved. The treated seedlings were air-dried naturally, with two seedlings grown per paper cup. A blank control was established using only solvent and surfactant without active compounds.

All bioassay data were collated and summarized in Table 3.

### 3.4. Molecular Docking Analysis

The binding site was predicted using AutoDock 4.2, and a total of 26 compounds were virtually screened via Vina. Compound **8j** exhibited the optimal docking score of −11.8 kcal/mol, which was considerably higher than that of fluxapyroxad (−8.9 kcal/mol). Both compounds anchored within the conserved hydrophobic pocket of SDH through π–π stacking and hydrophobic interactions with Phe153, Trp166, Val159, Val162, and Leu198. The prominent affinity of **8j** was mainly derived from the polar contact between its carbonyl group and Lys163, as well as lipophilic interactions provided by the terminal chlorine atom. Although fluxapyroxad penetrated deeper into the binding cavity and formed extra hydrophobic interactions with Leu195 and Met87, it failed to generate a polar interaction with Lys163. Additionally, the electron-withdrawing fluorine atom further weakened its binding capability, thereby showing lower complex stability than **8j**. Consequently, the polar interaction with Lys163 was essential for maintaining high binding affinity, and supplementary hydrophobic contacts could not compensate for the absence of this key polar anchoring effect.

## 4. Conclusions

In conclusion, a series of pyridylpyrazole–benzoxazinone derivatives (**8a**–**8z**) were efficiently synthesized via a metal-free strategy in this study. The optimal reaction conditions were determined by orthogonal experiments as follows: 3.0 equivalents of pyridine, water as the solvent, BF_4_^−^ as the counterion, and reaction at 25 °C for 3 h. Most target compounds displayed favorable fungicidal activity against maize rust and cucumber anthracnose. Among them, compound 8p showed excellent inhibitory activity against maize rust, with an inhibition rate of 97.0% at 200 mg/L and 92.0% at 50 mg/L. Compound **8q** exhibited the highest activity against cucumber anthracnose, with an inhibition rate of 90.5% at 50 mg/L. Although the present study focused on fungicidal activity, preliminary toxicity assessments (e.g., cytotoxicity or ecotoxicity) of the lead compounds will be conducted in future work to further evaluate their potential as agrochemical candidates. The fungicidal activity was controlled by the electronic effects and steric hindrance of the substituents on the benzoxazinone skeleton and the pyridine side chain. This study validates the feasibility of the metal-free strategy and provides an important structural basis for the development of sustainable, low-risk, and broad-spectrum agricultural fungicides.

## Figures and Tables

**Table 1:** Effects of solvent and diazonium salt counterion on the reaction.

Entry	Solvent	Counteranion	Pyridine (Equiv.)	Time (h)	Temp. (°C)	Yield of Ortho-Product (%)
**1**	Deionized water	BF_4_^−^	2.0	1	25	56.4
**2**	CH_3_CN	BF_4_^−^	2.0	1	25	49.6
**3**	EtOH	BF_4_^−^	2.0	1	25	11.3
**4**	DMSO	BF_4_^−^	2.0	1	25	53.5
**5**	DMF	BF_4_^−^	2.0	1	25	49.7
**6**	DCM	BF_4_^−^	2.0	1	25	6.5
**7**	EA	BF_4_^−^	2.0	1	25	9.1
**8**	Toluene	BF_4_^−^	2.0	1	25	0 (side reaction)
**9**	Deionized water	H_2_PO_4_^−^	2.0	1	25	11.8
**10**	Deionized water	Cl^−^	2.0	1	25	22.5
**11**	Deionized water	HSO_4_^−^	2.0	1	25	37.4

**Table 2:** Orthogonal study of reaction conditions.

Entry	Pyridine	Time	Temp	Yield (%)
**1**	1.0 eq	1	0	27.3
**2**	1.0 eq	2	25	39.2
**3**	1.0 eq	3	40	41.5
**4**	2.0 eq	1	25	56.4
**5**	2.0 eq	2	40	60.1
**6**	2.0 eq	3	0	43.7
**7**	3.0 eq	1	40	57.5
**8**	3.0 eq	2	0	45.9
**9**	3.0 eq	3	25	63.7
K1 *	108	141.2	116.9	
**K2**	160.2	145.2	159.3	
**K3**	167.1	148.9	159.1	
**k1 = K1/3**	36.0	47.1	39.0	
**k2 = K2/3**	53.4	48.4	53.1	
**k3 = K3/3**	55.7	49.6	53.0	
**Range**	19.7	2.6	14.1	
**Factor influence**	Pyridine amount > Reaction temperature > Reaction time.			
**Optimal conditions**	A_3_ (3.0 equiv. of pyridine), B_3_ (3 h), C_2_ (25 °C).			

* K1, K2, K3: the sum of the yields at the first, second and third levels representing factors A, B, C respectively.

**Table 3:** Fungicidal activity of target compounds against maize rust (in vivo) and cucumber anthracnose (in vitro).

	Concentration (mg/L)						
Compound	*Puccinia sorghi*		*Colletotrichum orbiculare*	Compound	*Puccinia sorghi*		*Colletotrichum orbiculare*
	200	50	50		200	50	50
**8** **a**	87.0	84.4	53.85	**8n**	70.0	37.0	47.6
**8** **b**	64.0	46.0	85.7	**8o**	41.0	34.0	61.9
**8** **c**	72.0	57.0	71.4	**8p**	97.0	92.0	54.5
**8** **d**	69.0	43.0	81.0	**8q**	58.0	43.0	90.5
**8e**	70.0	61.0	76.2	**8r**	74.0	62.0	85.7
**8** **f**	54.0	44.0	71.4	**8s**	57.0	37.0	61.9
**8** **g**	77.0	66.0	71.4	**8t**	66.0	47.0	42.9
**8** **h**	52.0	45.0	85.7	**8u**	69.0	60.0	71.4
**8** **i**	70.0	65.0	76.2	**8v**	87.6	25.0	46.15
**8** **j**	100	62.0	38.46	**8w**	69.0	33.0	85.7
**8k**	75.0	52.0	61.9	**8x**	59.0	48.0	71.4
**8l**	90.0	82.0	36.4	**8y**	85.0	38.3	46.15
**8m**	74.5	38.6	54.0	**8z**	72.0	63.0	85.7
**Azoxystrobin**	100	100	/	**Prochloraz**	/	/	100

**Table 4:** EC_50_ values of azoxystrobin and target compounds against corn rust.

Compound	y = a + bx	R	EC_50_ (mg/L)
**8a**	y = 2.915 + 1.521 lgC	0.9952	23.49
**8j**	y = 2.986 + 1.492 lgC	0.9937	22.38
**8l**	y = 3.552 + 1.181 lgC	0.9508	16.83
**8p**	y = 3.041 + 1.467 lgC	0.9869	21.65

## Data Availability

Samples of the compounds are not available from the authors.

## Supplementary Materials

The following supporting information can be downloaded at: https://www.mdpi.com/article/10.3390/ijms27135903/s1.
