# Supplementary material for: Synthesis and Fungicidal Evaluation of Pyridylpyrazole Derivatives Bearing a Benzoxazinone Scaffold via a Metal-Free Approach
Source: Int J Mol Sci. 2026 Jun 30;27(13):5903. doi: 10.3390/ijms27135903 (PMC13361420; doi:10.3390/ijms27135903)
Supplement: Supplementary file 1 [file ijms-27-05903-s001.zip › ijms-4374432-supplementary.pdf]

Synthesis and Fungicidal Evaluation of Pyridylpyrazole Derivatives Bearing a Benzoxazinone Scaffold via a Metal-Free Approach

Bin-Ran Zeng, Jing-Ya Liu, Rui-Han Hu and Xiao-Hua Du *

Catalytic Hydrogenation Research Center, Zhejiang Key Laboratory of Green Pesticides and Cleaner Production Technology, Zhejiang Green Pesticide Collaborative Innovation Center, Zhejiang University of Technology, Hangzhou 310014, P. R. China

*Corresponding author.
E-mail addresses: duxiaohua@zjut.edu.cn

Supporting Information

Contents:

1. ^{1}H NMR of target compounds **3a,3b**;

2. ^{1}H NMR and ^{13}C NMR of target compounds **5a–5l,5o**;

3. ^{1}H NMR and of target compounds **5p–5s**;

4. LCMS Chromatogram of Compound **5m;**

5. ^{1}H NMR and ^{13}C NMR of target compounds **8a–8z**;

6. Molecular docking binding energies of target compounds (**8a–8z**).

7. Inhibitory activities of potent compounds against maize rust across different concentrations;

8. Synthetic Steps and Respective ^{1}H NMR of Diazonium Salts;

9. HPLC-MS of Compounds with Potential Biological Activity.

1.¹H NMR of target compounds 3a,3b.

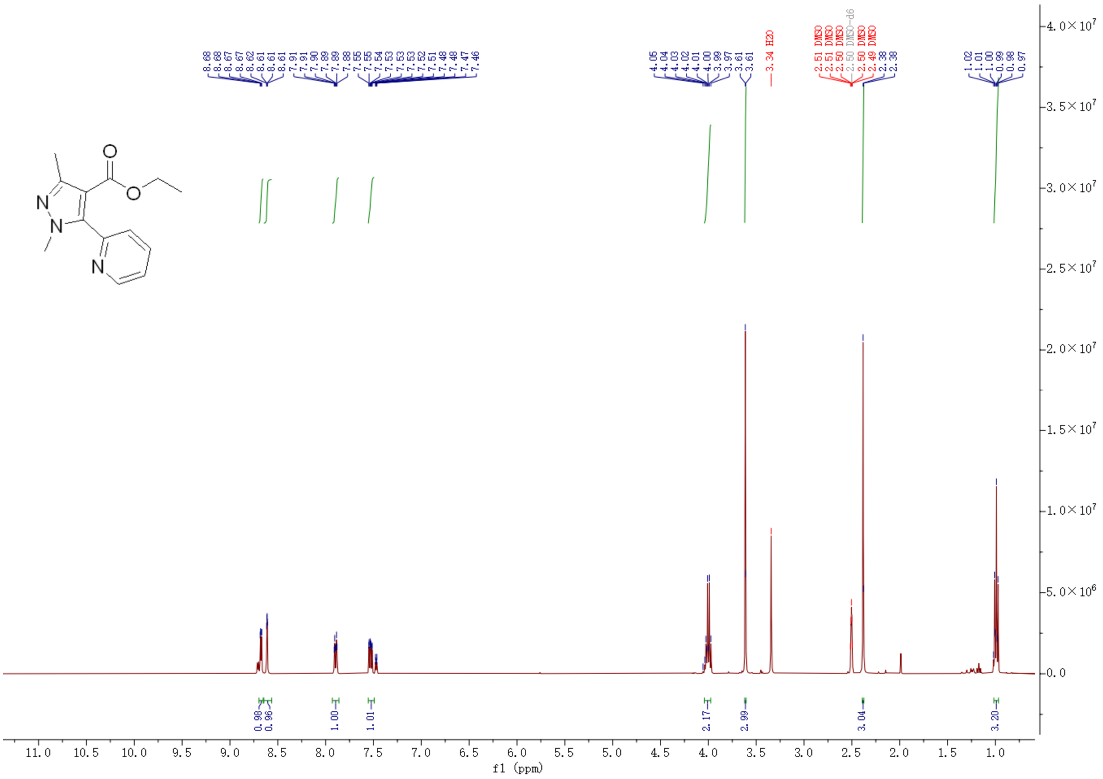

Figure S1. The ¹H NMR of **3a** (DMSO-d*6*).

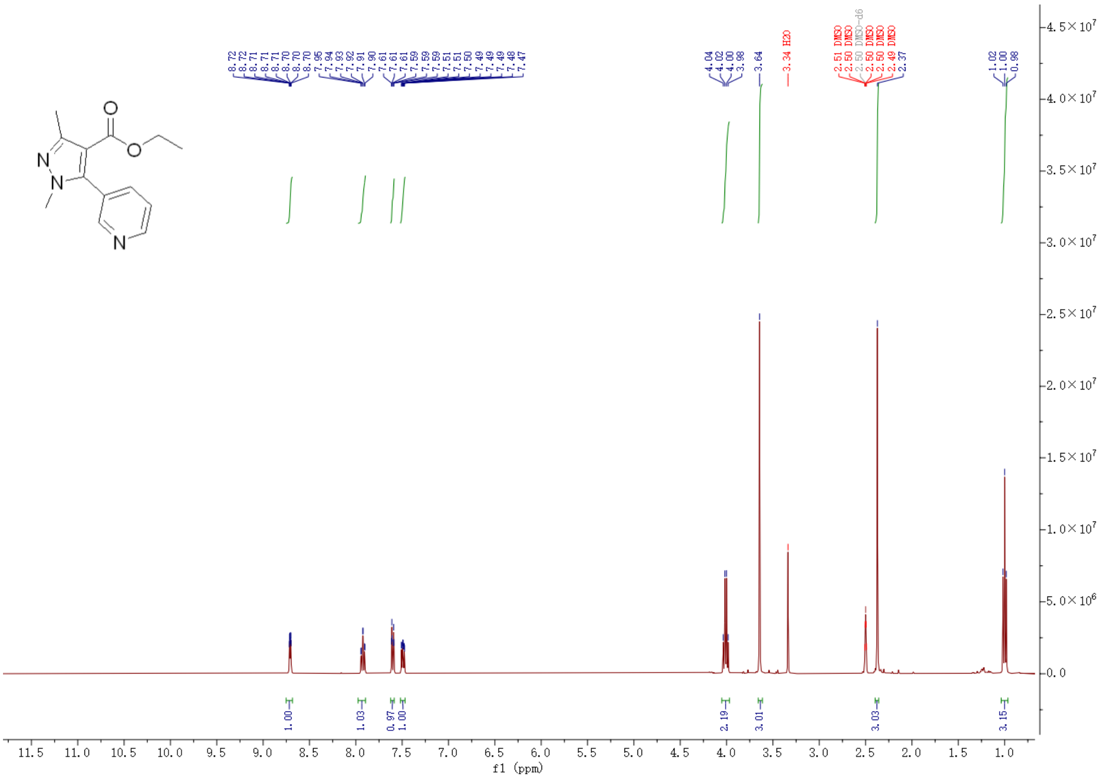

Figure S2. The ¹H NMR of **3b** (DMSO-d*6*).

2.¹H NMR and ¹³C NMR of target compounds 5a-5l.

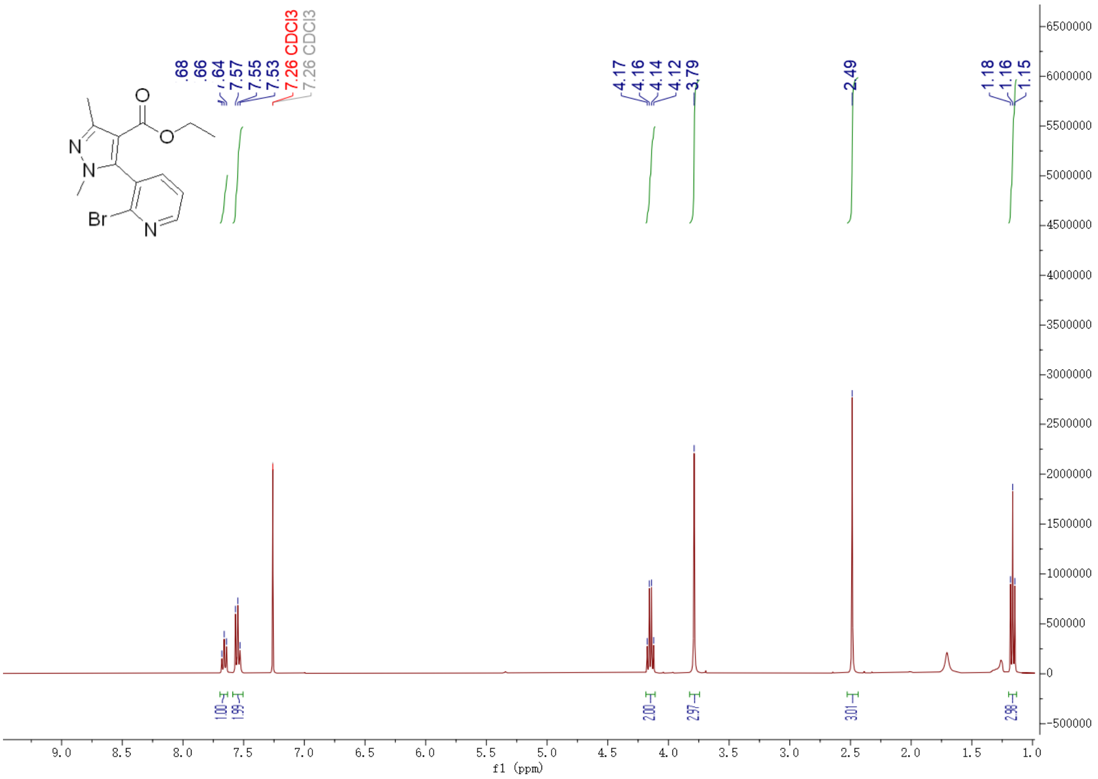

Figure S3. The ¹H NMR of **5a** (Chloroform-*d*).

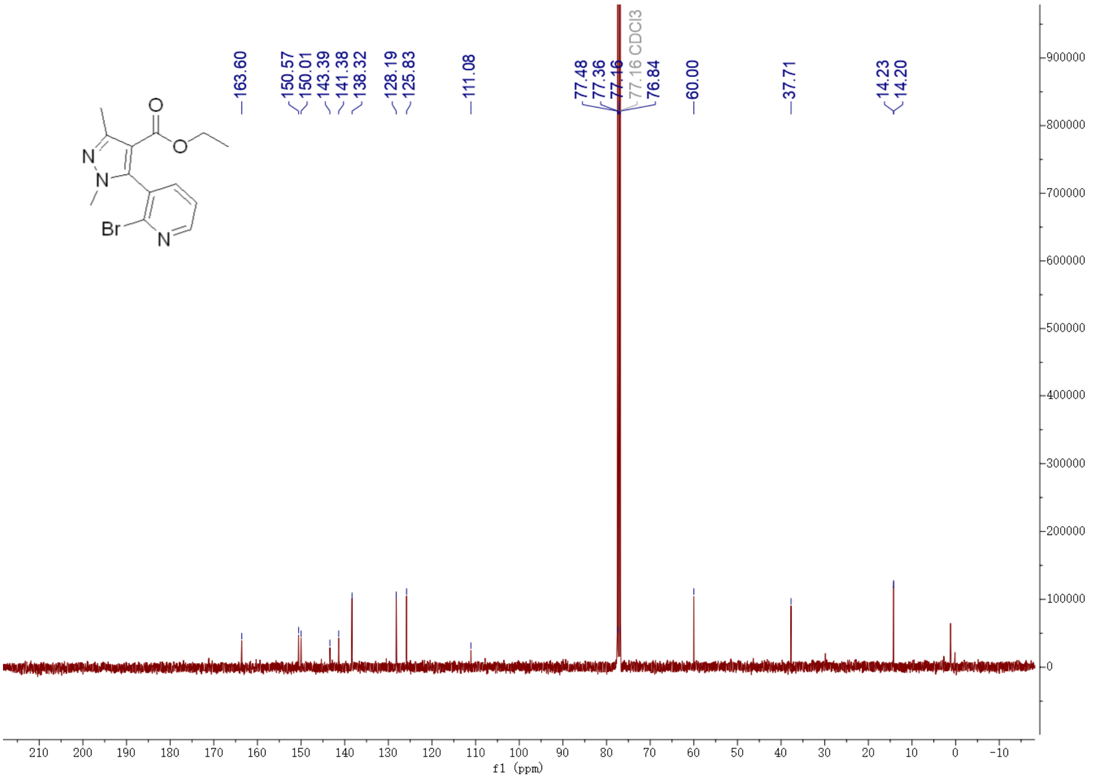

Figure S4. The ¹³C NMR of **5a** (Chloroform-*d*).

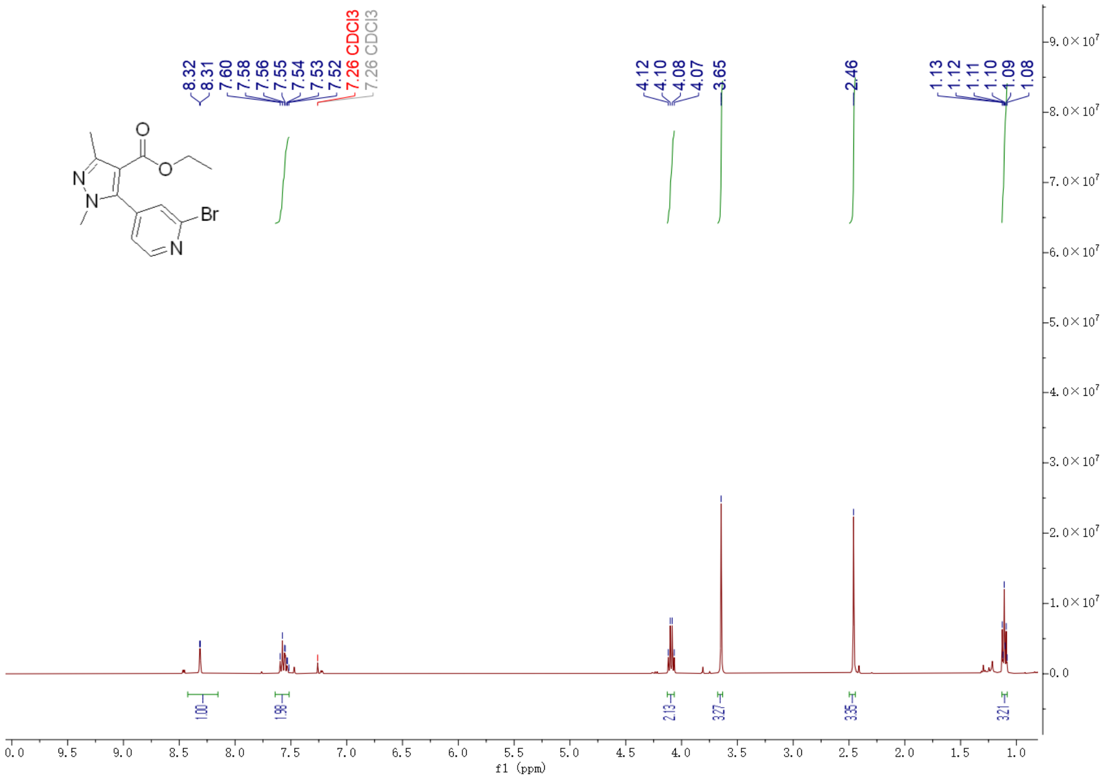

Figure S5. The ^{1}H NMR of **5b** (Chloroform-*d*).

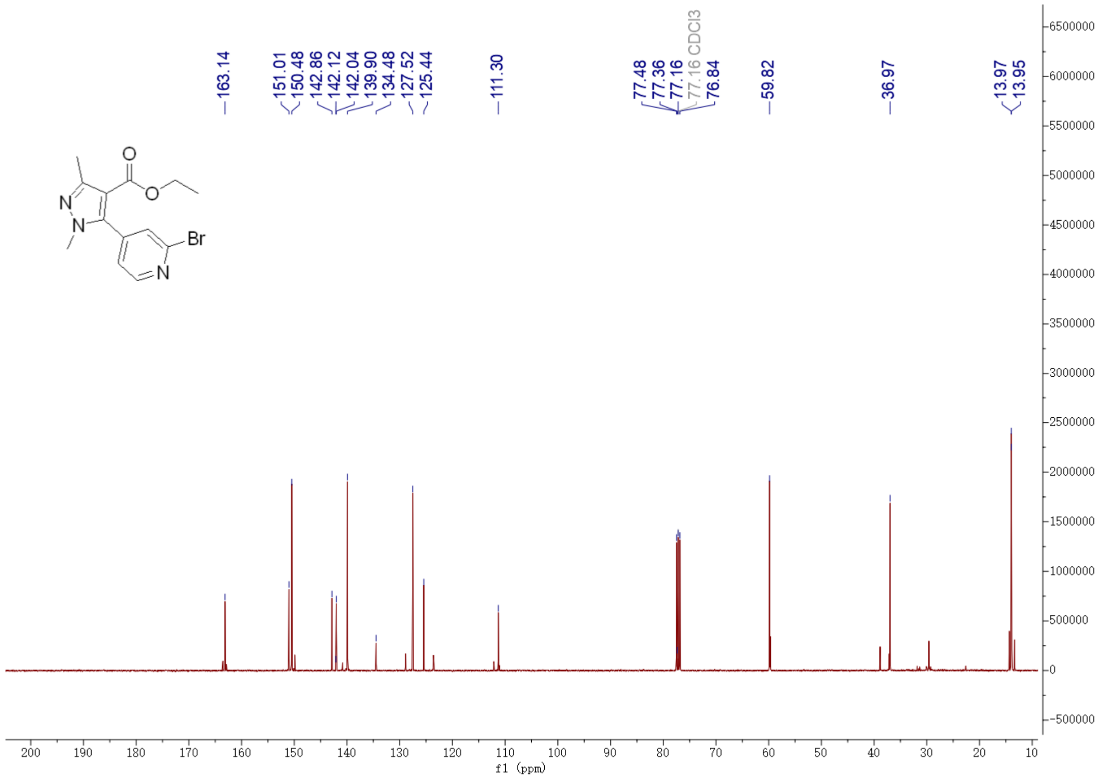

Figure S6. The ^{13}C NMR of **5b** (Chloroform-*d*).

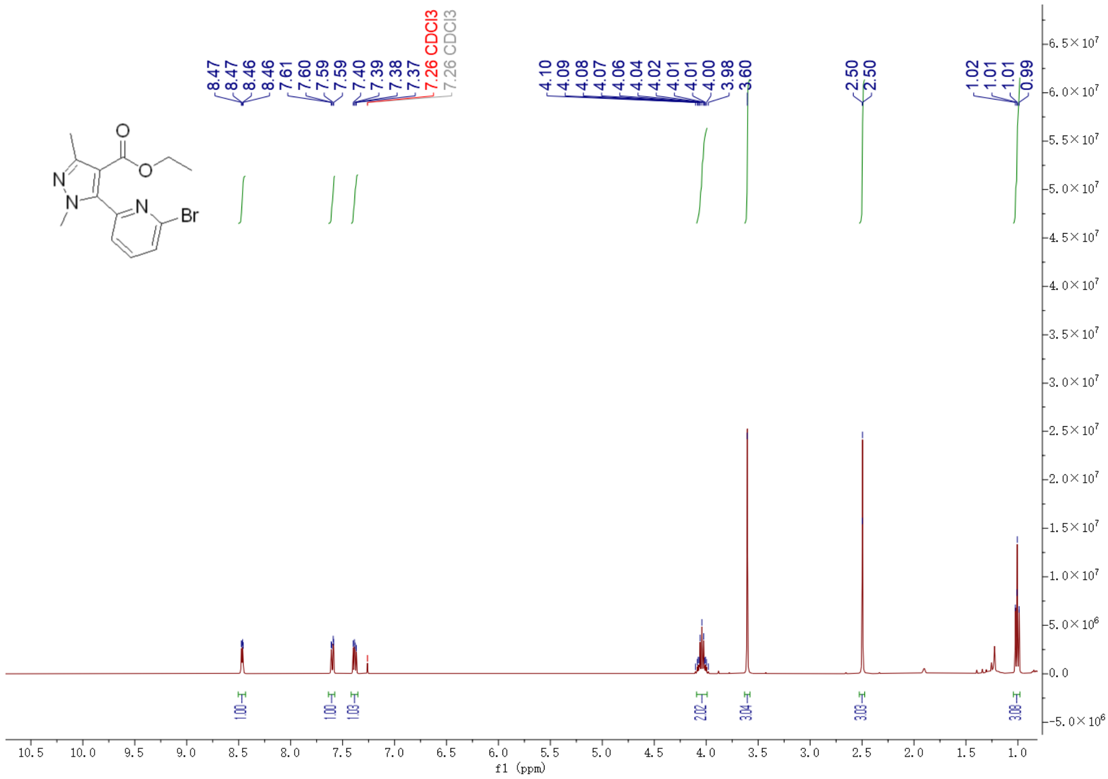

Figure S7. The ^{1}H NMR of **5c** (Chloroform-*d*).

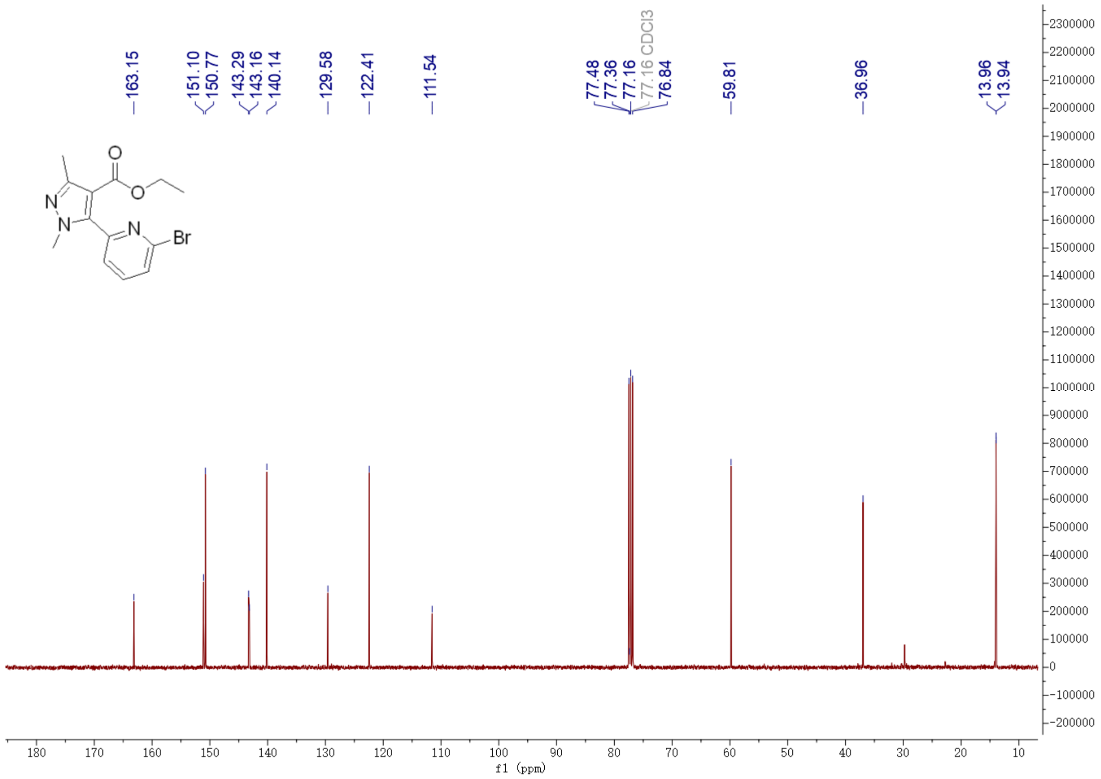

Figure S8. The ^{13}C NMR of **5c** (Chloroform-*d*).

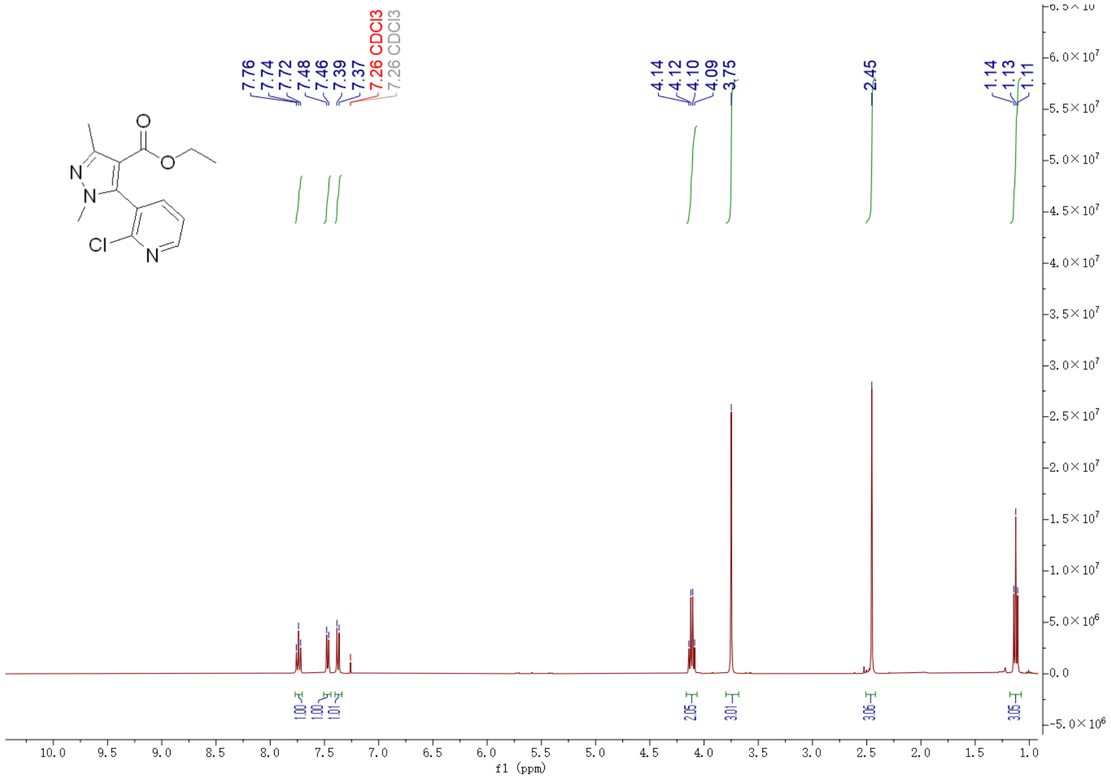

Figure S9. The ^{1}H NMR of **5d** (Chloroform-*d*).

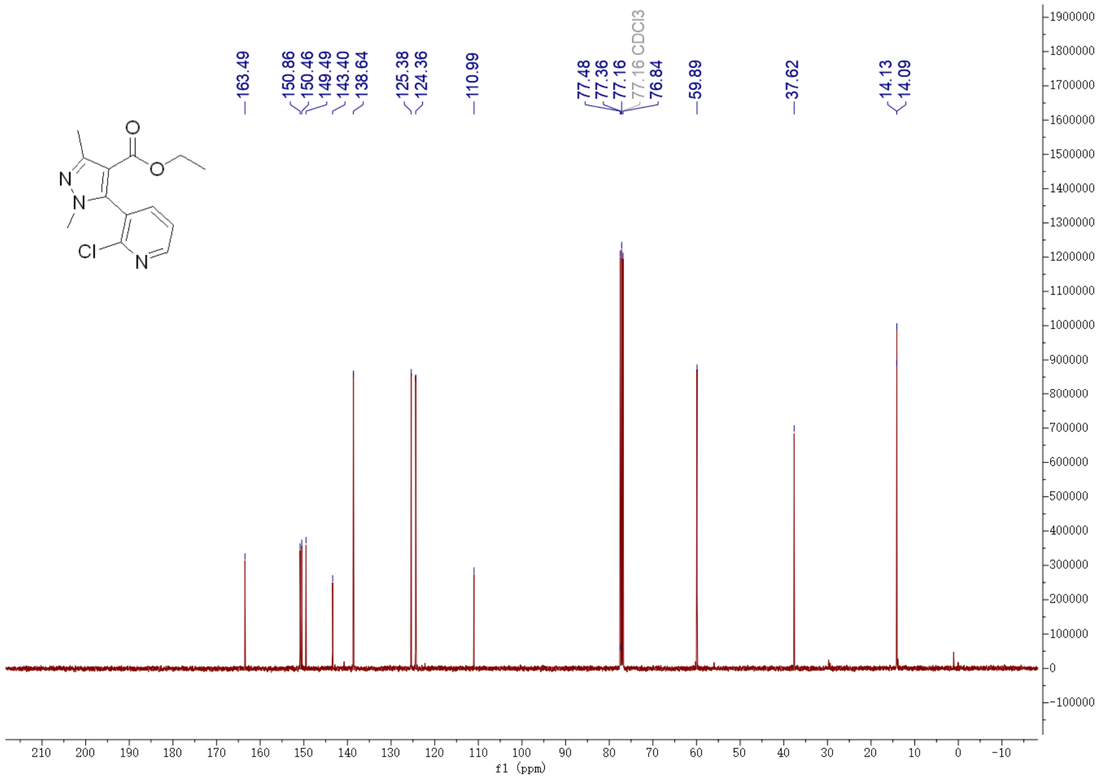

Figure S10. The ^{13}C NMR of **5d** (Chloroform-*d*).

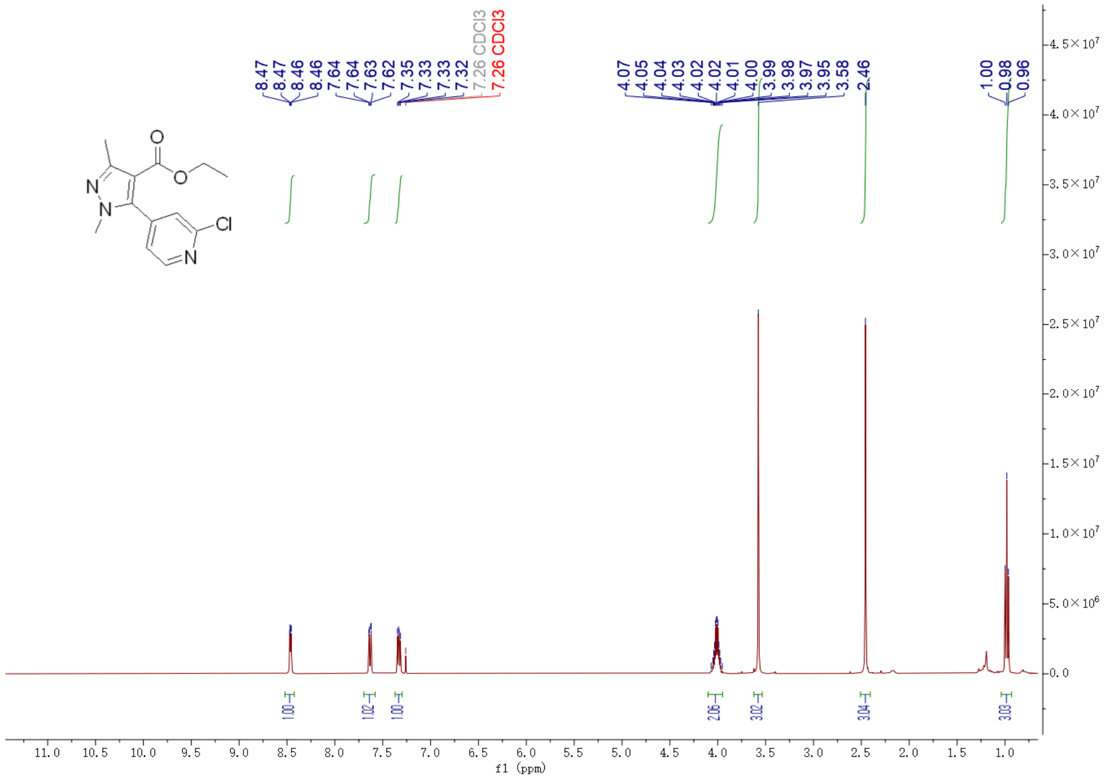

Figure S11. The ^{1}H NMR of **5e** (Chloroform-*d*).

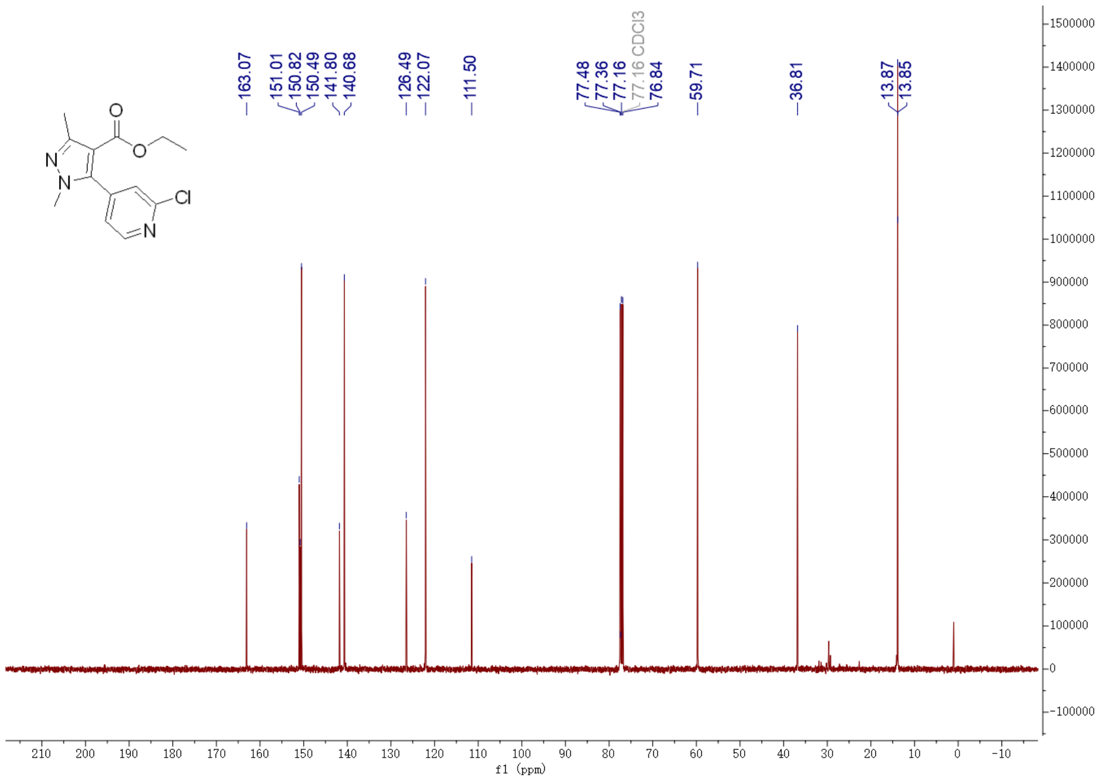

Figure S12. The ^{13}C NMR of **5e** (Chloroform-*d*).

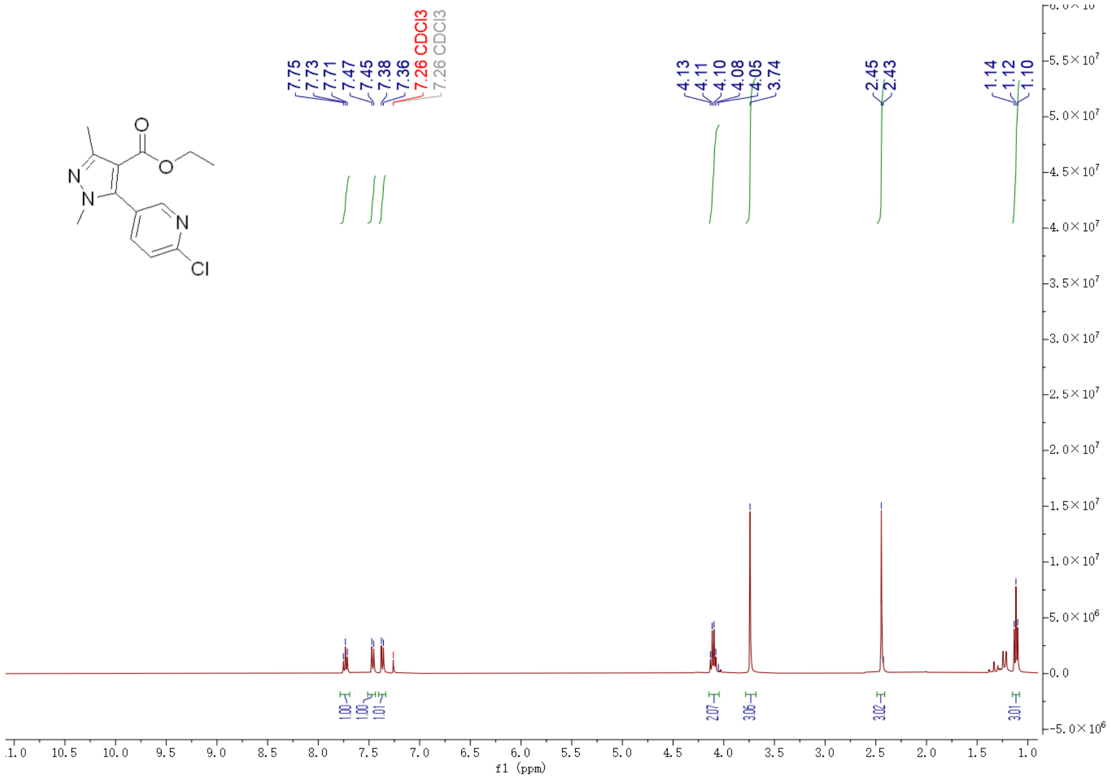

Figure S13. The ^{1}H NMR of **5f** (Chloroform-*d*).

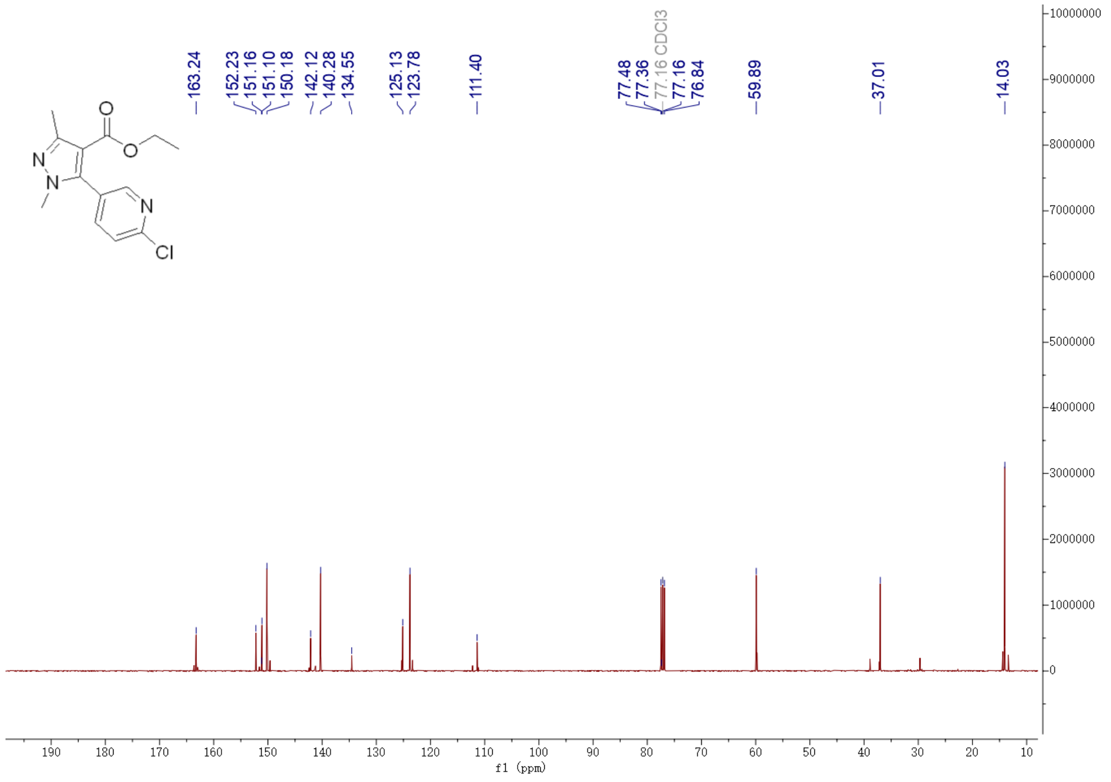

Figure S14. The ^{13}C NMR of **5f** (Chloroform-*d*).

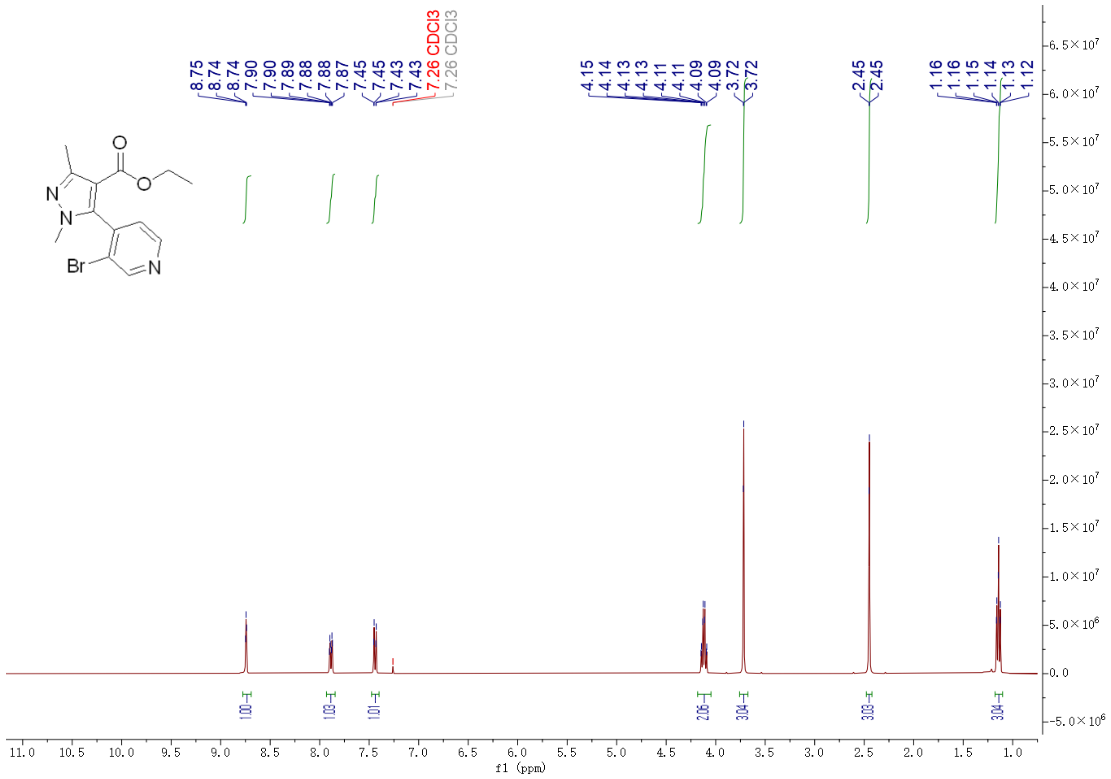

Figure S15. The ^{1}H NMR of **5g** (Chloroform-*d*).

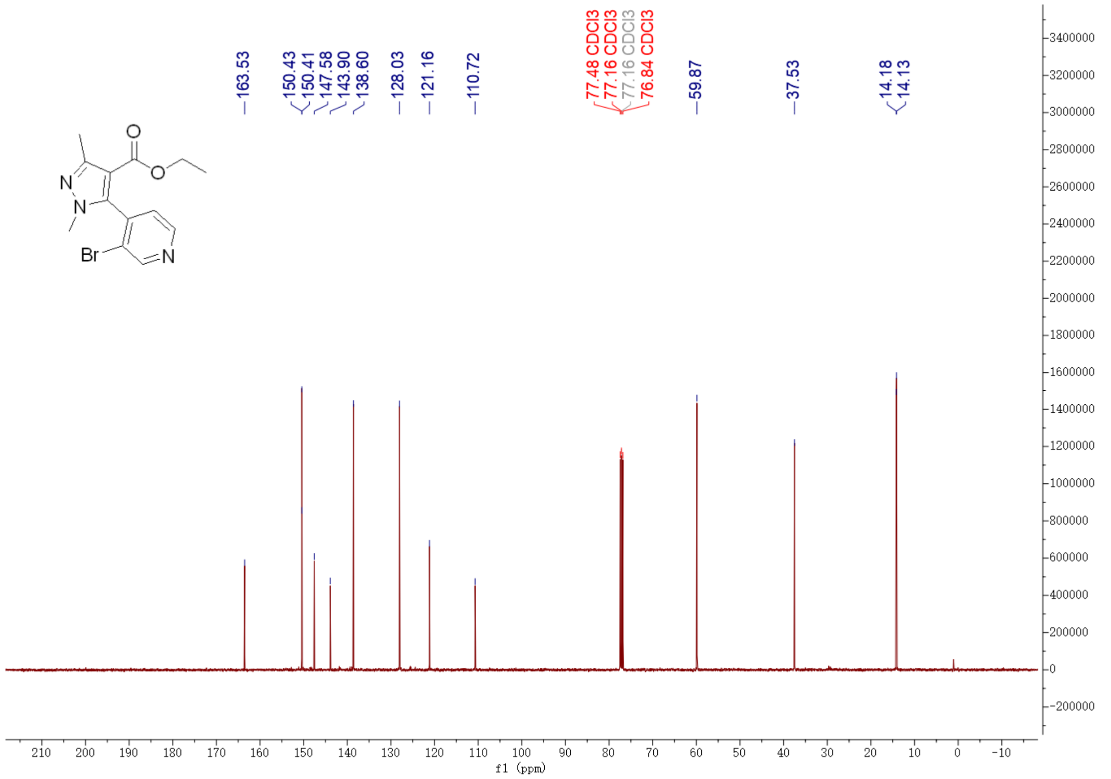

Figure S16. The ^{13}C NMR of **5g** (Chloroform-*d*).

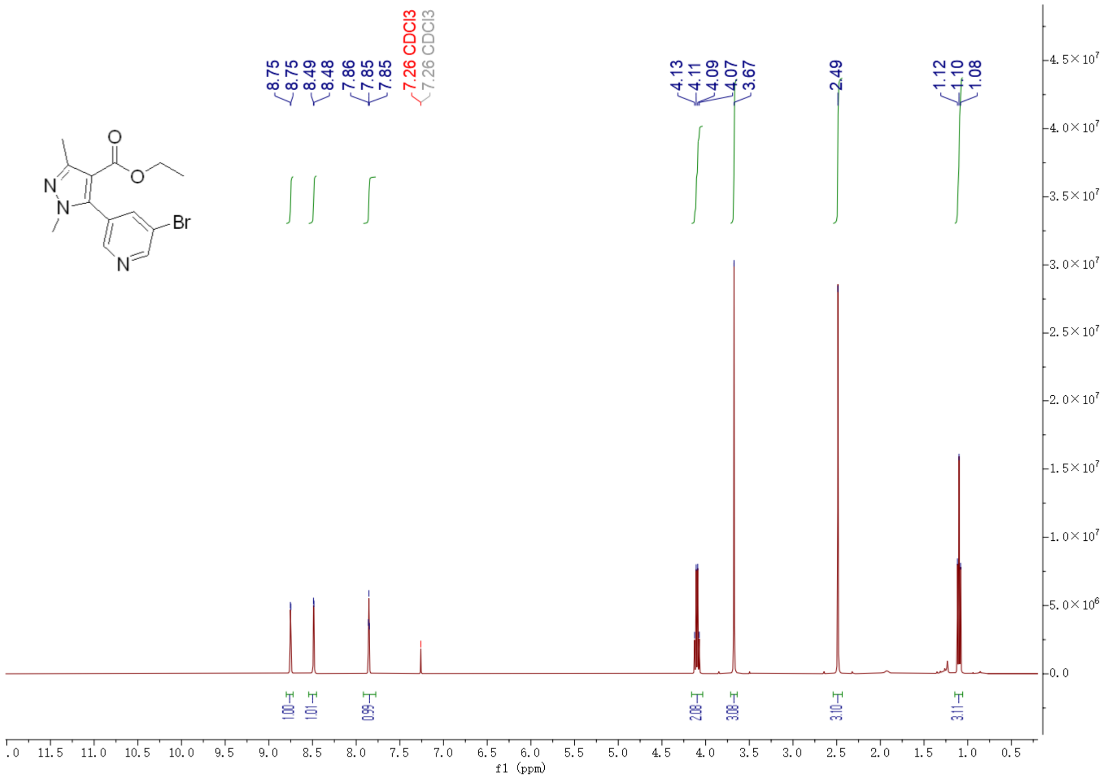

Figure S17. The ^{1}H NMR of **5h** (Chloroform-*d*).

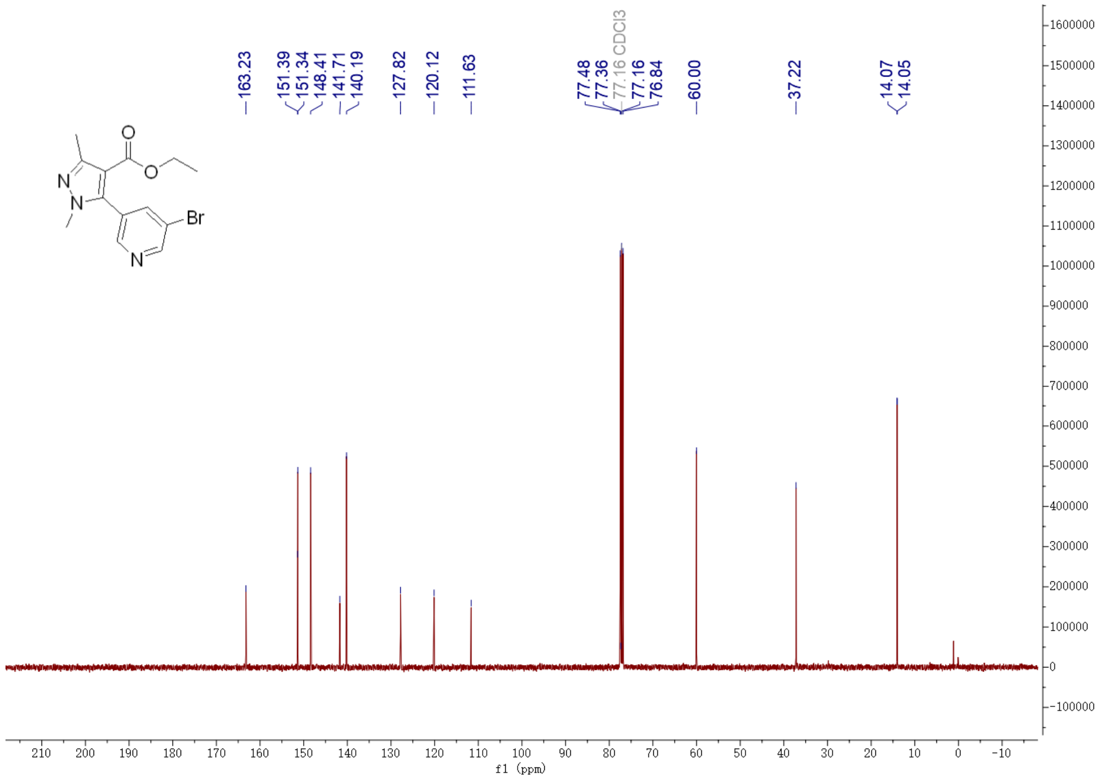

Figure S18. The ^{13}C NMR of **5h** (Chloroform-*d*).

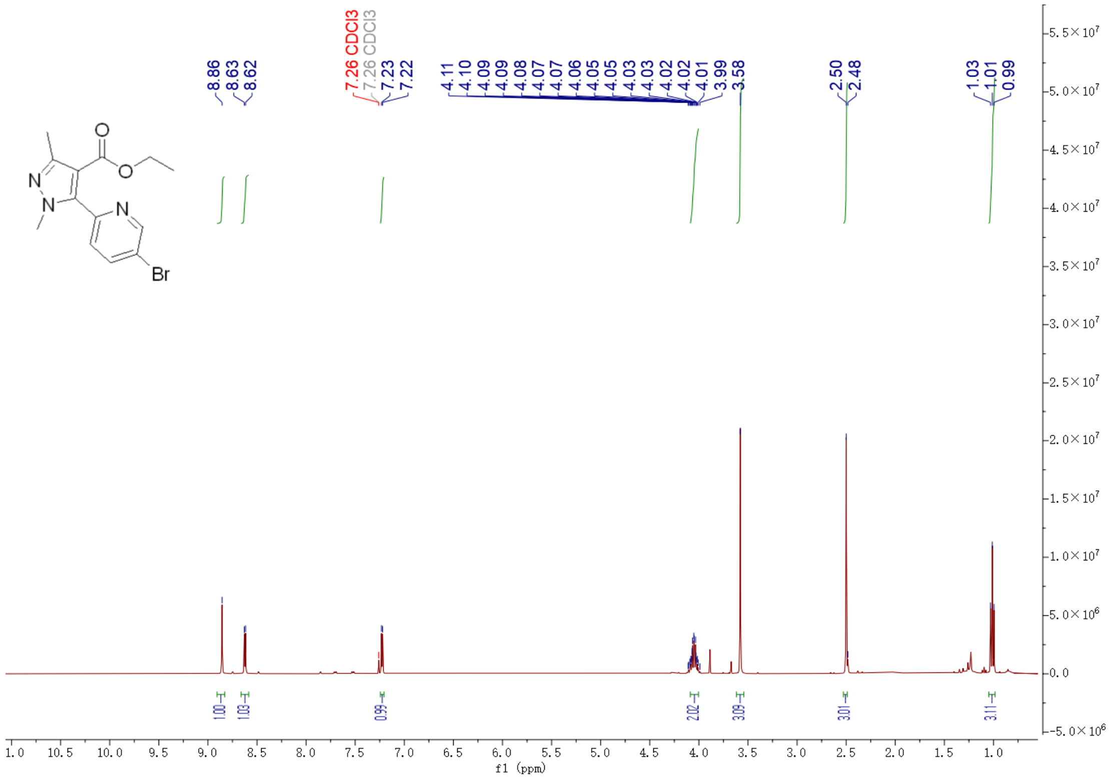

Figure S19. The ^{1}H NMR of **5i** (Chloroform-*d*).

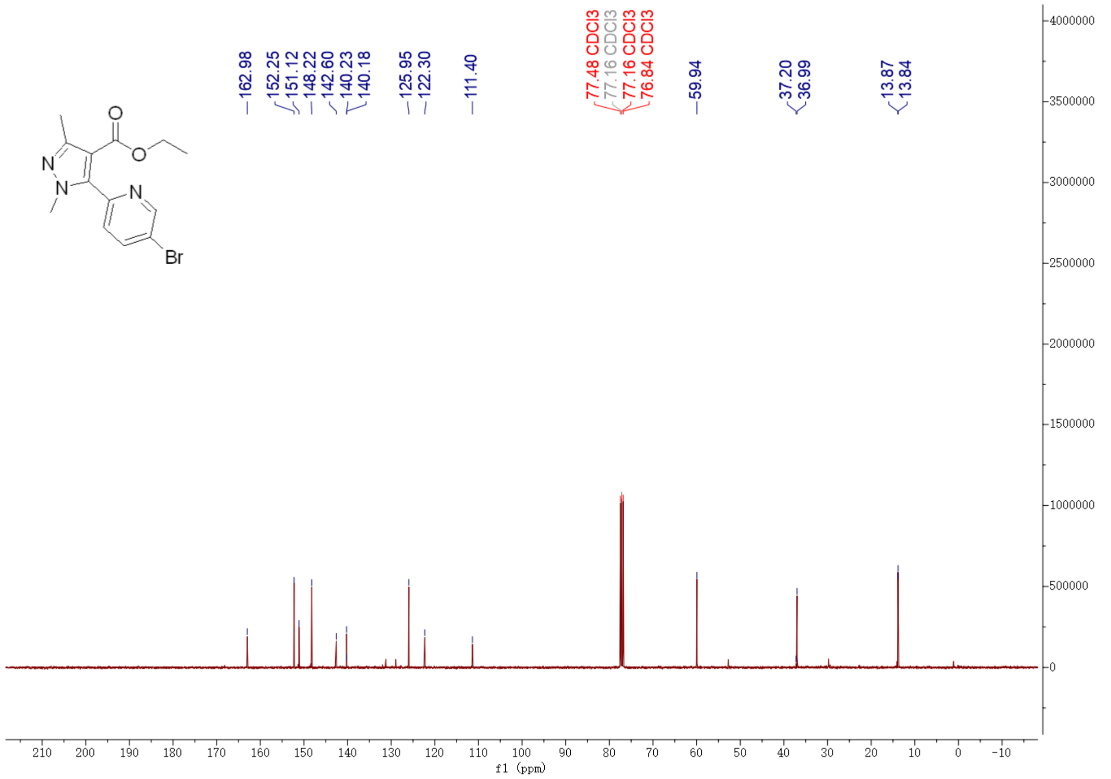

Figure S20. The ^{13}C NMR of **5i** (Chloroform-*d*).

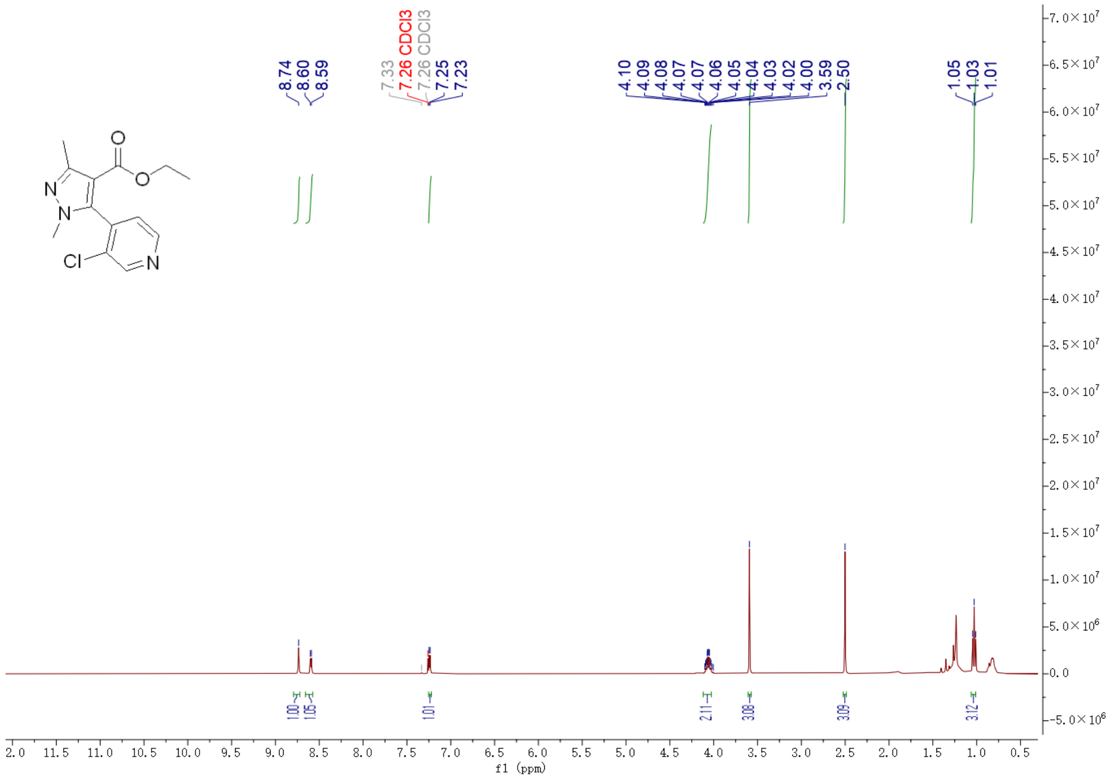

Figure S21. The ^{1}H NMR of **5j** (Chloroform-*d*).

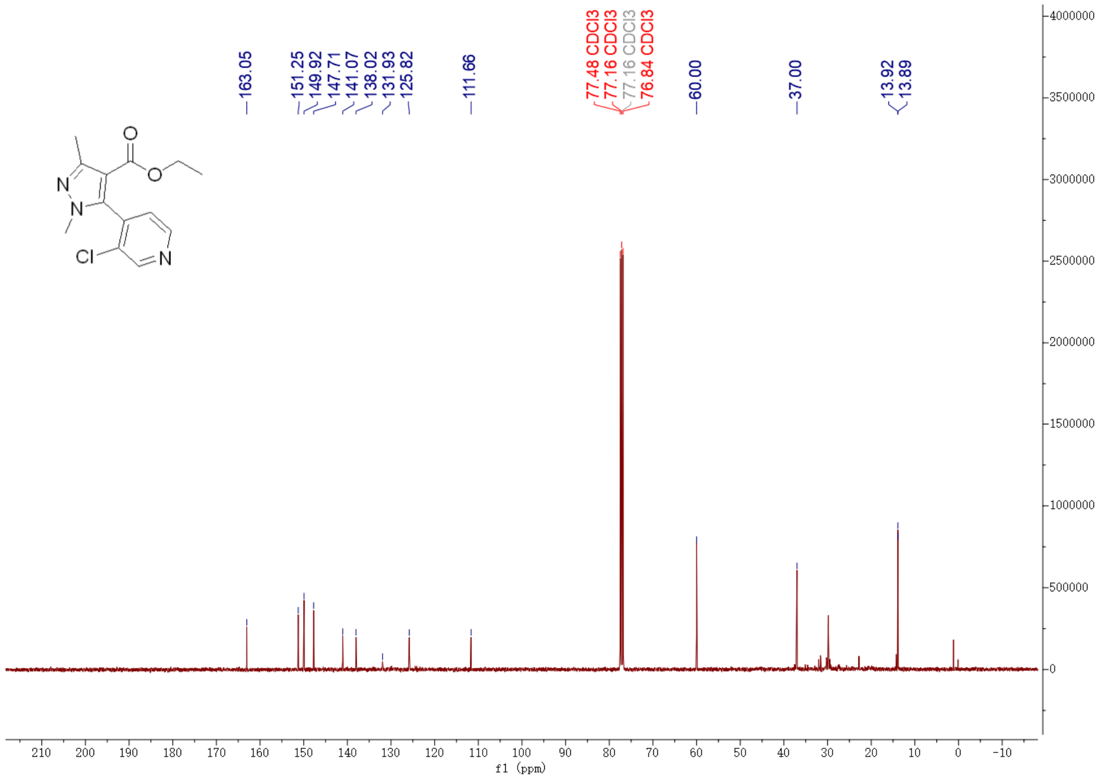

Figure S22. The ^{1}H NMR of **5j** (Chloroform-*d*).

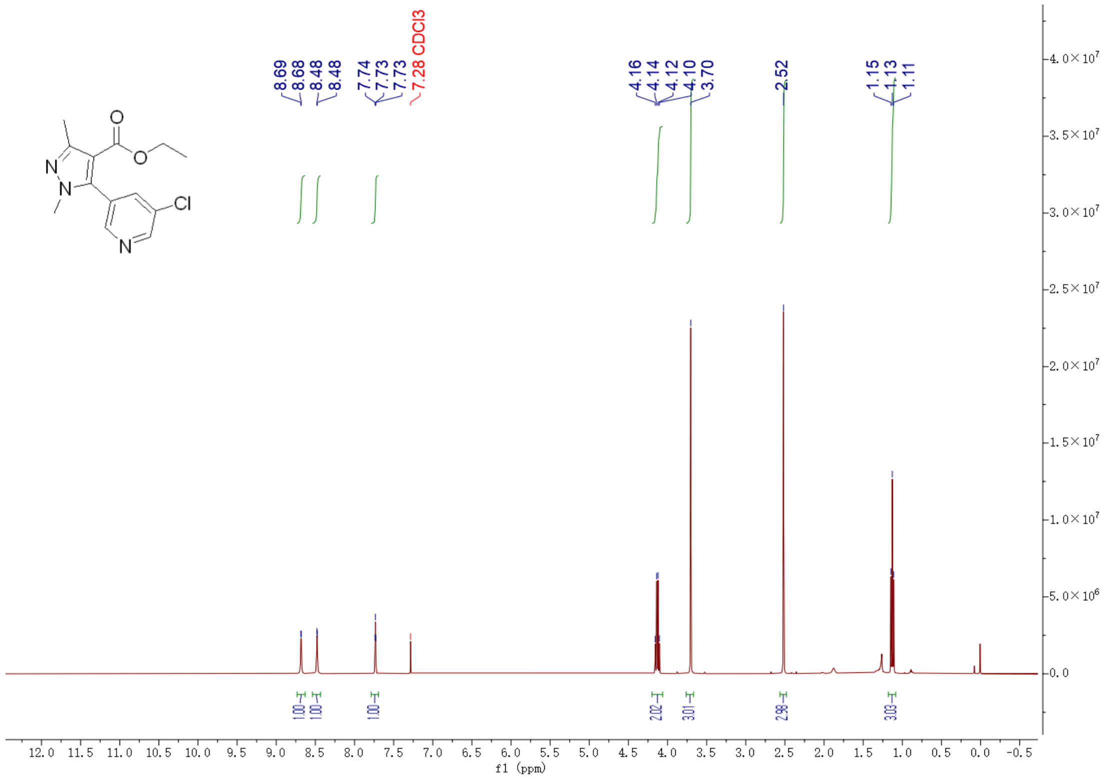

Figure S23. The ^{1}H NMR of **5k** (Chloroform-*d*).

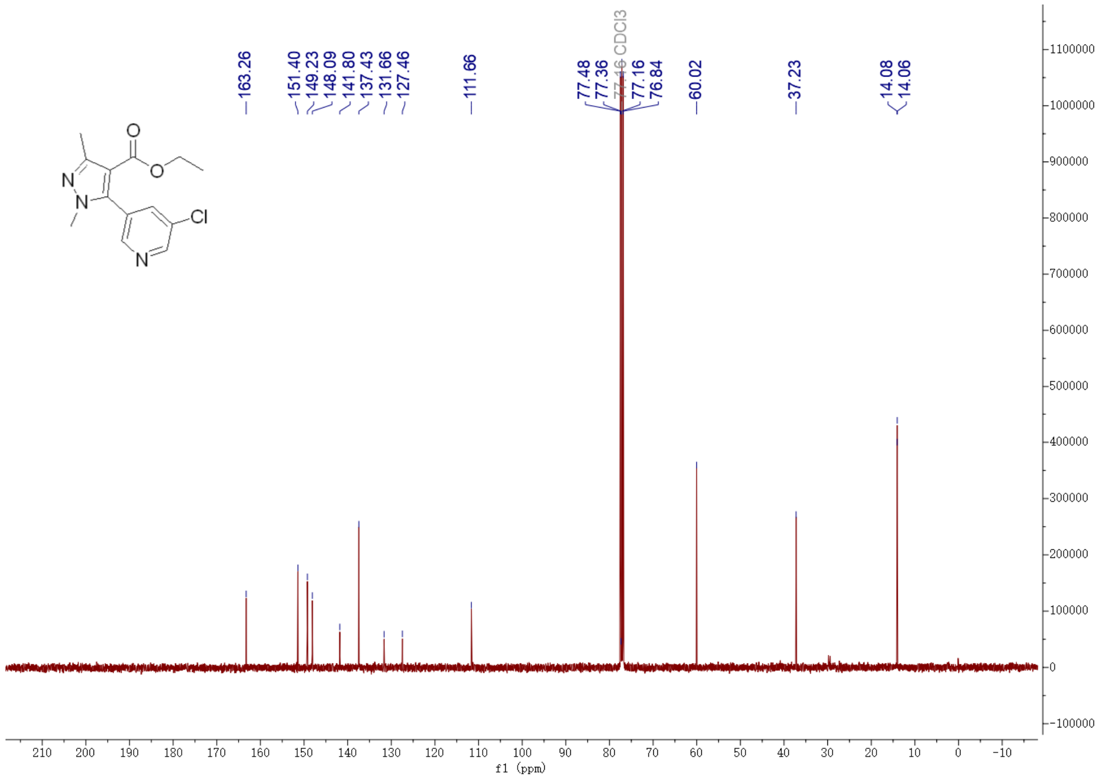

Figure S24. The ^{13}C NMR of **5k** (Chloroform-*d*).

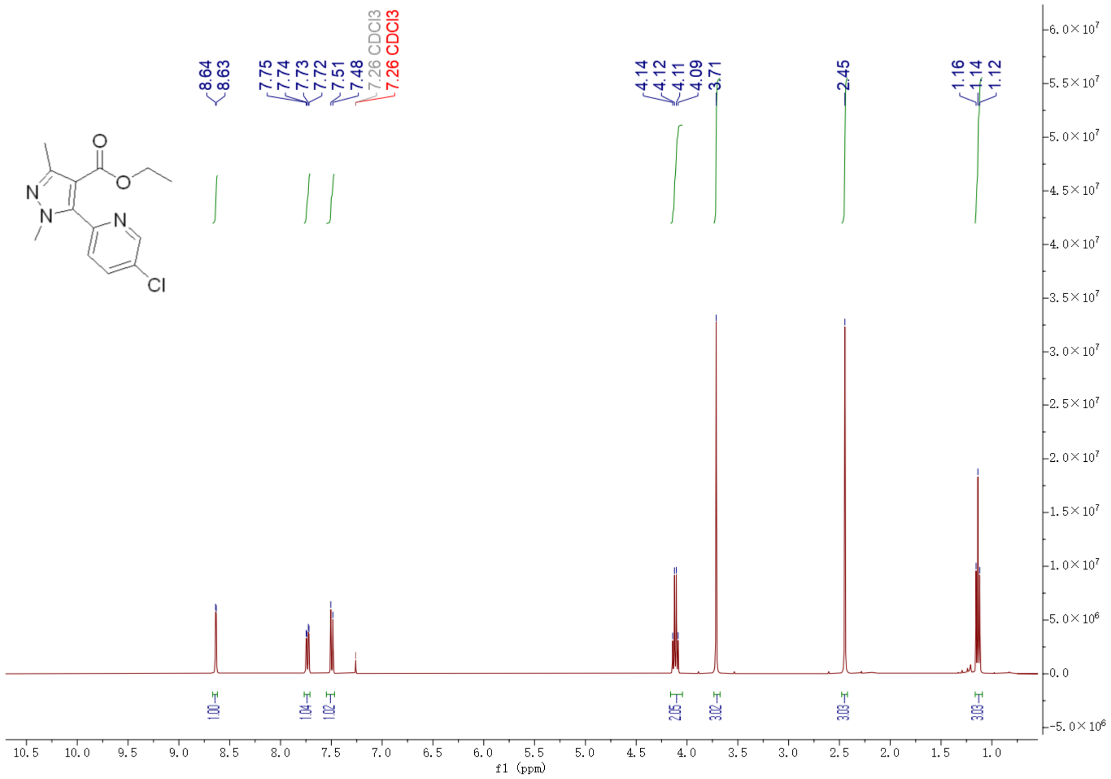

Figure S25. The ^{1}H NMR of **5l** (Chloroform-*d*).

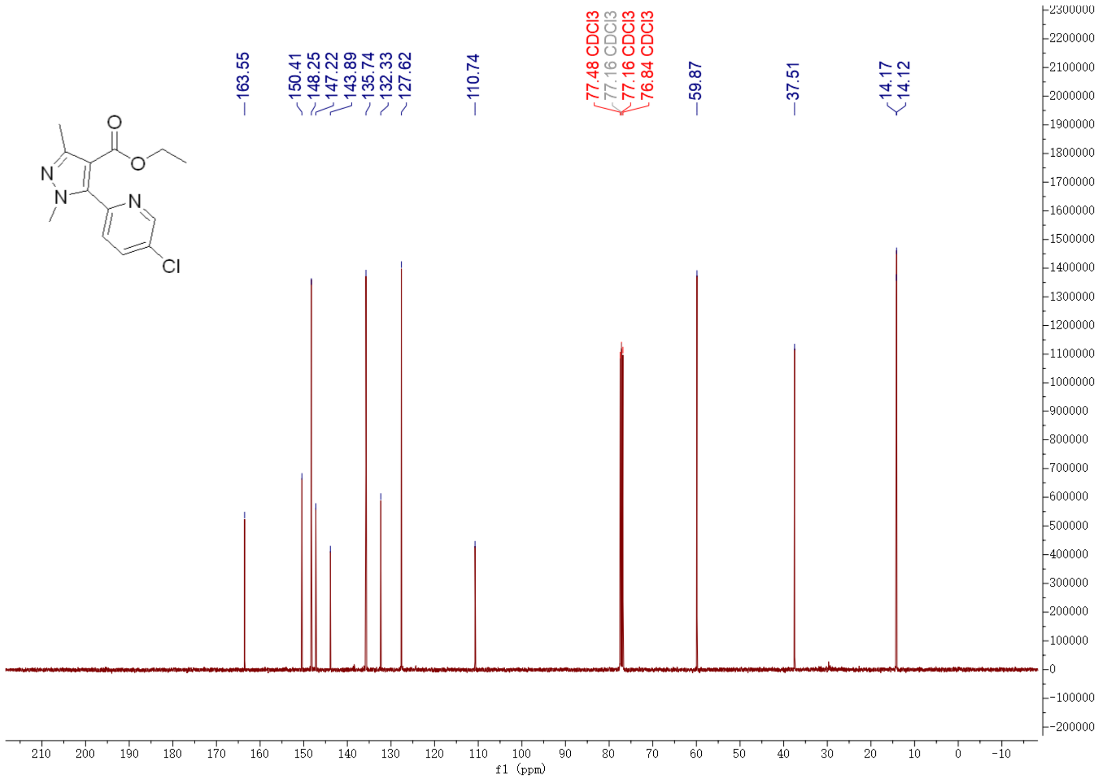

Figure S26. The ^{13}C NMR of **5l** (Chloroform-*d*).

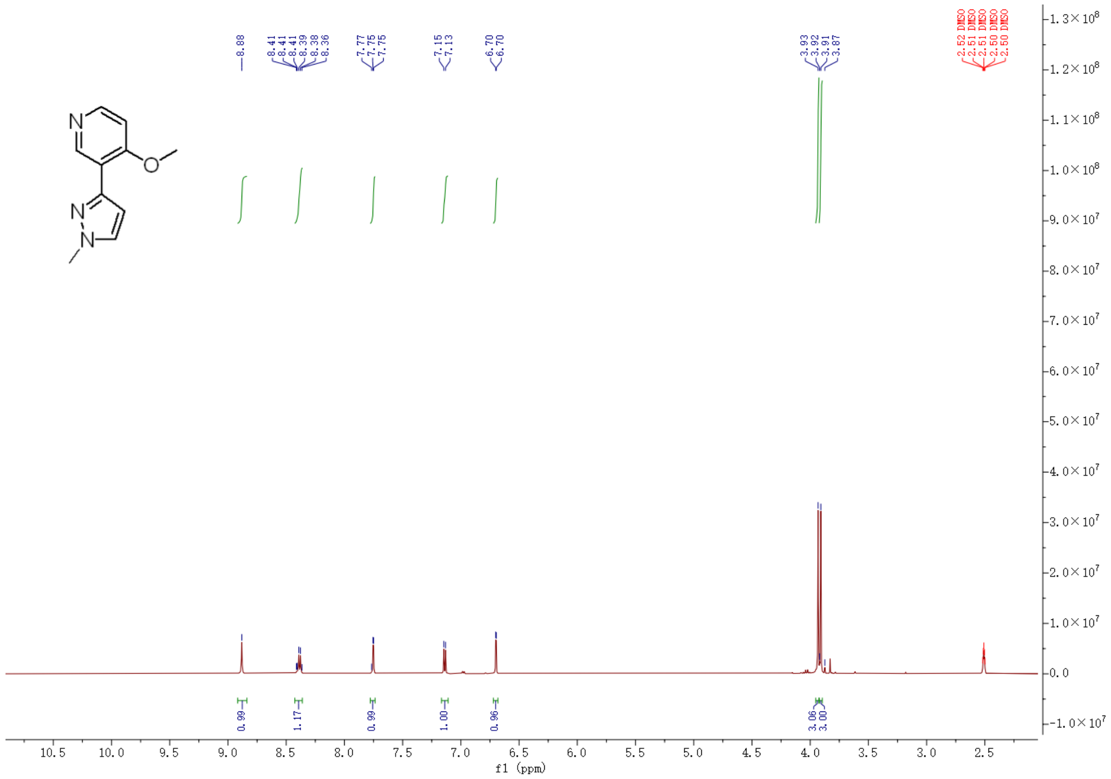

Figure S27. The ^{1}H NMR of **5o** (DMSO-d*6*).

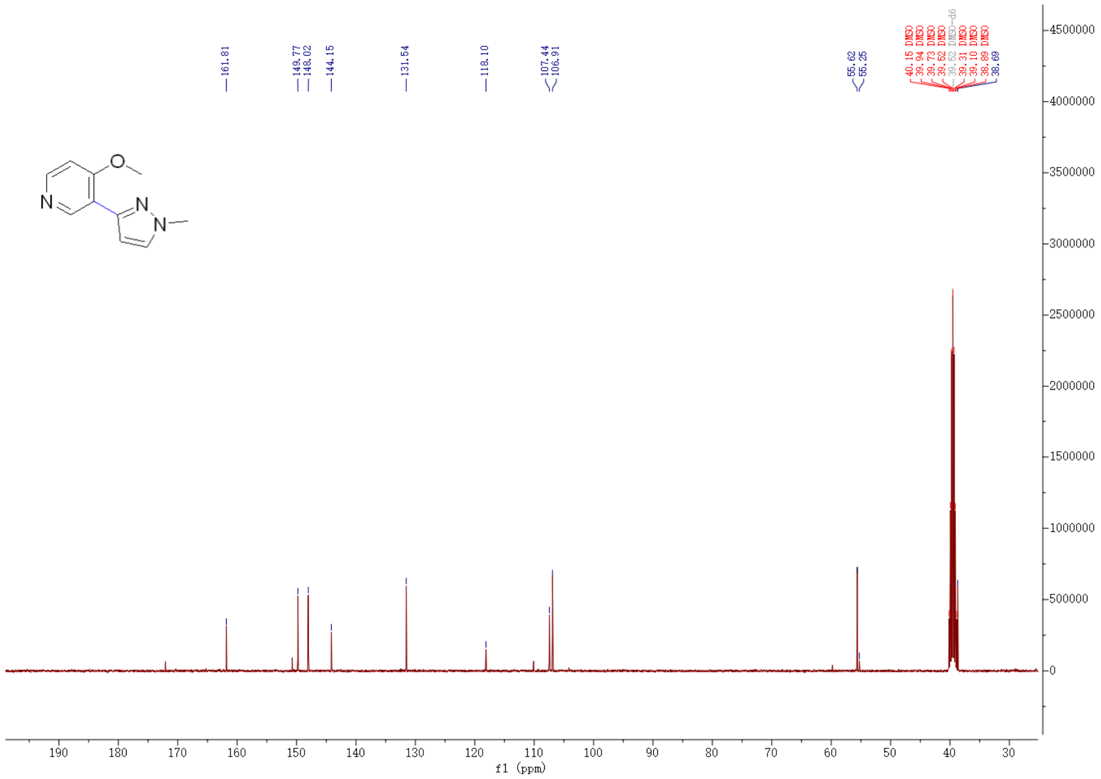

Figure S28. The ^{13}C NMR of **5o** (DMSO-d*6*).

3. ^{1}H NMR and of target compounds 5p–5s;

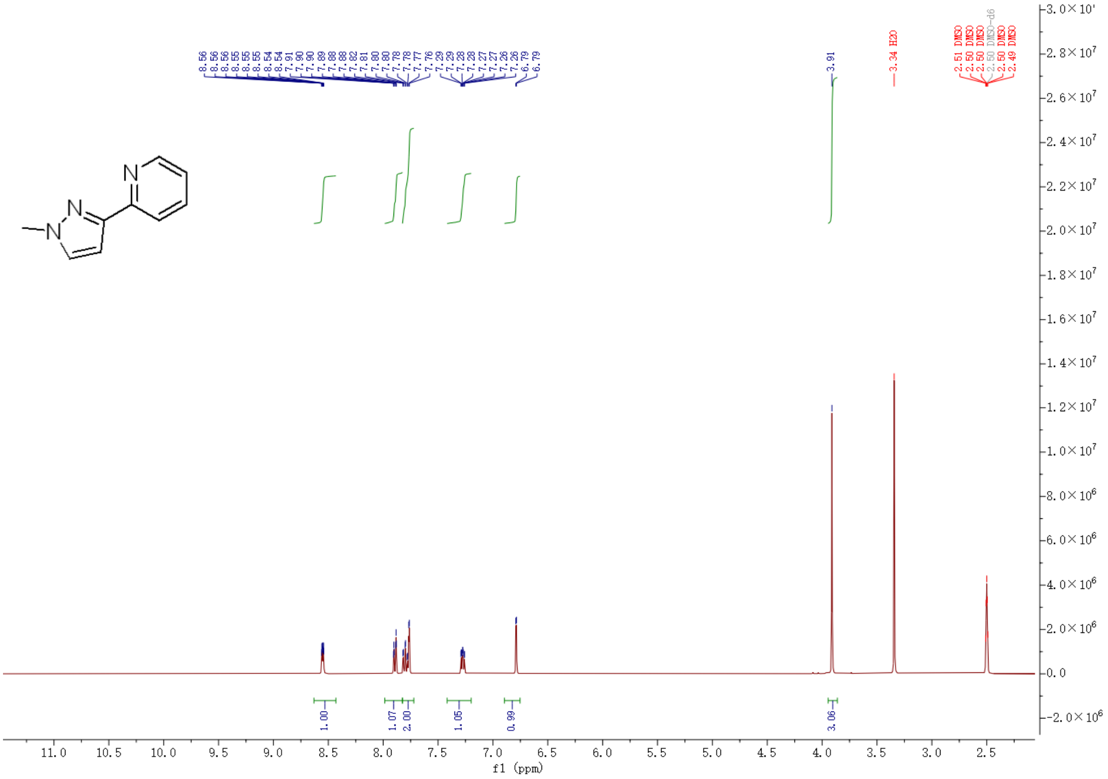

Figure S29. The ^{1}H NMR of **5p** (DMSO-d*6*).

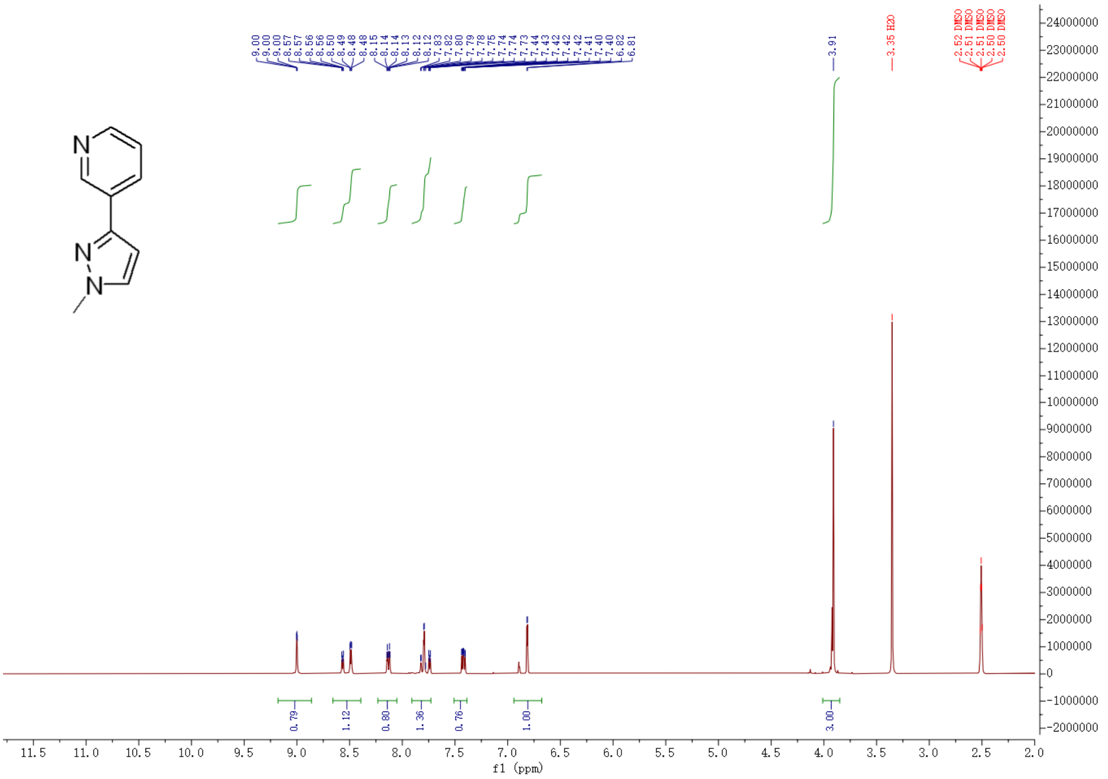

Figure S30. The ^{1}H NMR of **5q** (DMSO-d*6*).

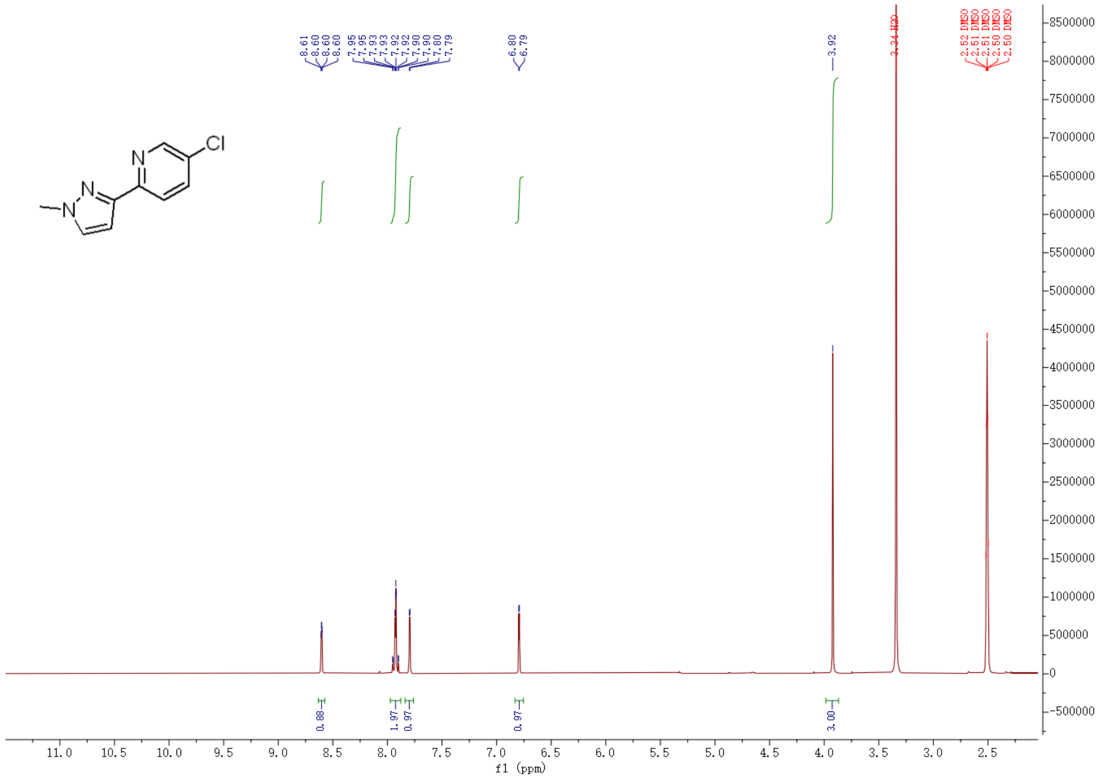

Figure S31. The ^{1}H NMR of **5r** (DMSO-d*6*).

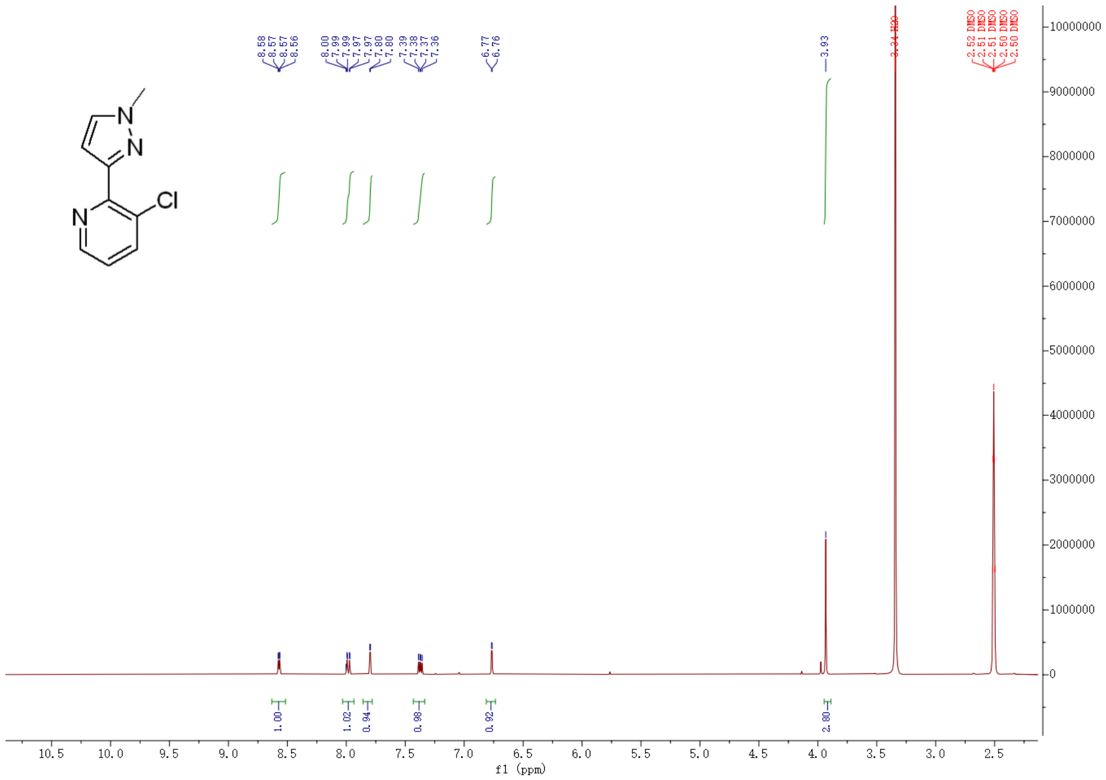

Figure S32. The ^{1}H NMR of **5s** (DMSO-d*6*).

4. LC Chromatogram of Compound 5m;

SCIEX X500R QTOFMS

Ion Source: ESI Ionization mode: +ESI

Ion Source gas 1: 50 psi; Ion Source gas 2: 50 psi; Curtain gas (N2): 30 psi

Ion spray voltage(V): 5500; Temperature(°C): 450

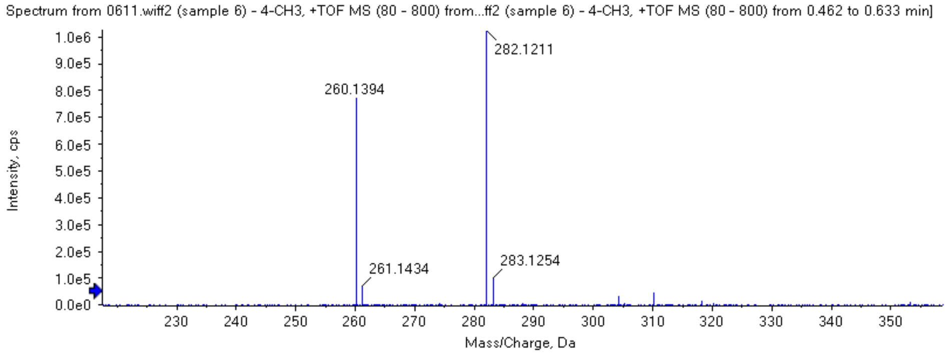

Ion Formula	Calculated m/z	Error (ppm)	Error (mmu)	RDB
C14H18N3O2	260.13935	0.2	0.05	8.0
C14H17N3NaO2	282.12130	-0.8	-0.24	8.0

Figure S33. Direct Injection Chromatogram of **5m**.

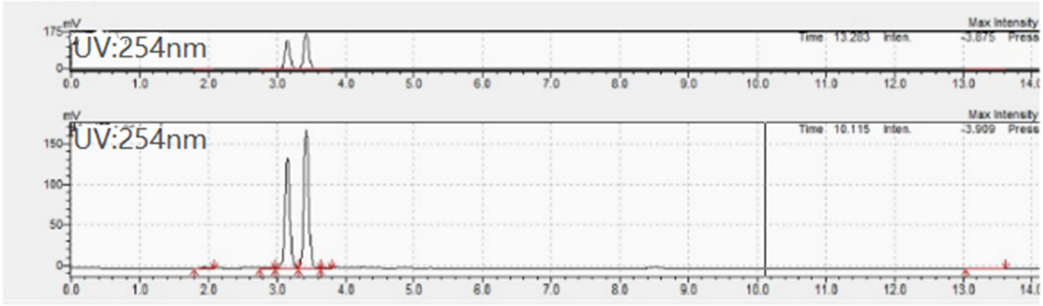

Number	Retention Time (min)	Peak Area	Peak Height	Area (%)
1	1.942	8578	0.613	0.613
2	2.921	10468	0.670	0.670
3	3.152	676221	43.269	43.289
4	3.426	866951	54.868	54.858
5	3.719	7674	0.491	0.491
6	13.263	1223	0.078	0.078
Total		1562115	100.00	100.00

Figure S34. LC Chromatogram of Compound **5m**.

5.^{1}H NMR and ^{13}C NMR of target compounds 8a–8z;

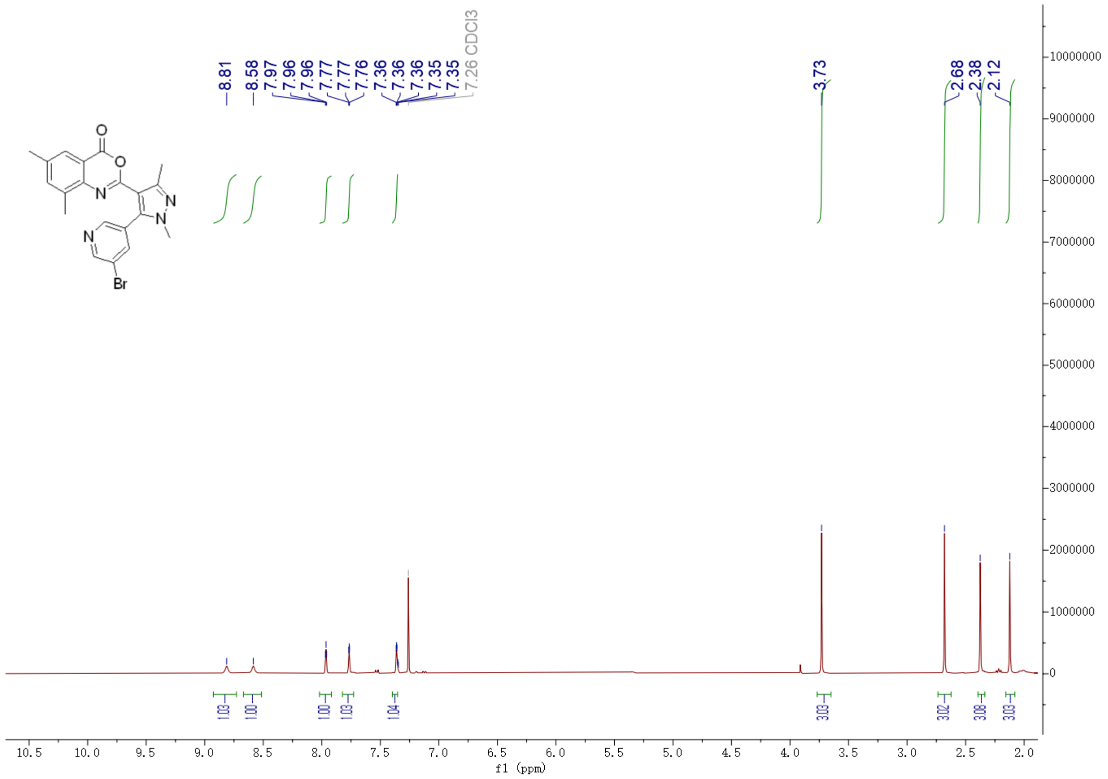

Figure S35. The ^{1}H NMR of **8a** (Chloroform-*d*).

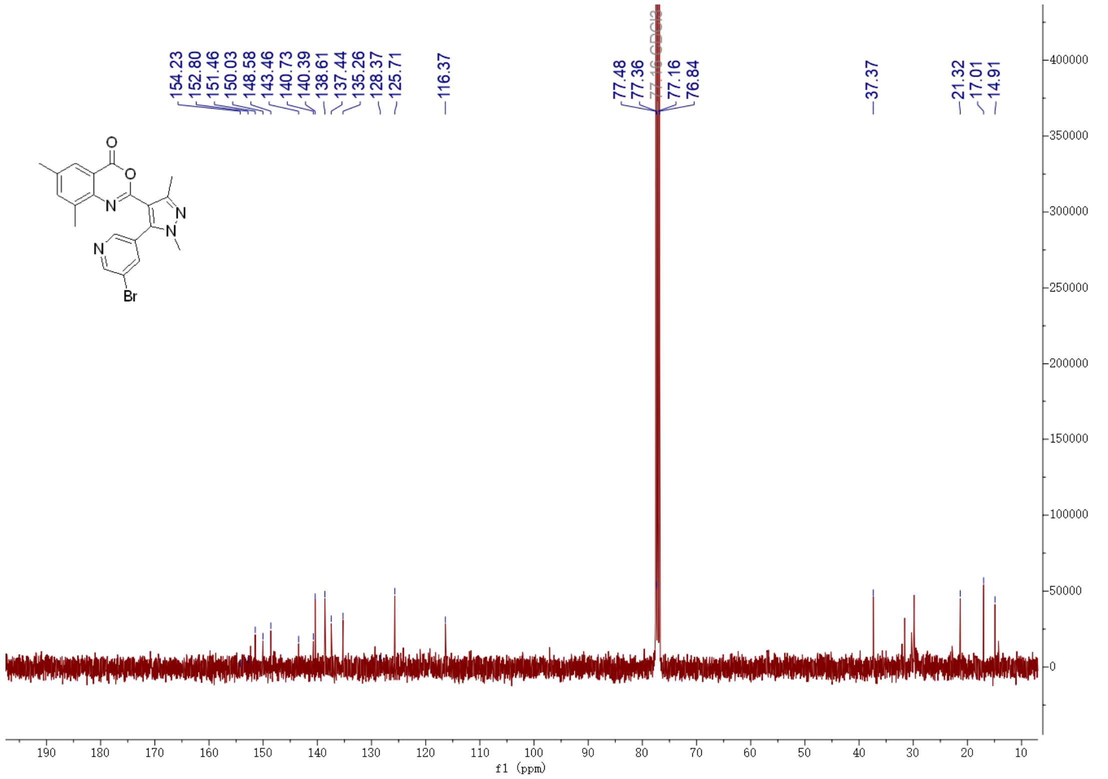

Figure S36. The ^{13}C NMR of **8a** (Chloroform-*d*).

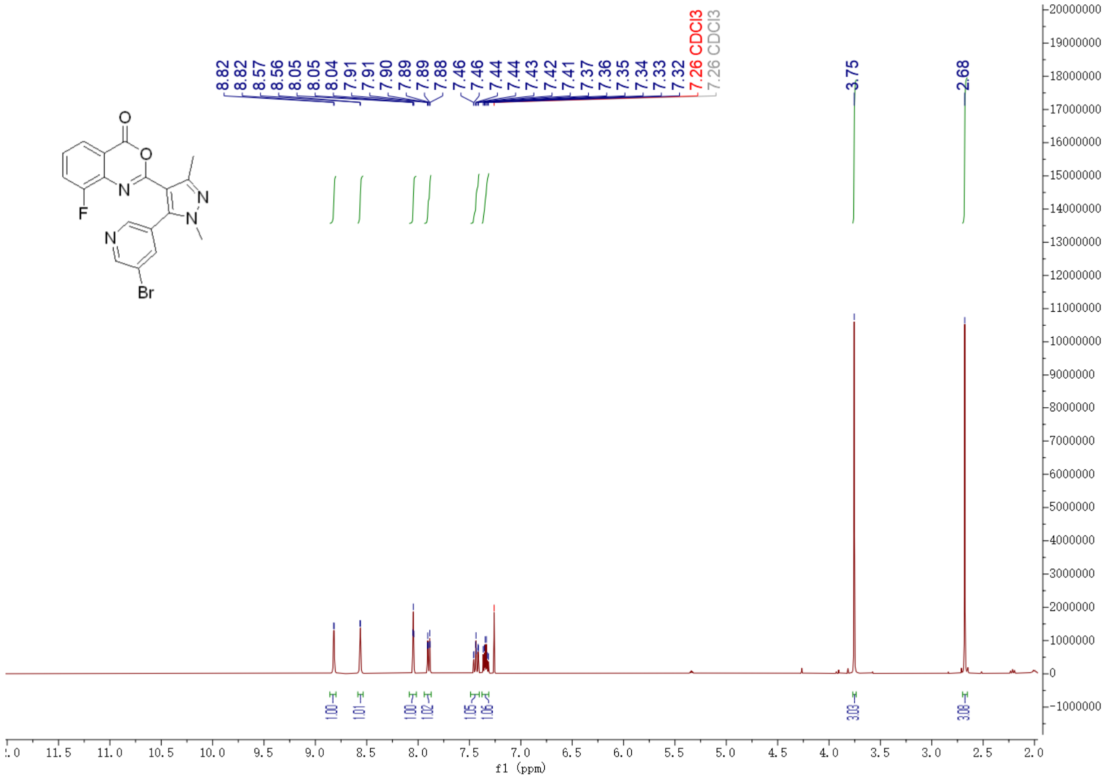

Figure S37. The ^{1}H NMR of **8b** (Chloroform-*d*).

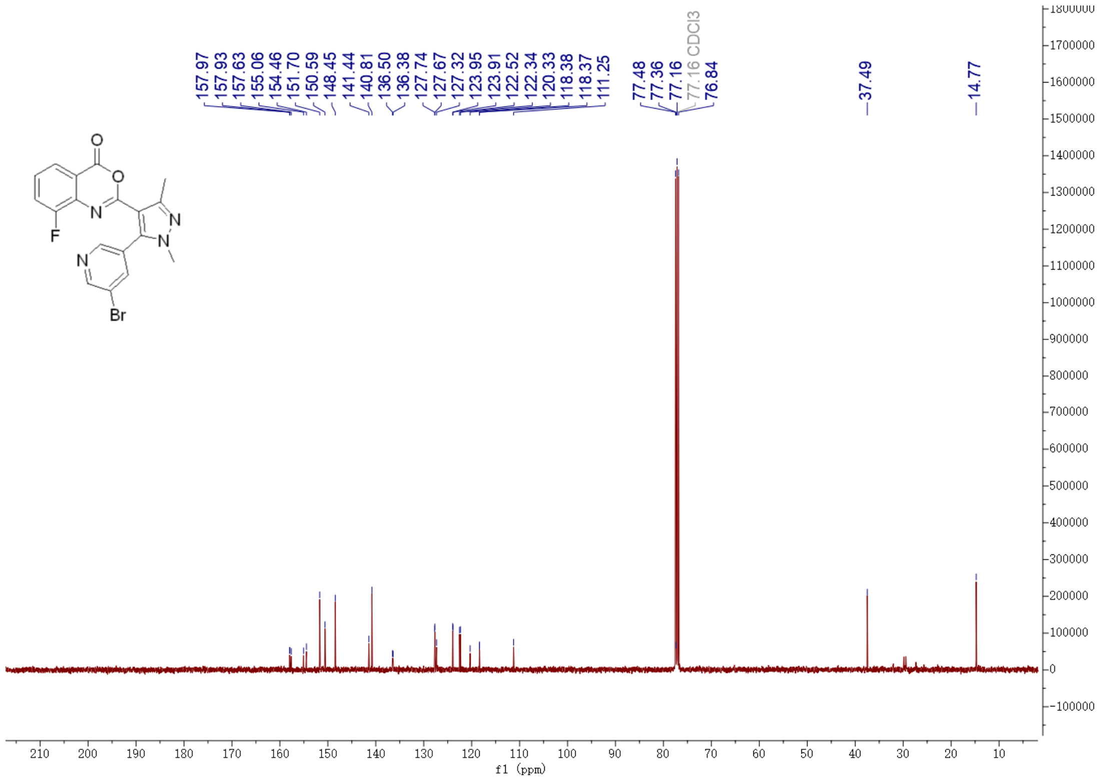

Figure S38. The ^{13}C NMR of **8b** (Chloroform-*d*).

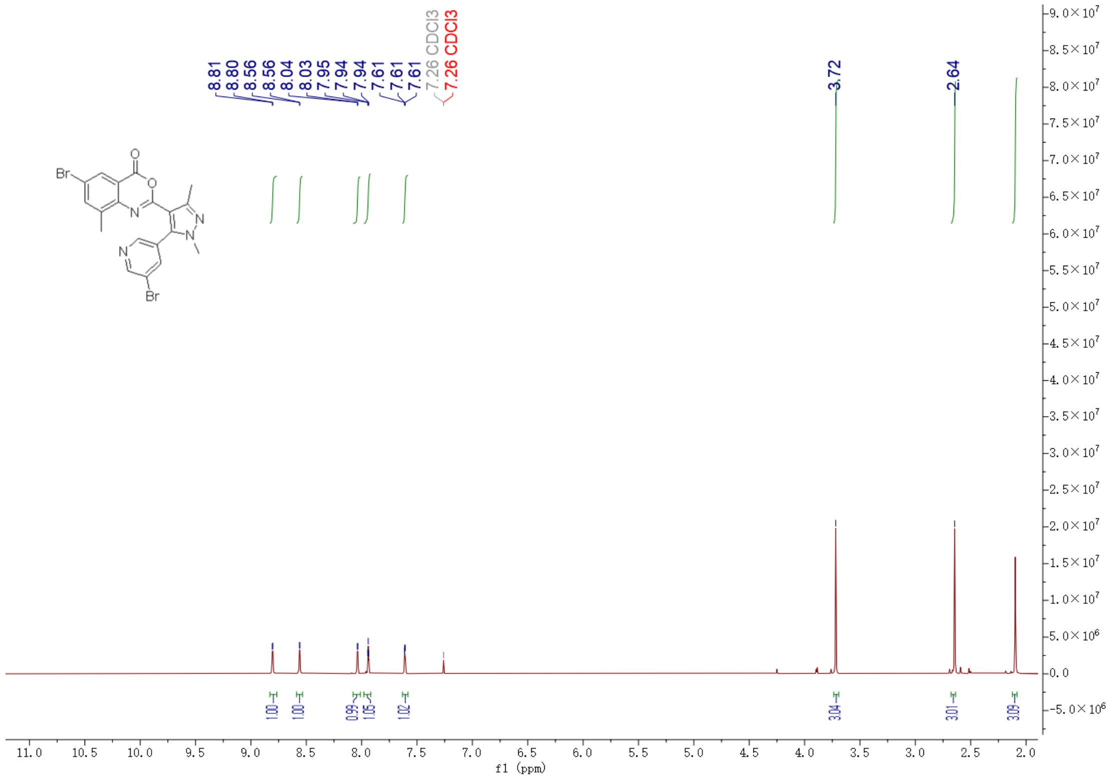

Figure S39. The ^{1}H NMR of **8c** (Chloroform-*d*).

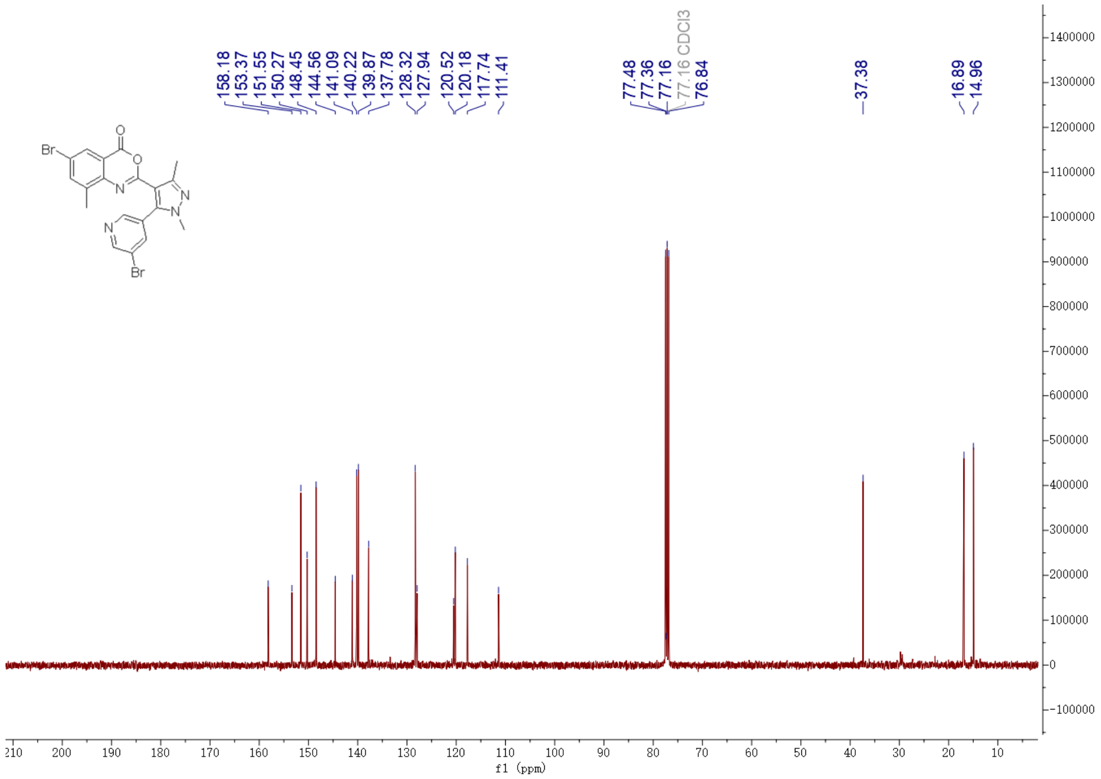

Figure S40. The ^{13}C NMR of **8c** (Chloroform-*d*).

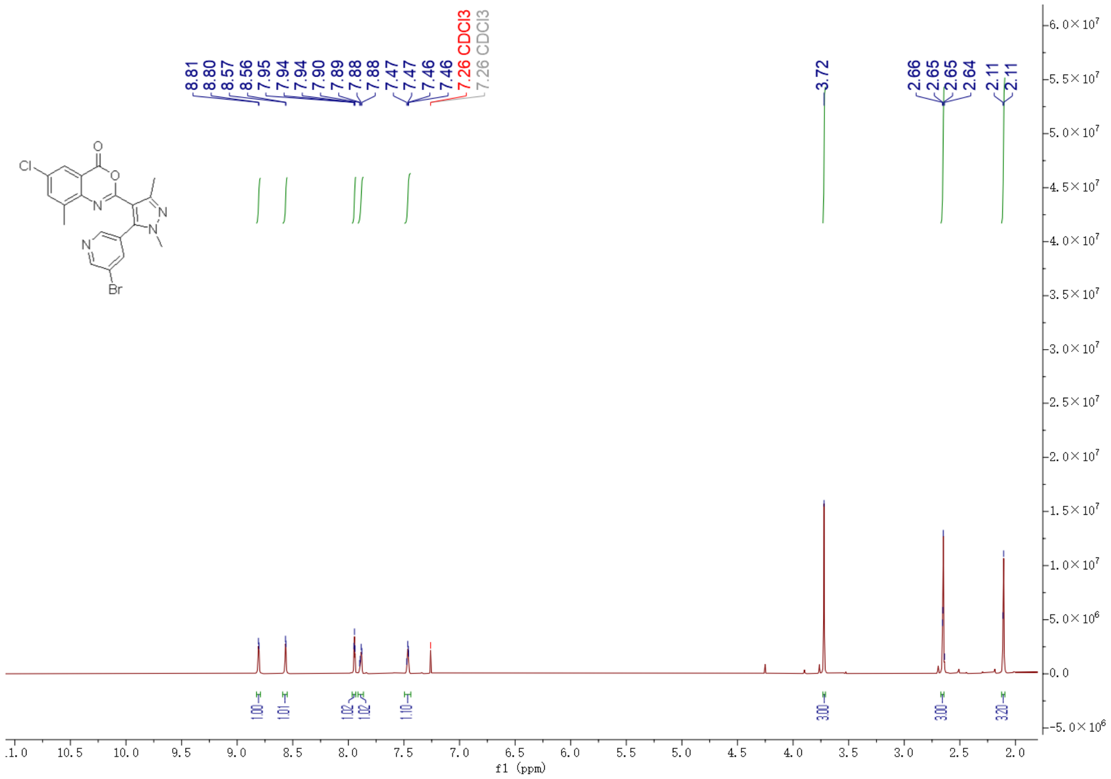

Figure S41. The ^{1}H NMR of **8d** (Chloroform-*d*).

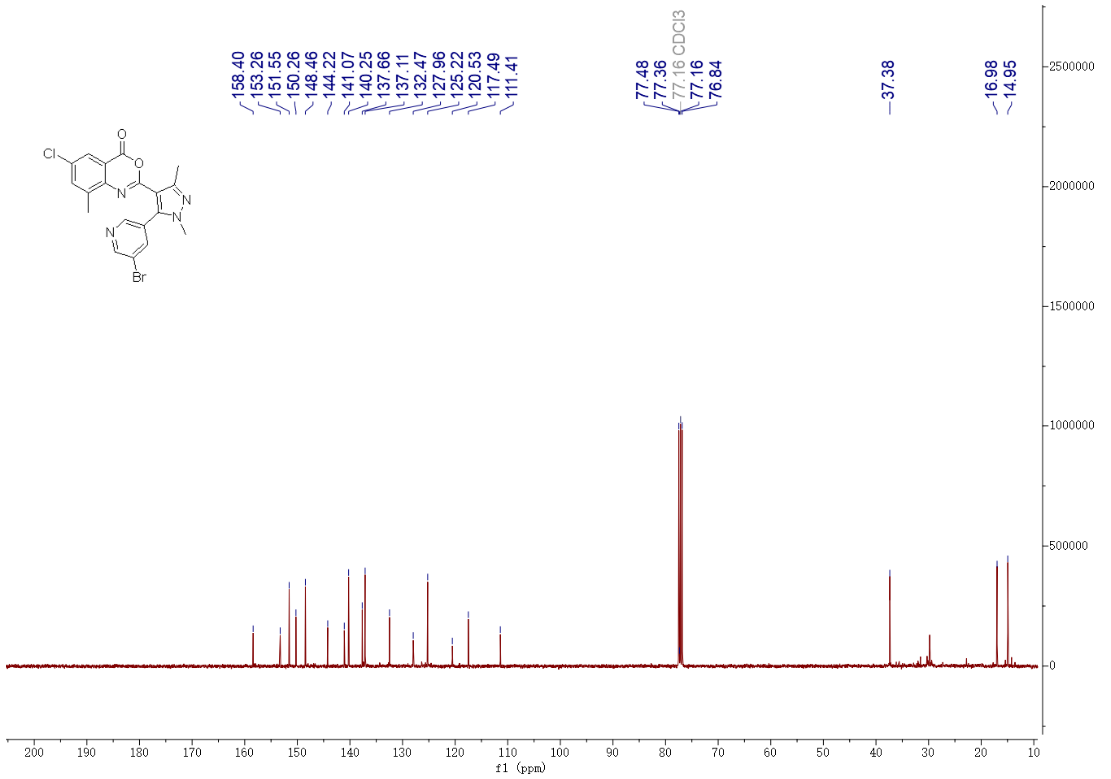

Figure S42. The ^{13}C NMR of **8d** (Chloroform-*d*).

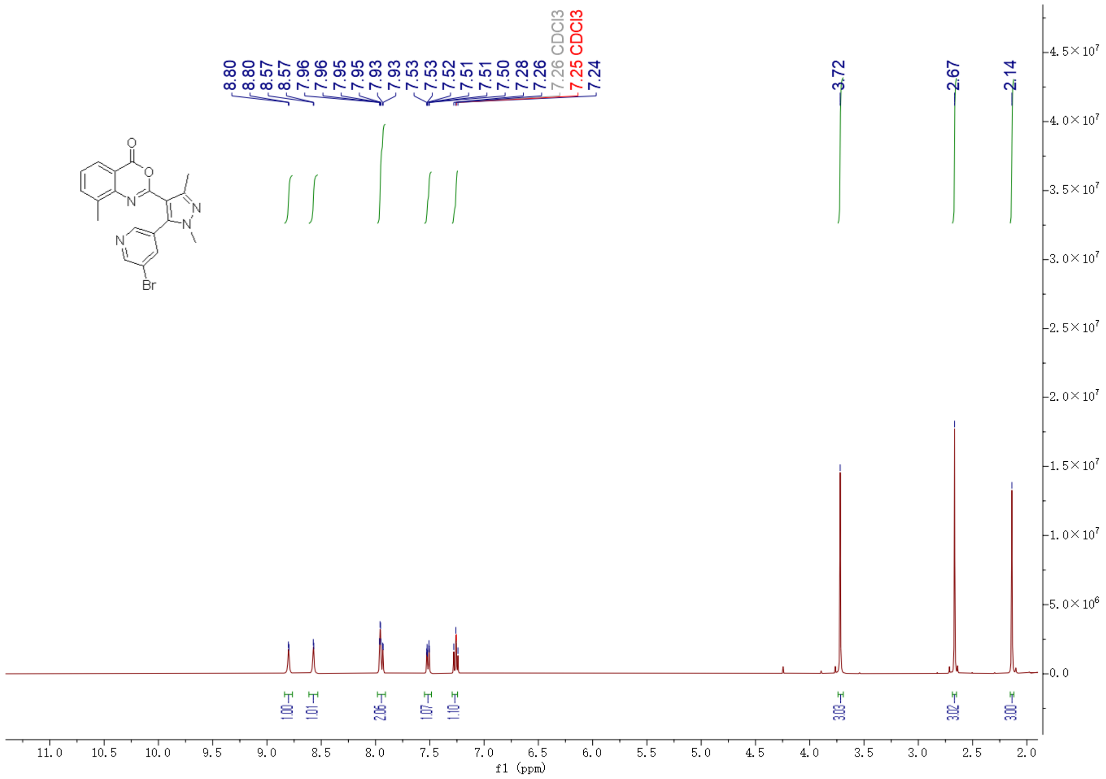

Figure S43. The ^{1}H NMR of **8e** (Chloroform-*d*).

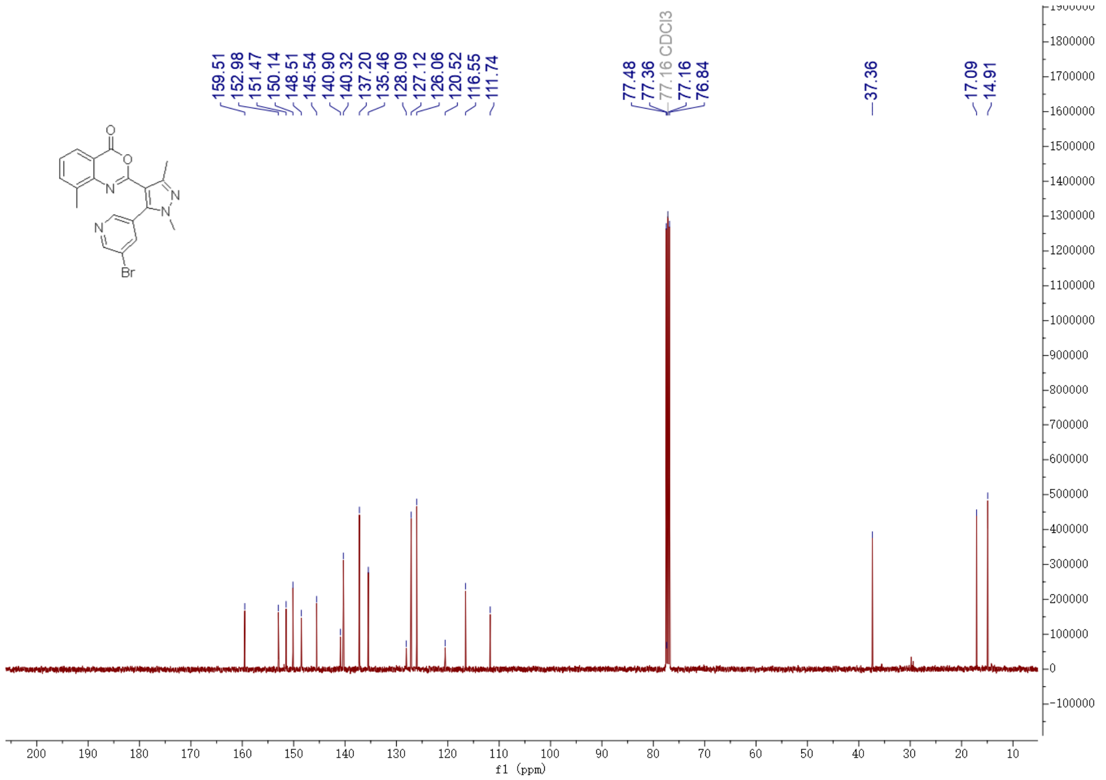

Figure S44. The ^{13}C NMR of **8e** (Chloroform-*d*).

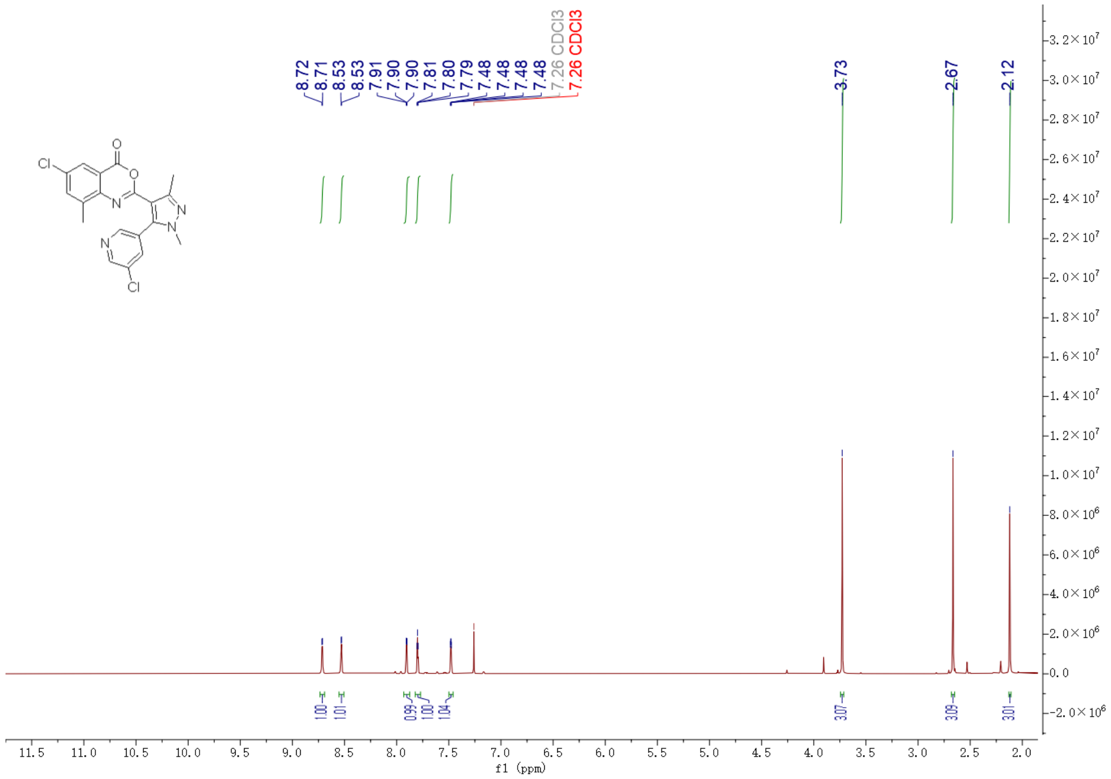

Figure S45. The ^{1}H NMR of **8f** (Chloroform-*d*).

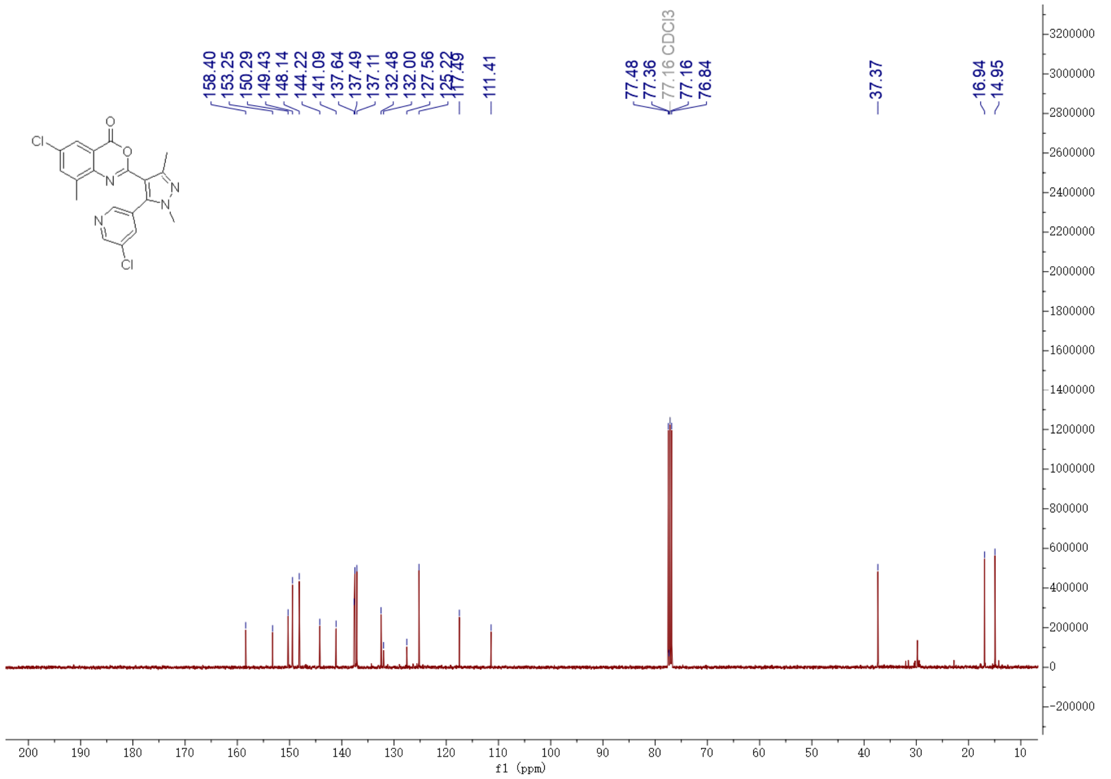

Figure S46. The ^{13}C NMR of **8f** (Chloroform-*d*).

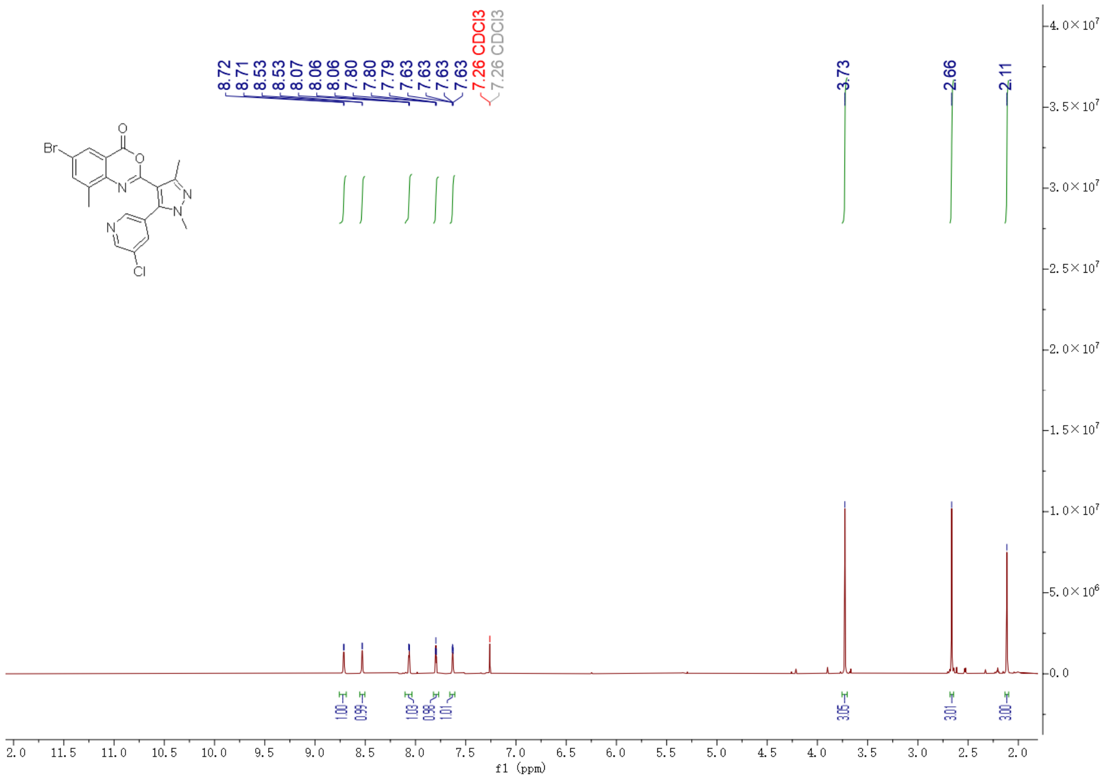

Figure S47. The ^{1}H NMR of **8g** (Chloroform-*d*).

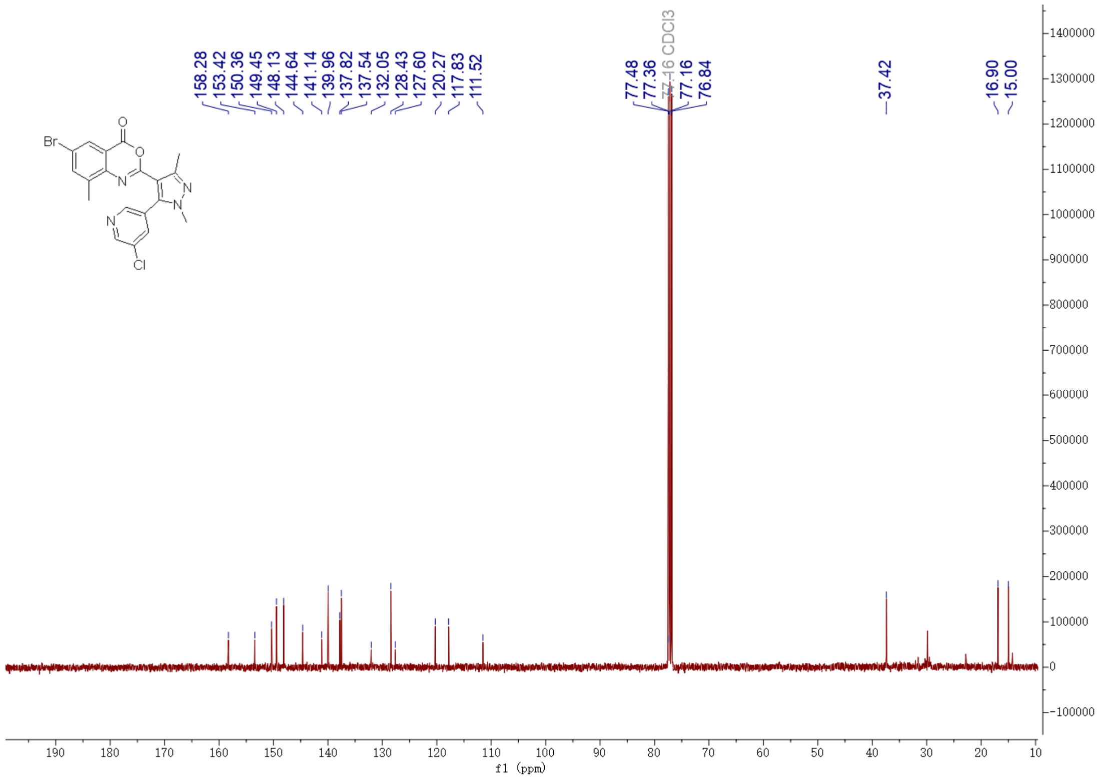

Figure S48. The ^{13}C NMR of **8g** (Chloroform-*d*)

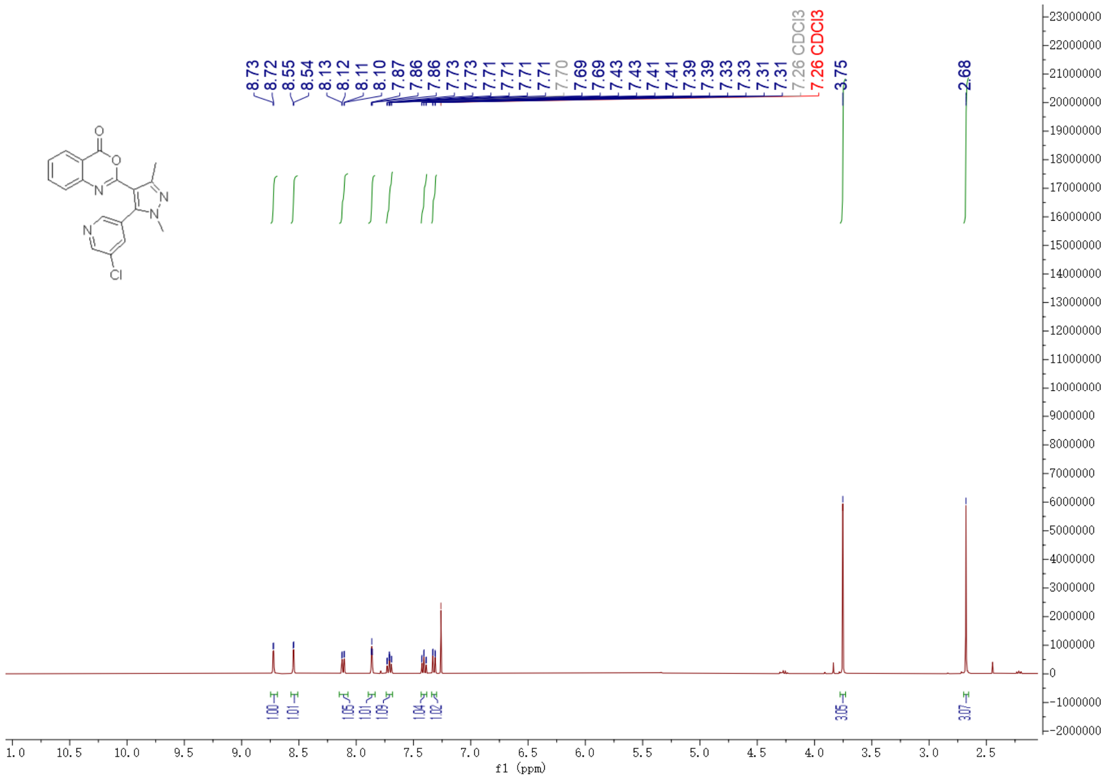

Figure S49. The ^{1}H NMR of **8h** (Chloroform-*d*).

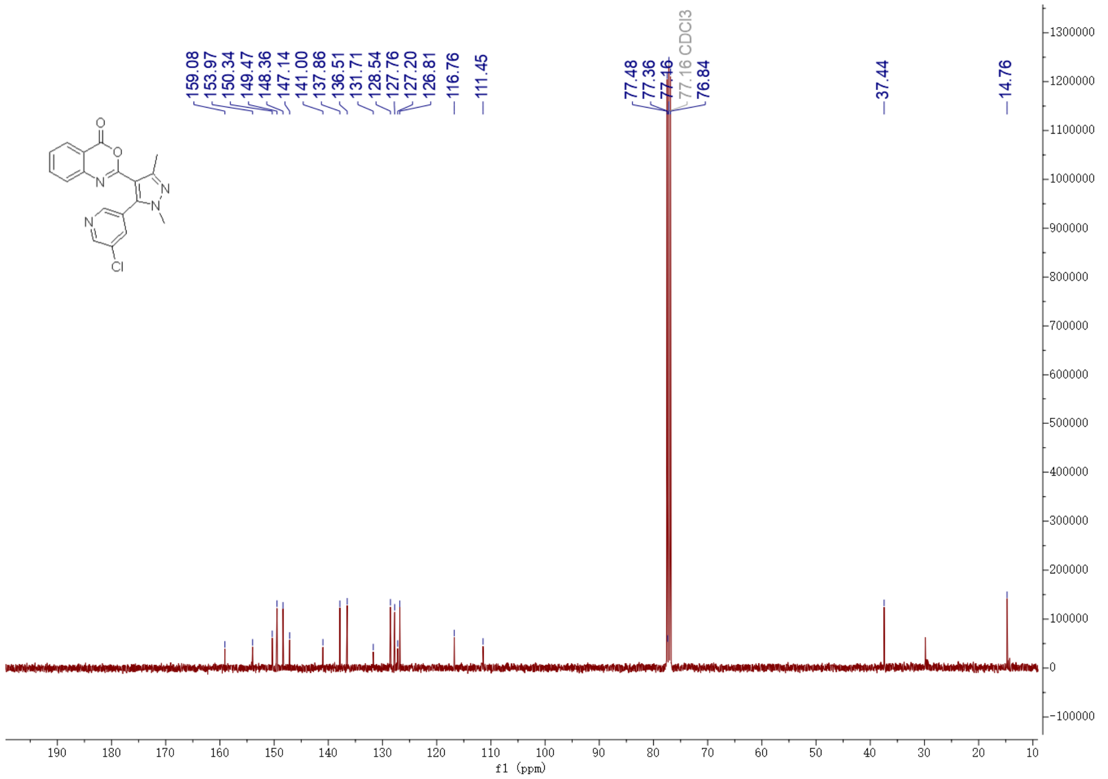

Figure S50. The ^{13}C NMR of **8h** (Chloroform-*d*)

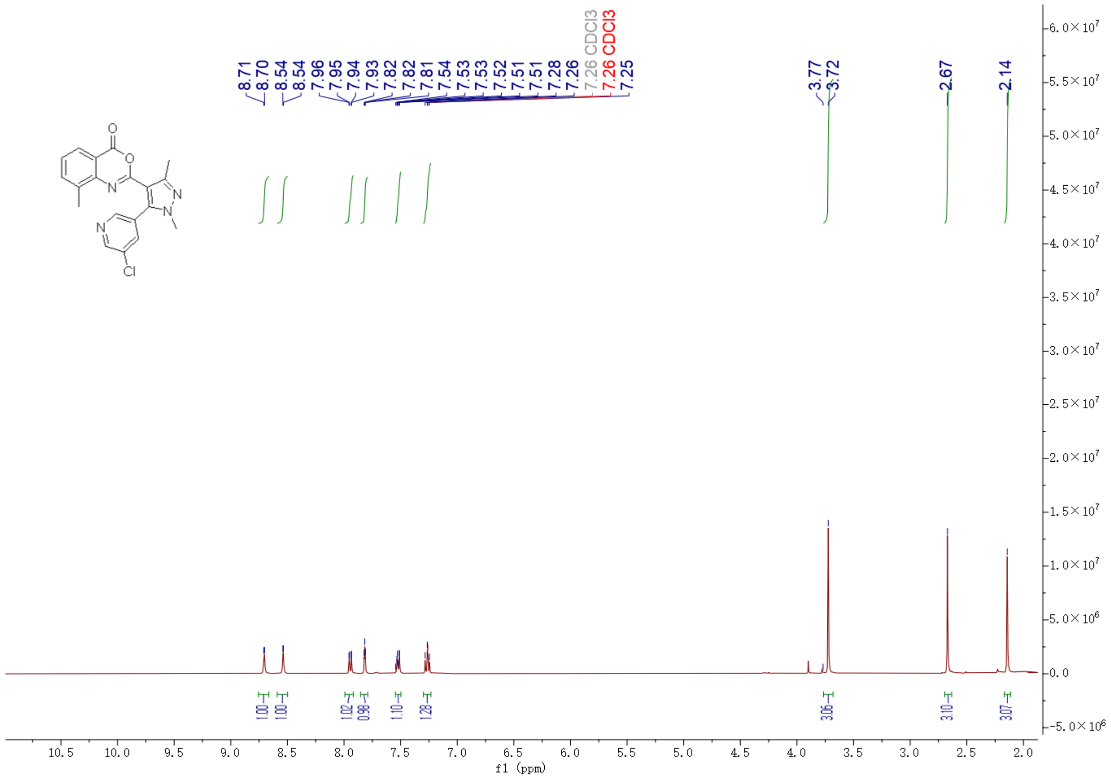

Figure S51. The ^{1}H NMR of **8i** (Chloroform-*d*).

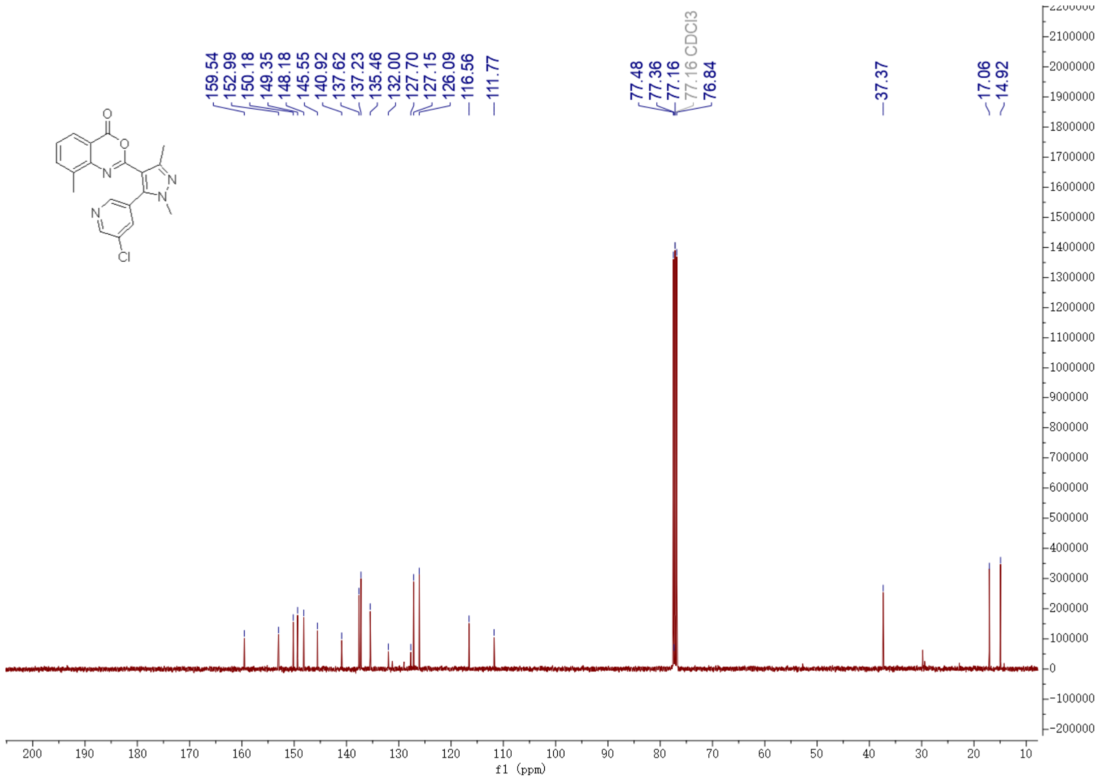

Figure S52. The ^{13}C NMR of **8i** (Chloroform-*d*)

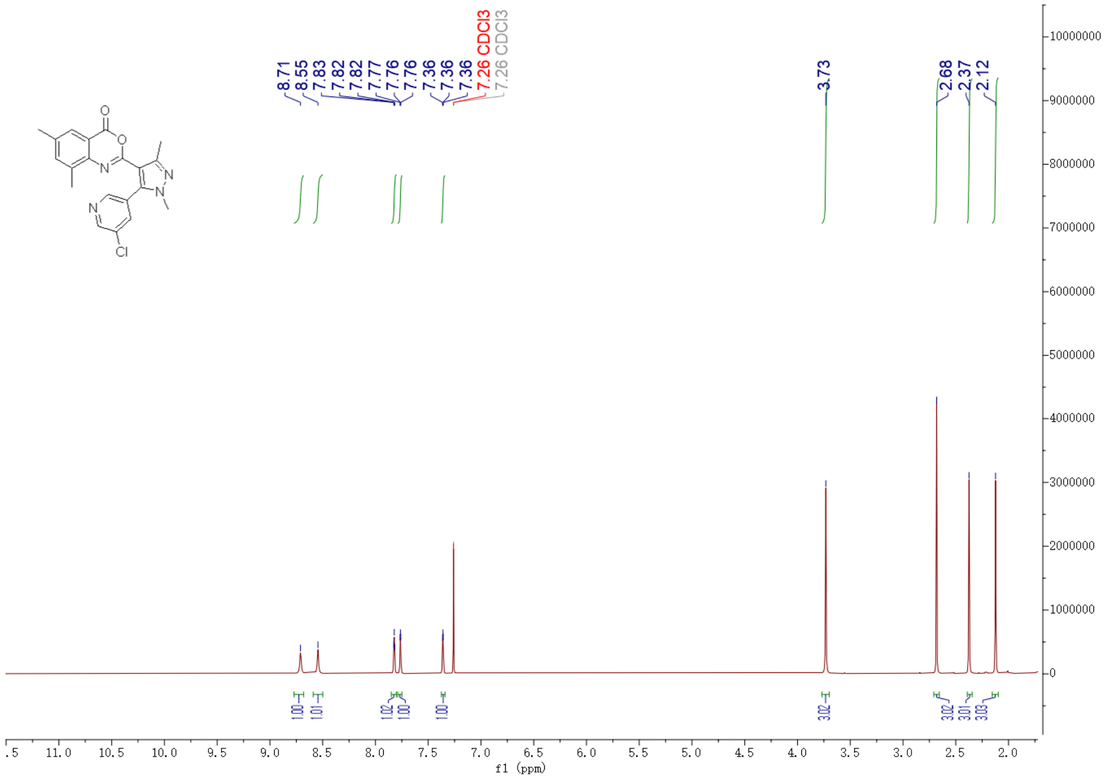

Figure S53. The ^{1}H NMR of **8j** (Chloroform-*d*).

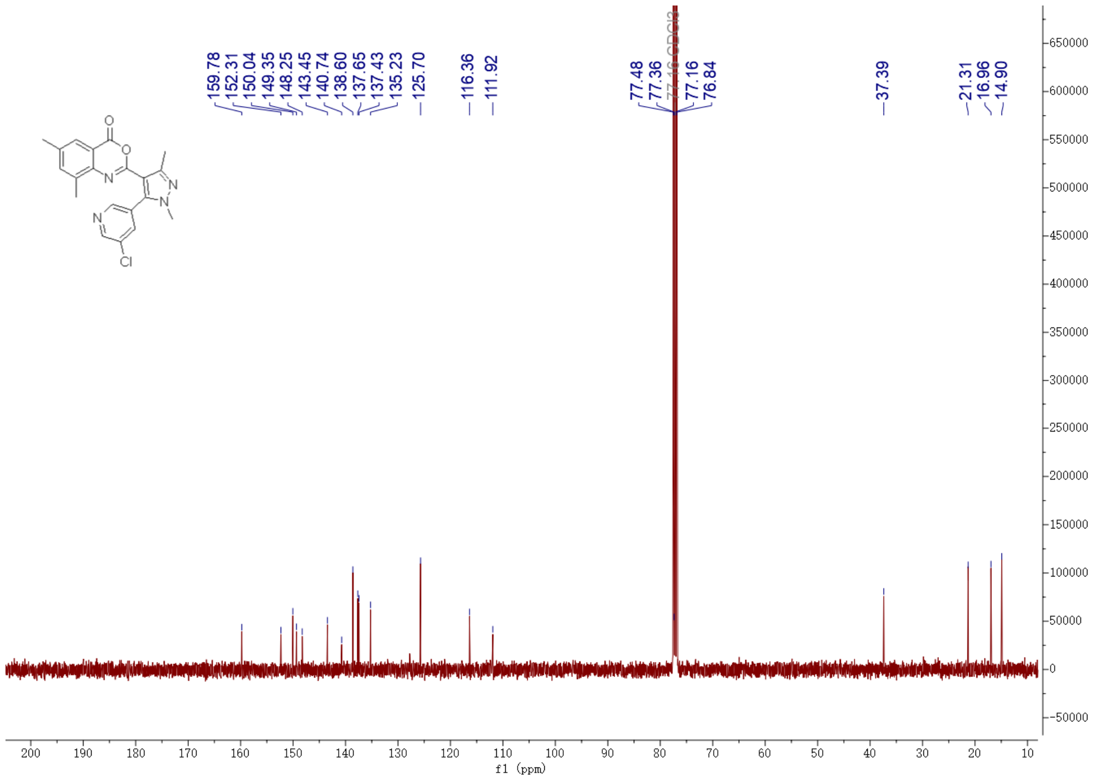

Figure S54. The ^{13}C NMR of **8j** (Chloroform-*d*)

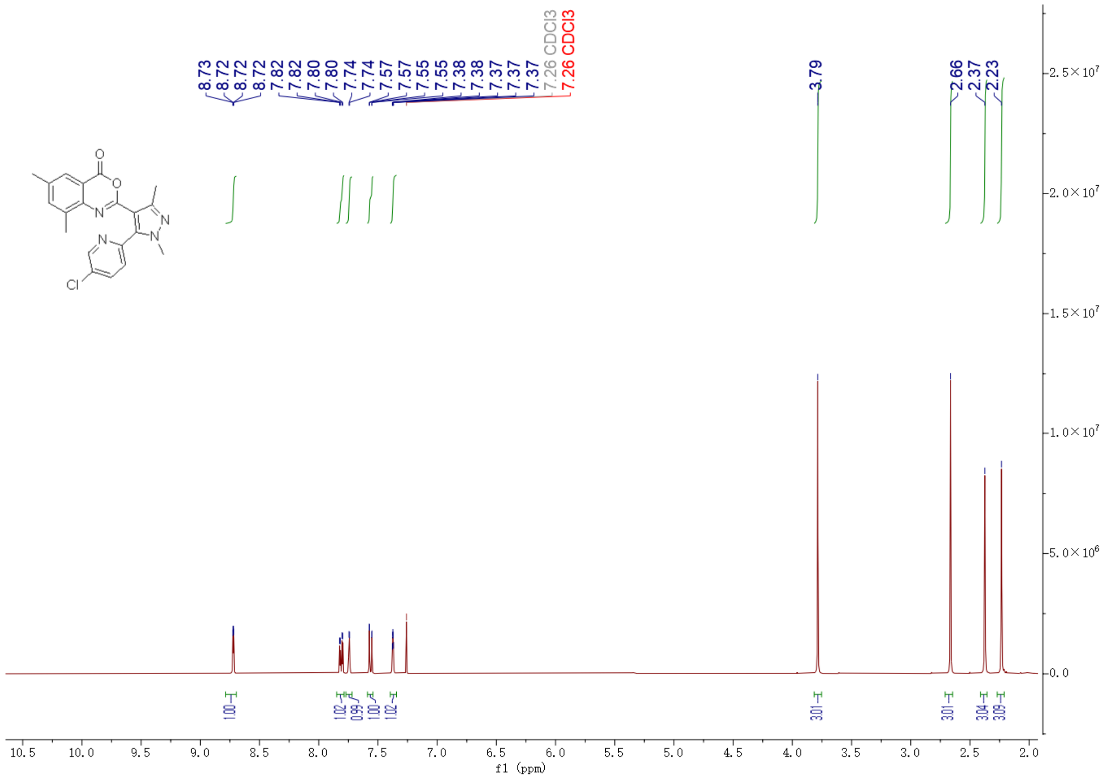

Figure S55. The ^{1}H NMR of **8k** (Chloroform-*d*).

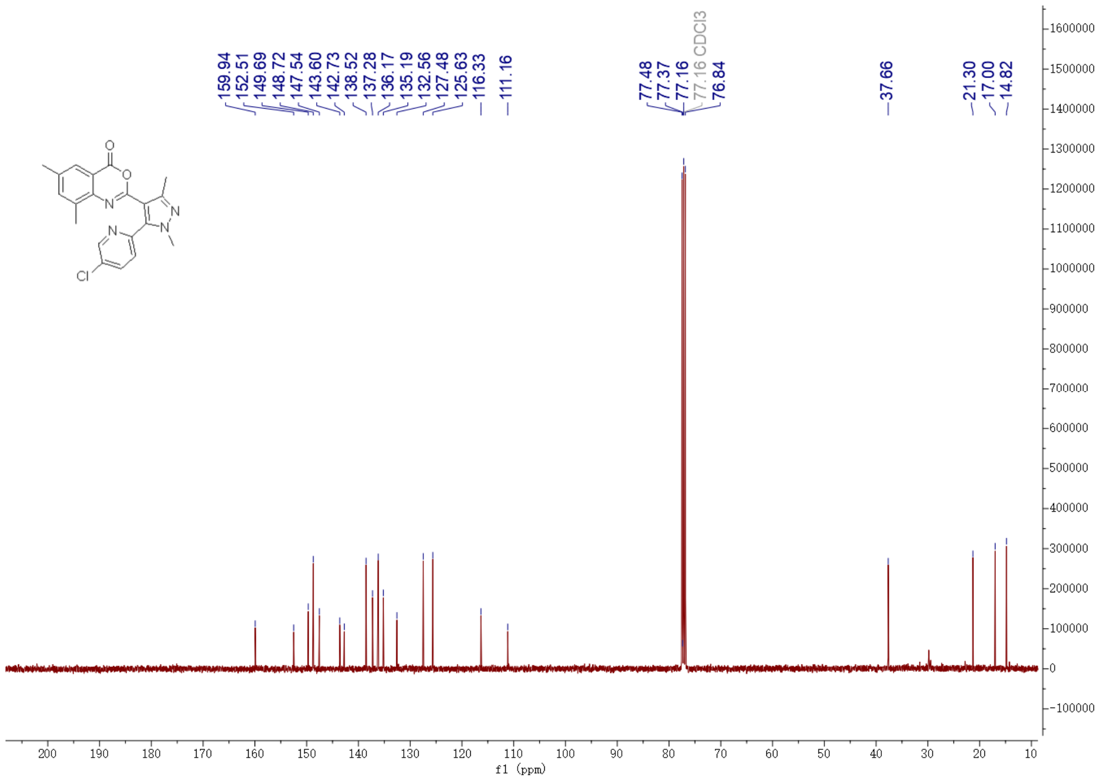

Figure S56. The ^{13}C NMR of **8k** (Chloroform-*d*)

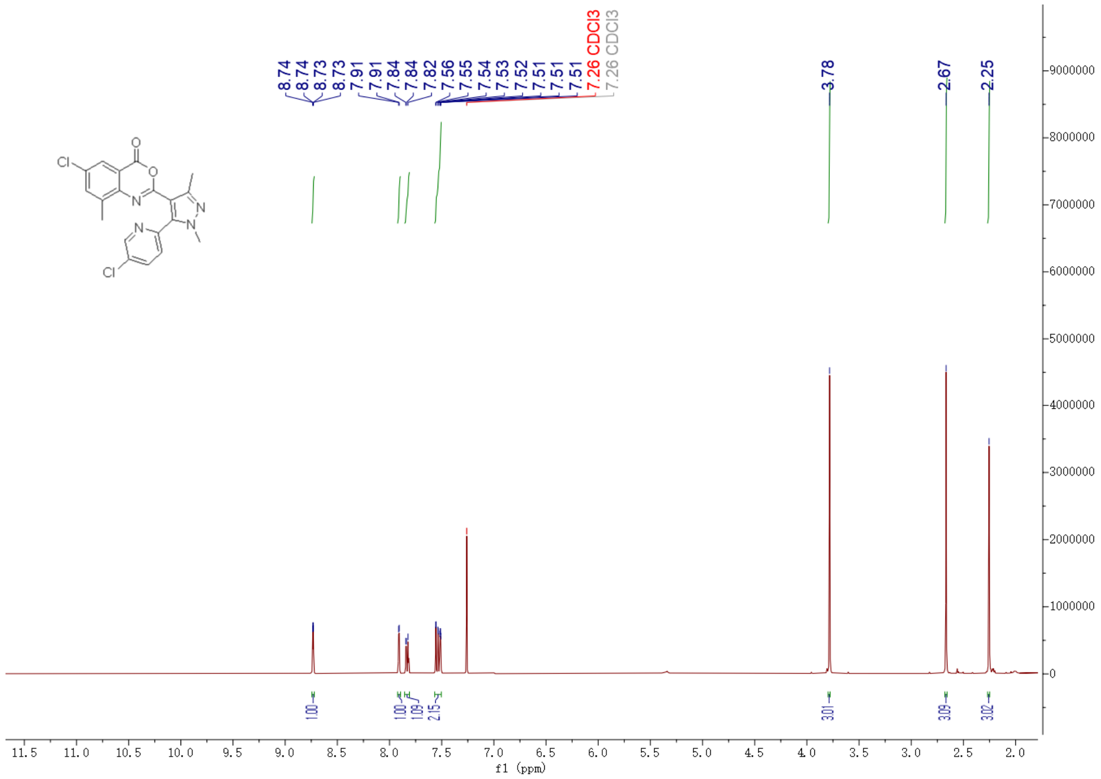

Figure S57. The ^{1}H NMR of **8l** (Chloroform-*d*).

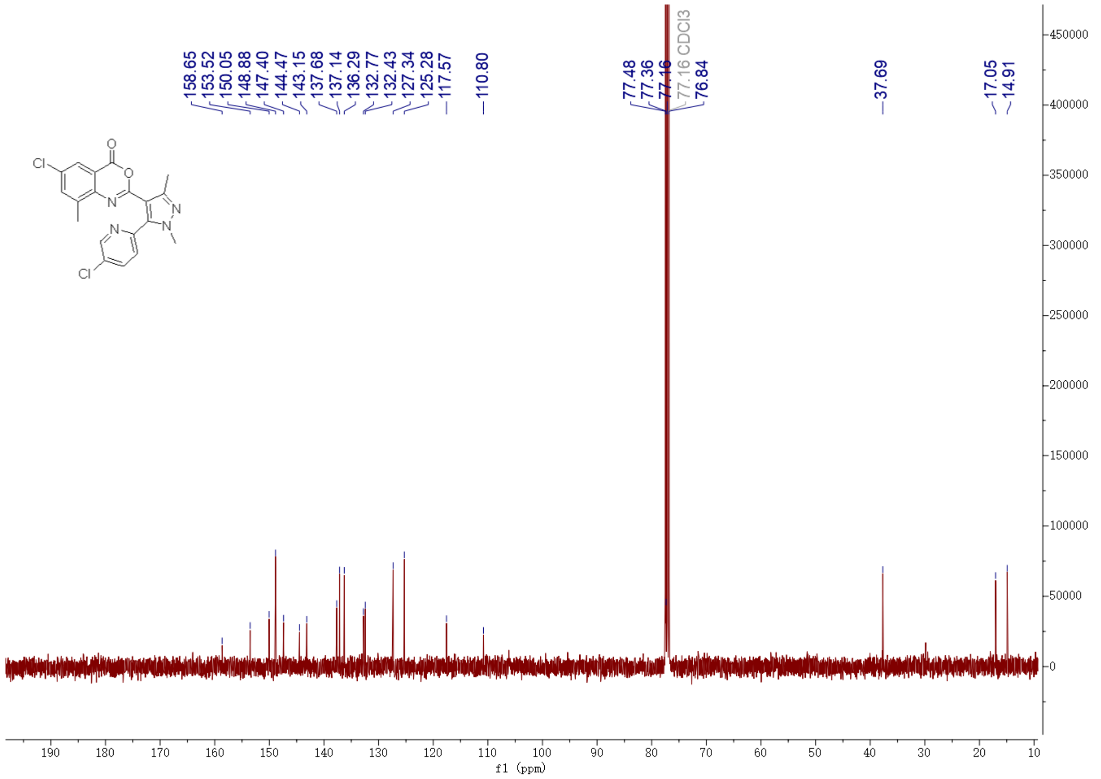

Figure S58. The ^{13}C NMR of **8l** (Chloroform-*d*)

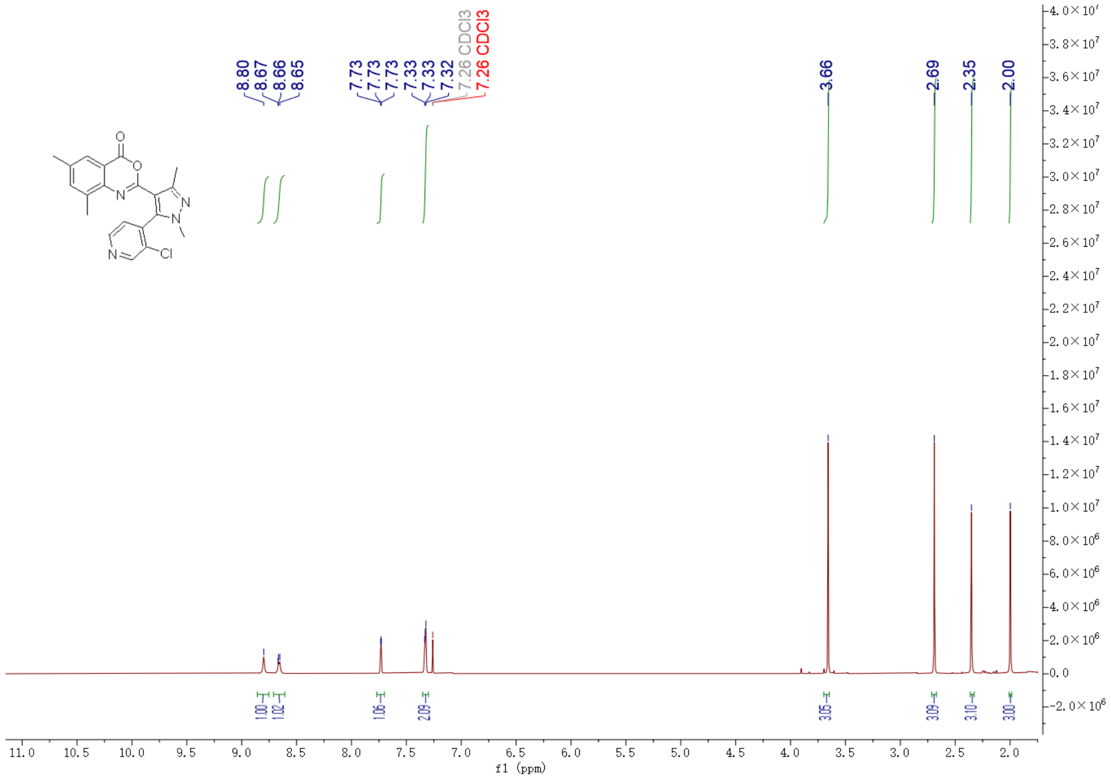

Figure S59. The ^{1}H NMR of **8m** (Chloroform-*d*).

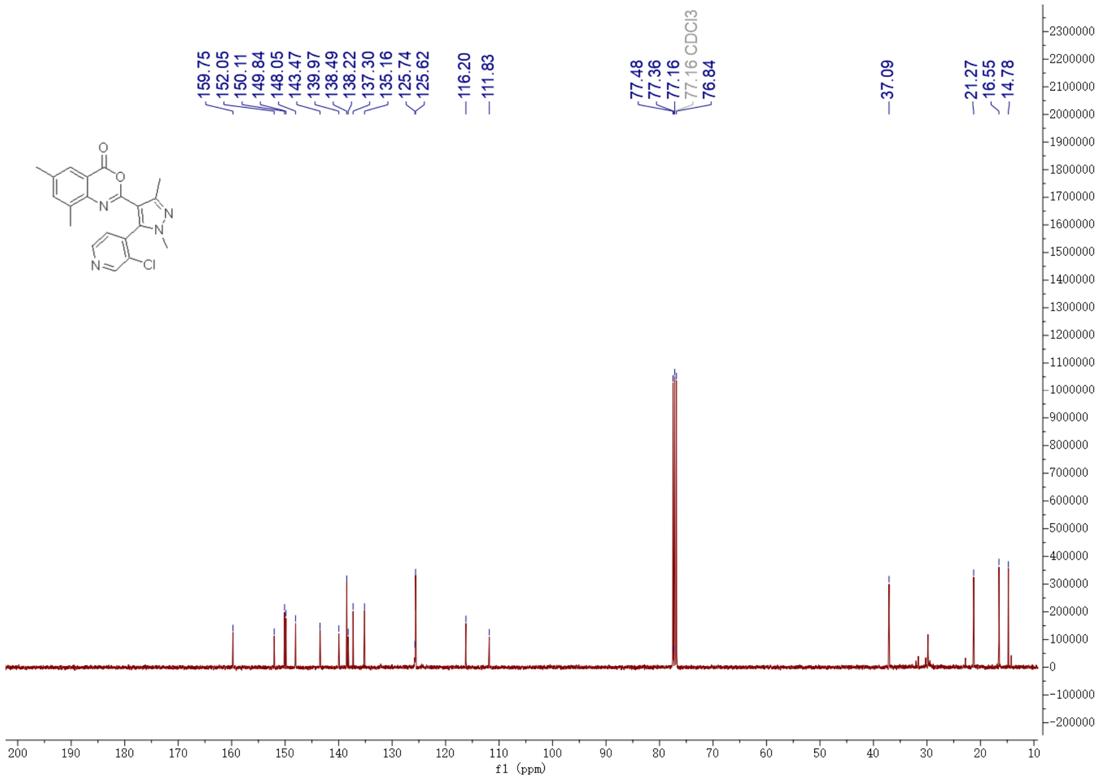

Figure S60. The ^{13}C NMR of **8m** (Chloroform-*d*)

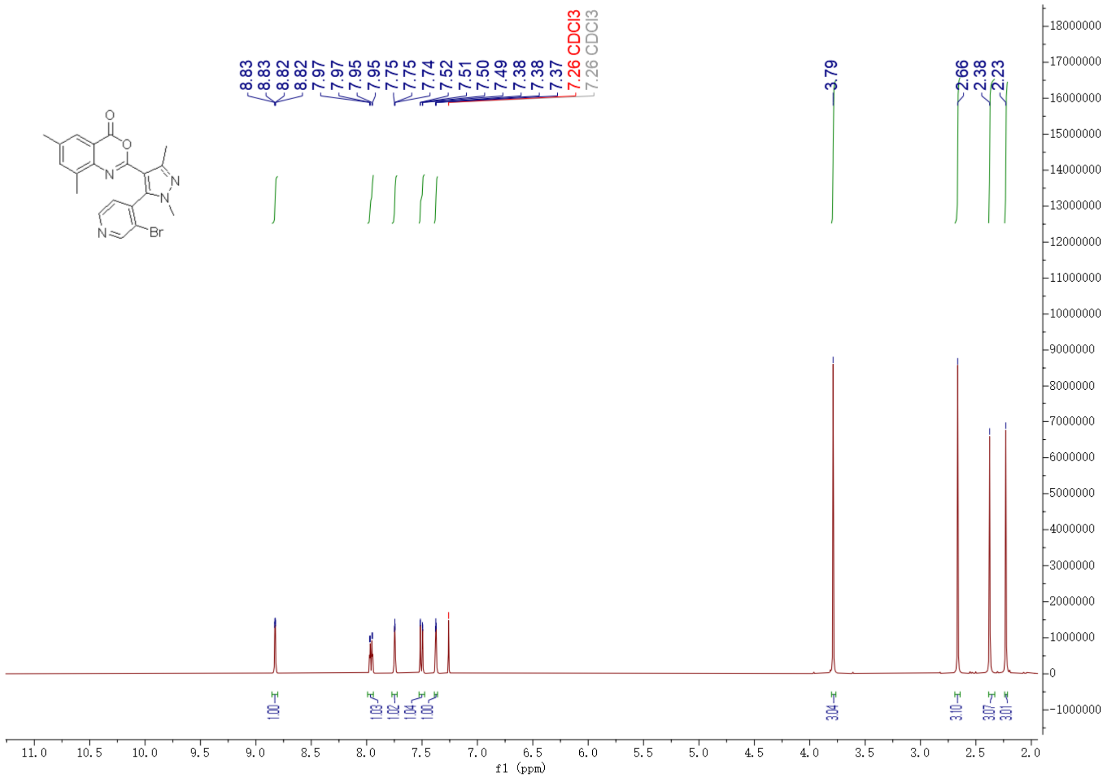

Figure S61. The ^{1}H NMR of **8n** (Chloroform-*d*).

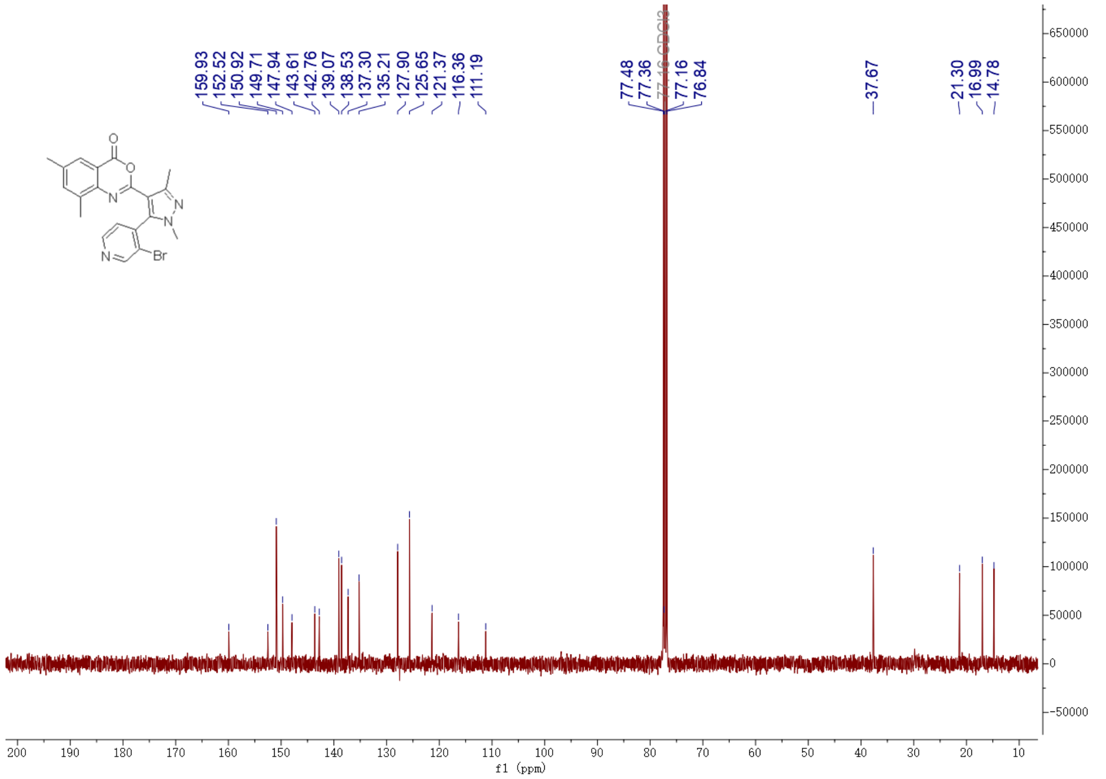

Figure S62. The ^{13}C NMR of **8n** (Chloroform-*d*)

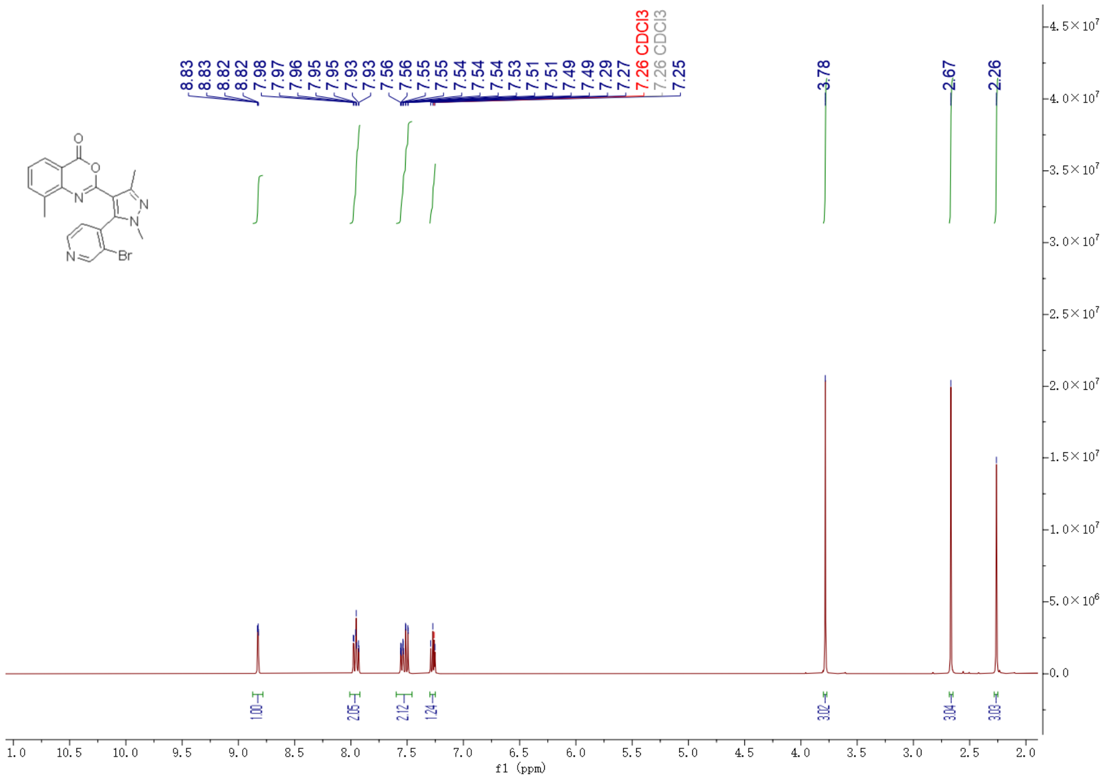

Figure S63. The ^{1}H NMR of **8o** (Chloroform-*d*).

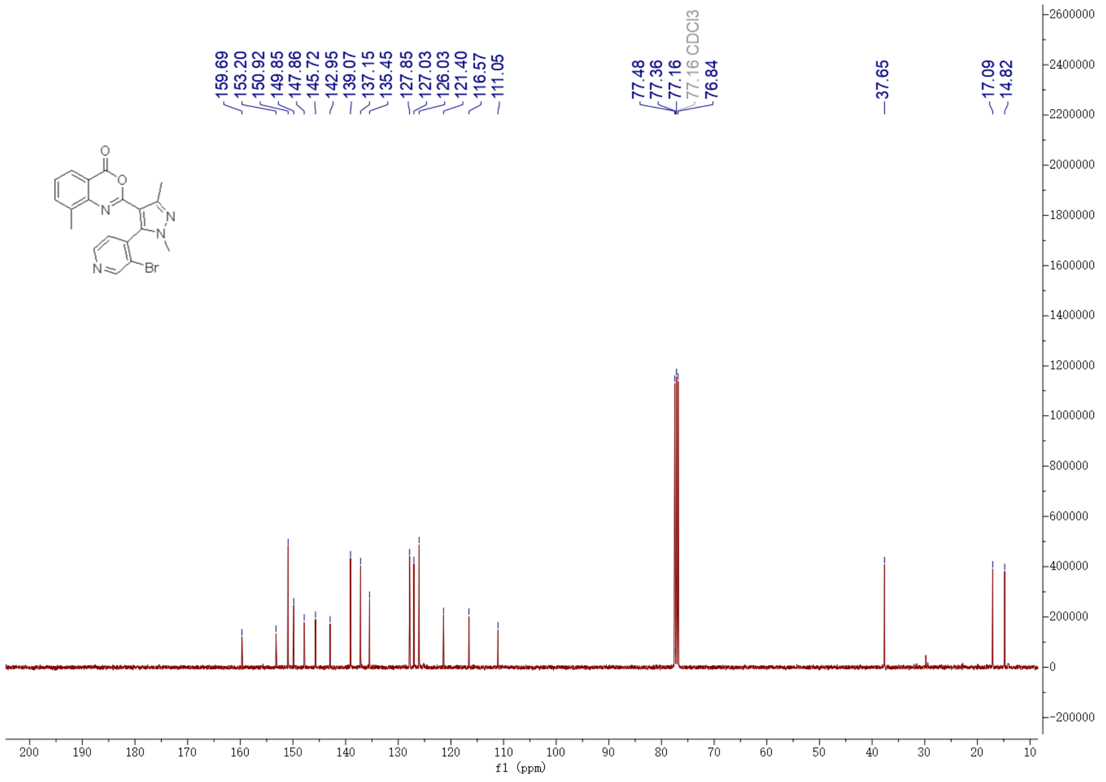

Figure S64. The ^{13}C NMR of **8o** (Chloroform-*d*)

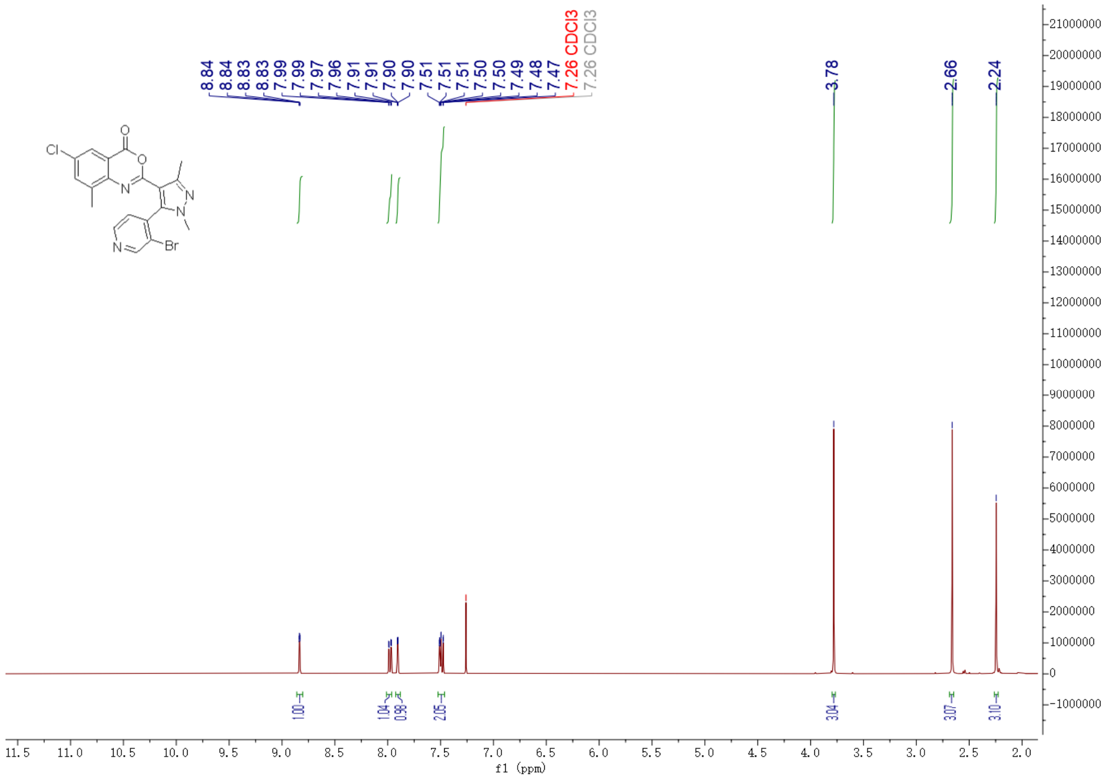

Figure S65. The ^{1}H NMR of **8p** (Chloroform-*d*).

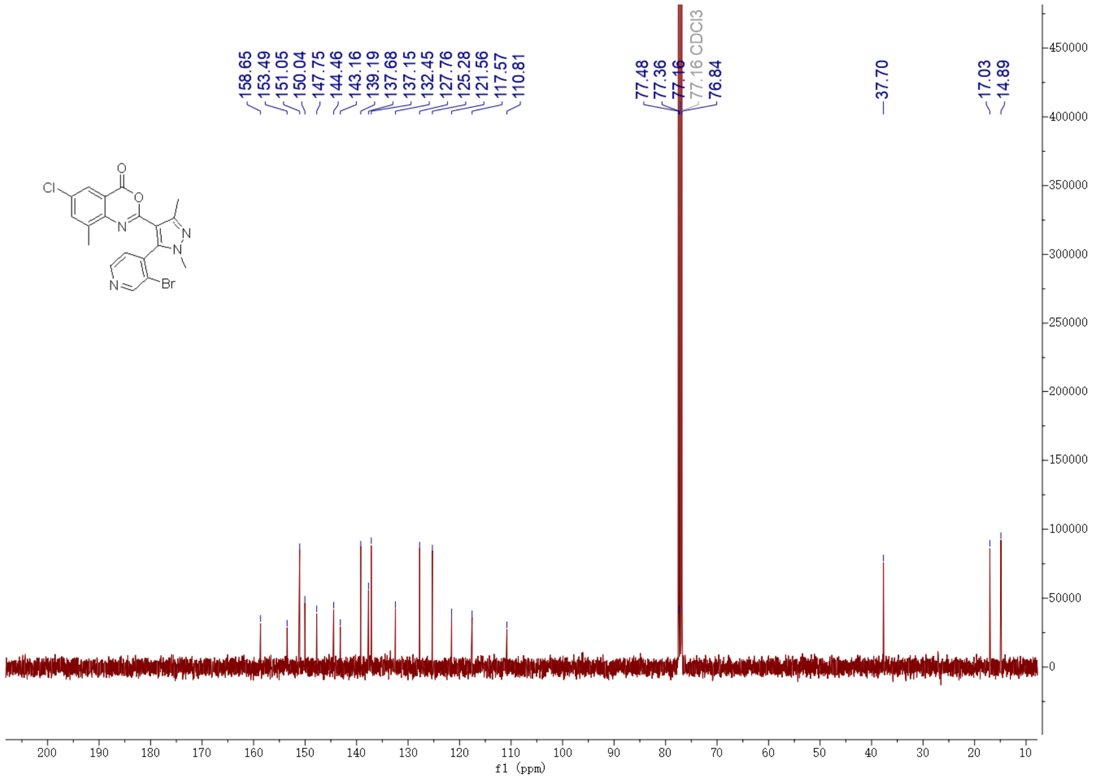

Figure S66. The ^{13}C NMR of **8p** (Chloroform-*d*)

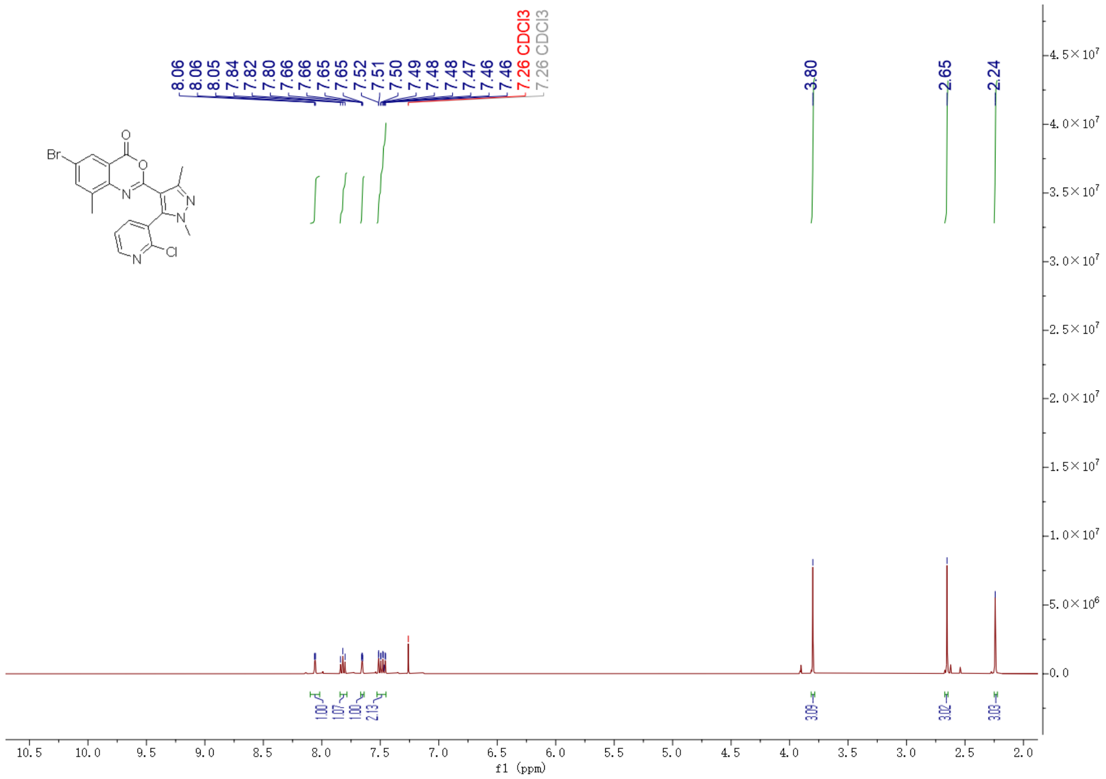

Figure S67. The ^{1}H NMR of **8q** (Chloroform-*d*).

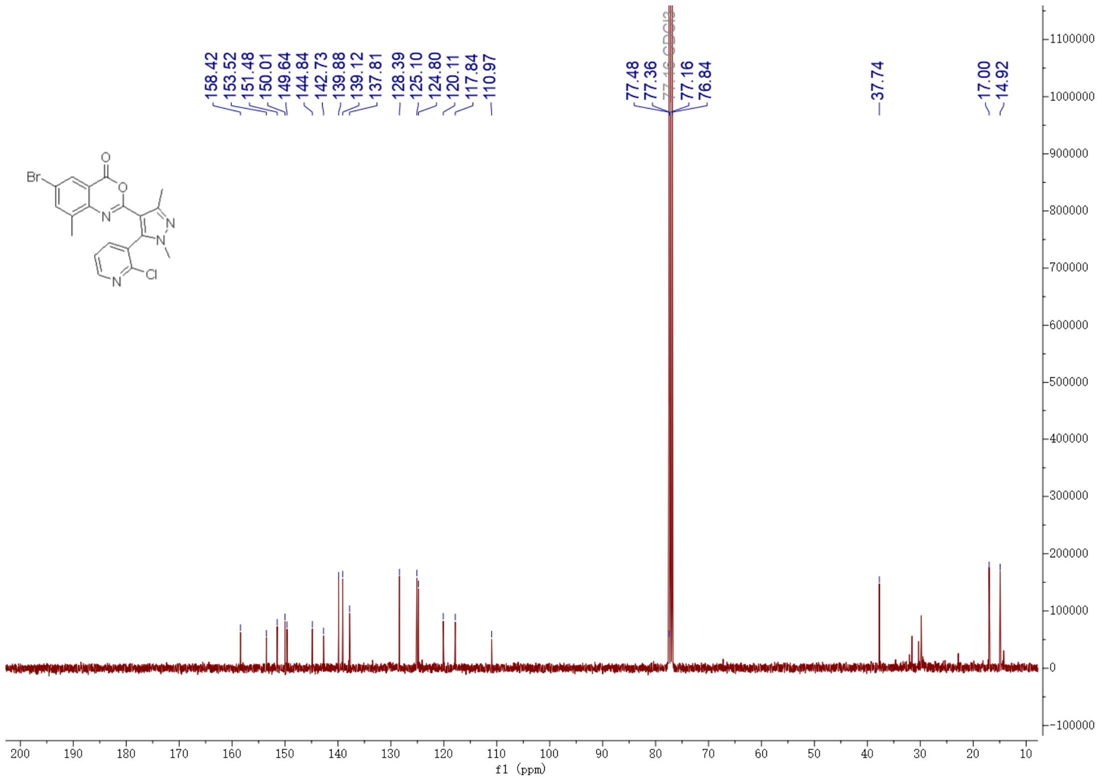

Figure S68. The ^{13}C NMR of **8q** (Chloroform-*d*)

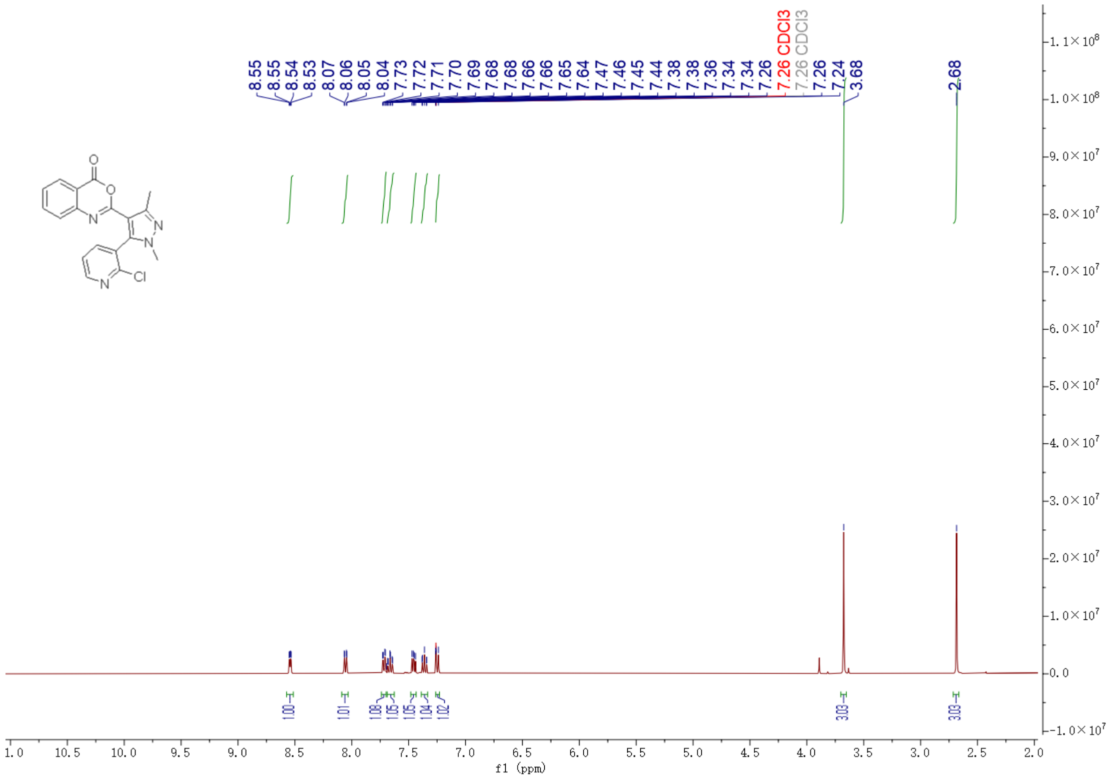

Figure S69. The ^{1}H NMR of **8r** (Chloroform-*d*).

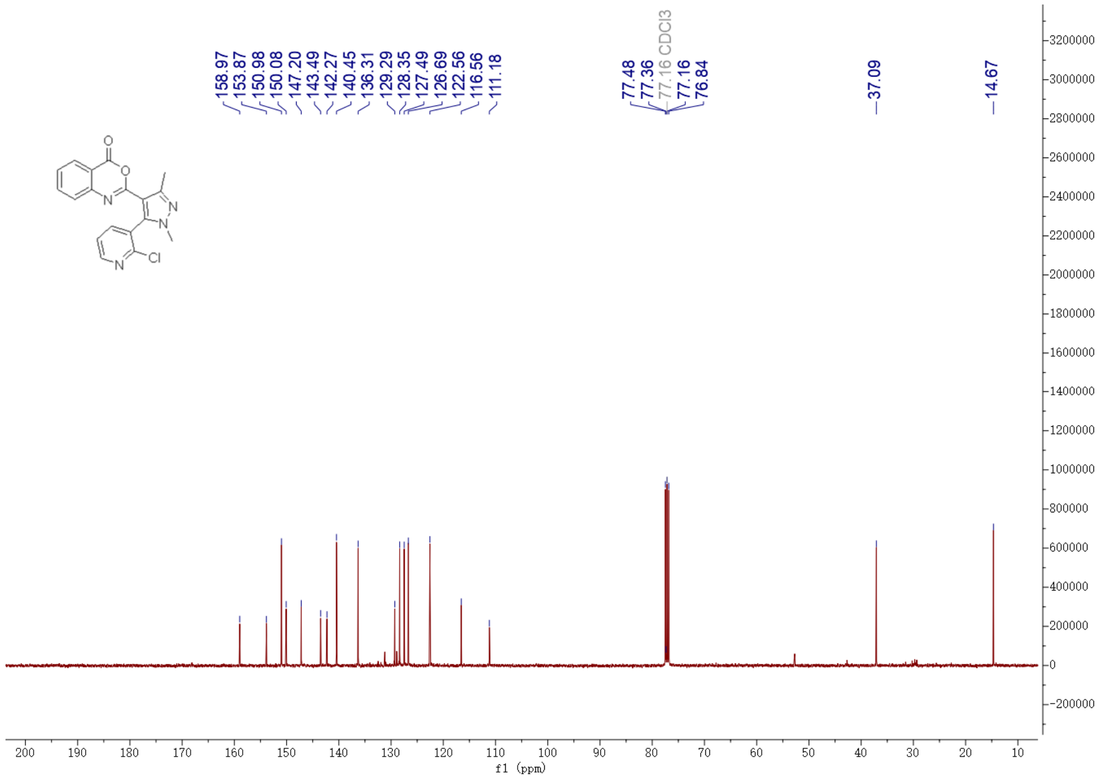

Figure S70. The ^{13}C NMR of **8r** (Chloroform-*d*)

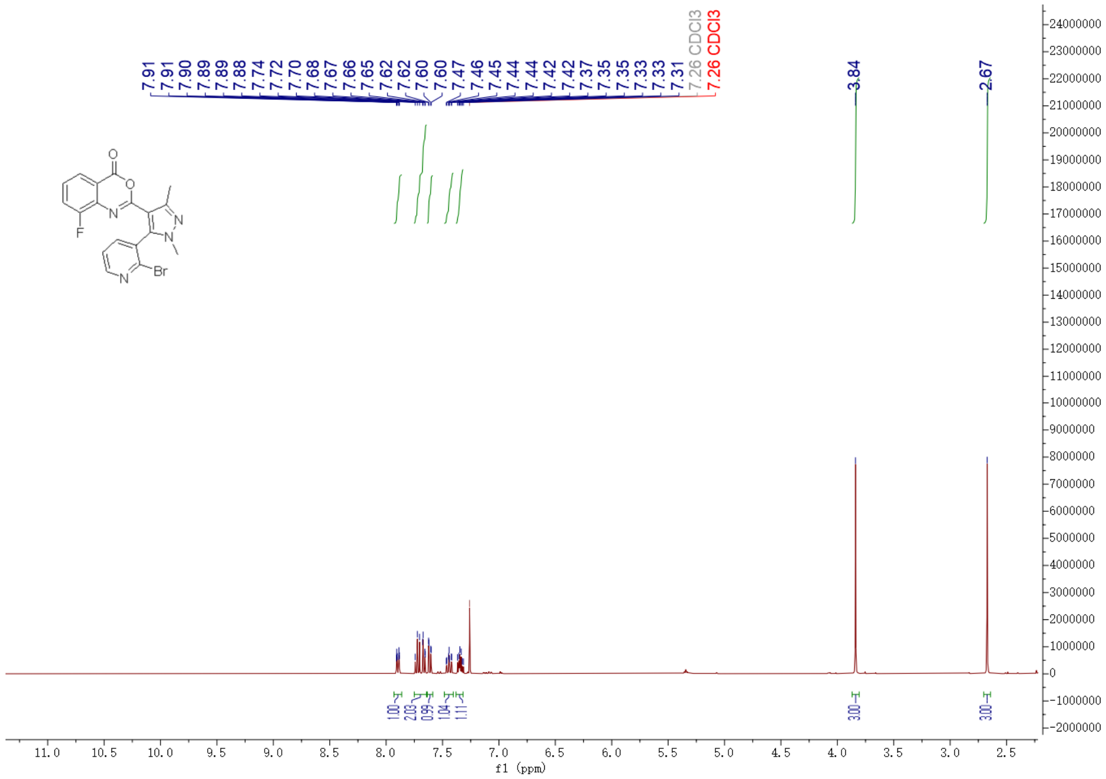

Figure S71. The ^{1}H NMR of **8s** (Chloroform-*d*).

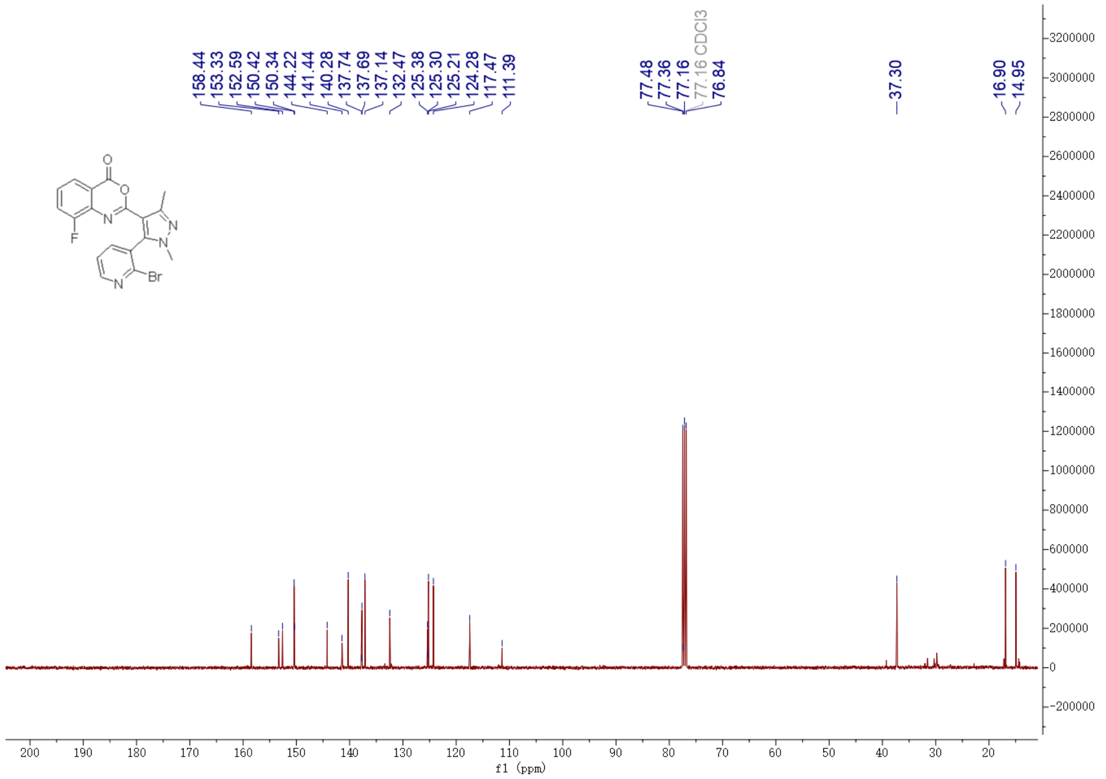

Figure S72. The ^{13}C NMR of **8s** (Chloroform-*d*)

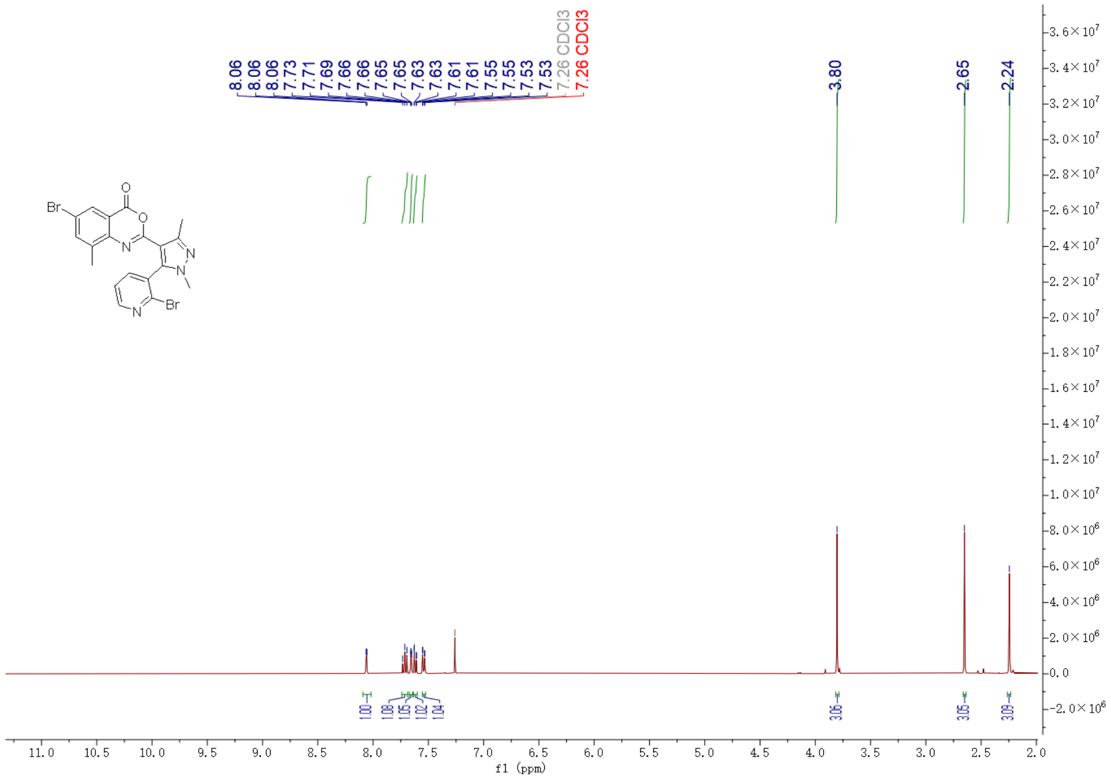

Figure S73. The ^{1}H NMR of **8t** (Chloroform-*d*).

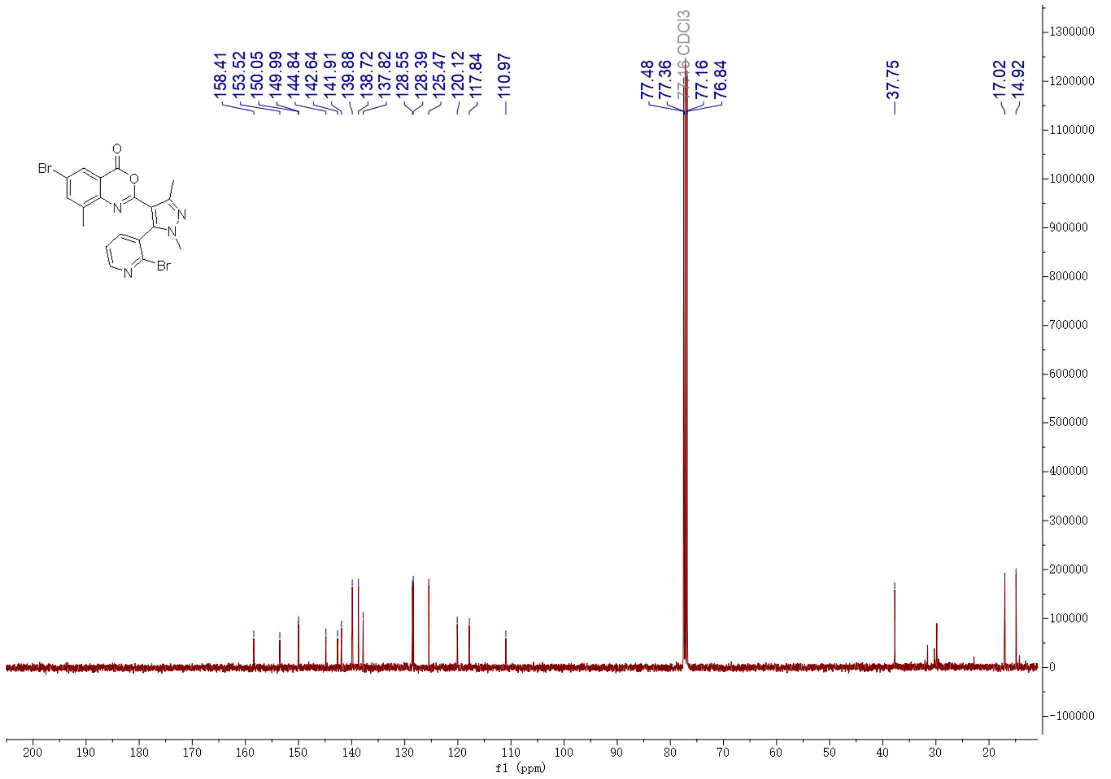

Figure S74. The ^{13}C NMR of **8t** (Chloroform-*d*)

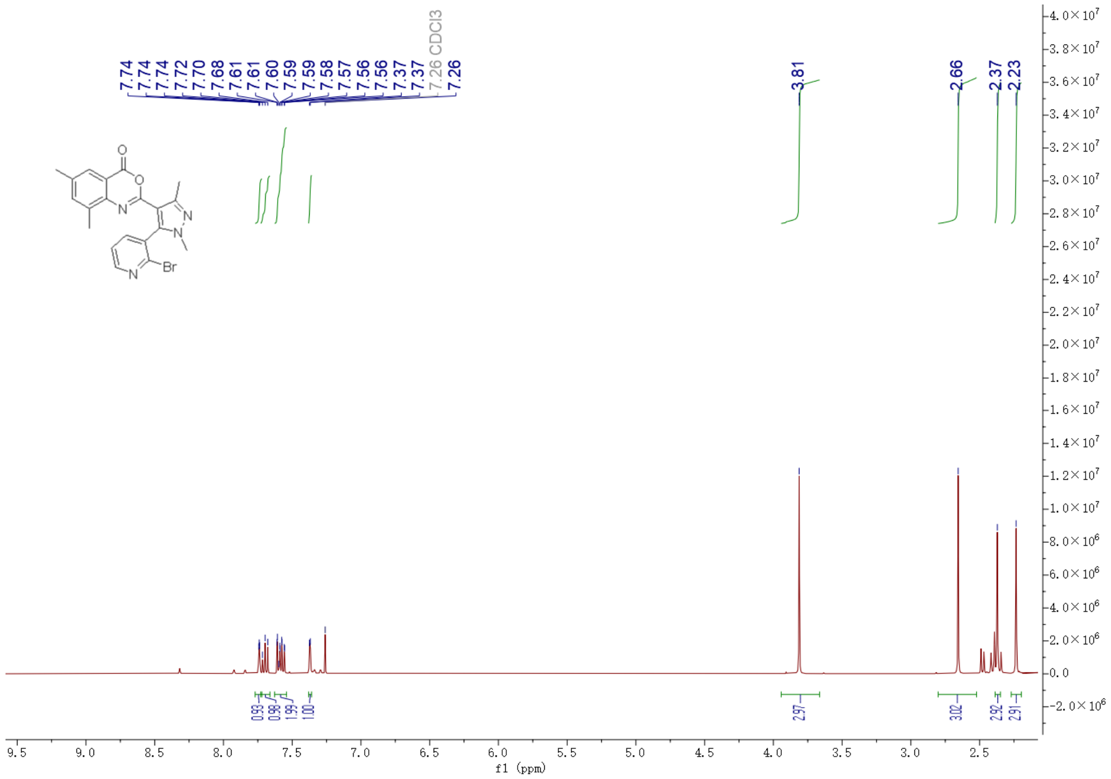

Figure S75. The ^{1}H NMR of **8u** (Chloroform-*d*).

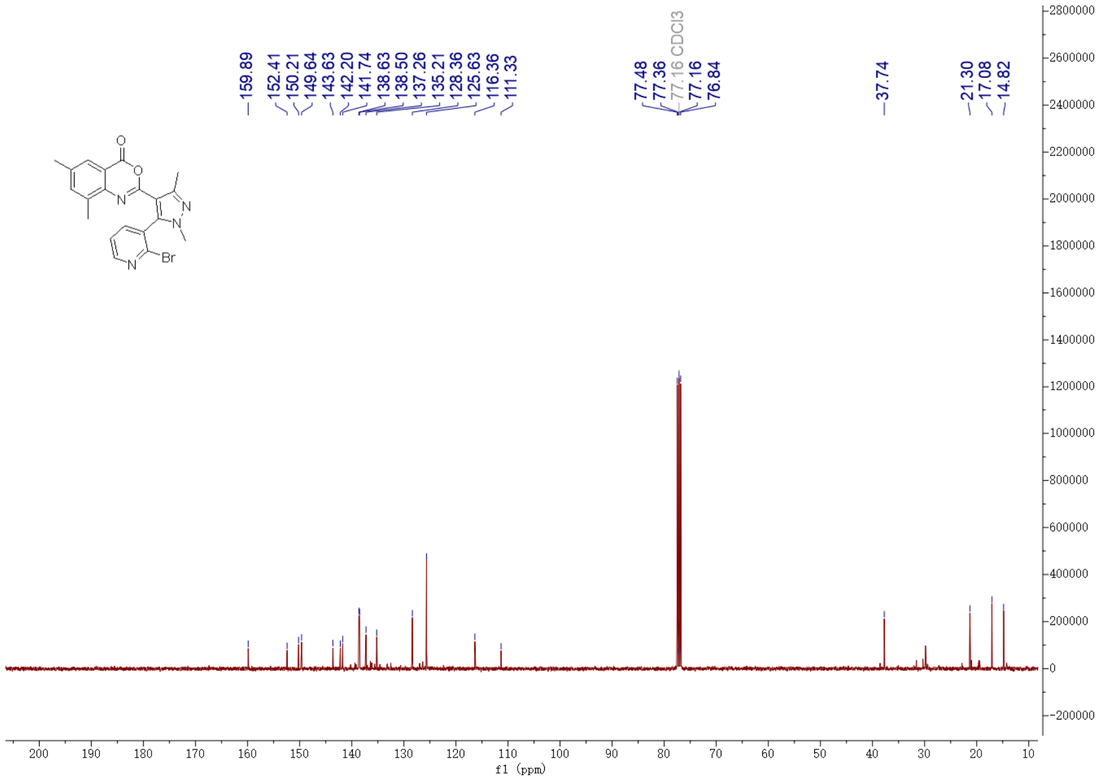

Figure S76. The ^{13}C NMR of **8u** (Chloroform-*d*)

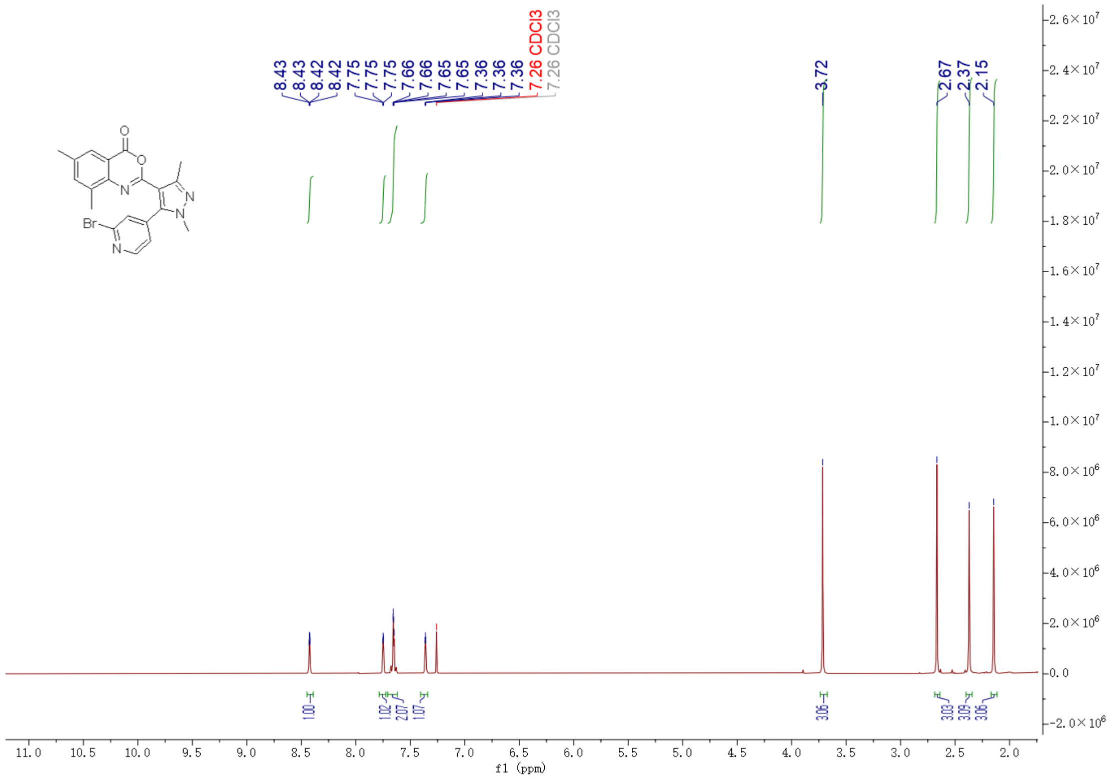

Figure S77. The ^{1}H NMR of **8v** (Chloroform-*d*).

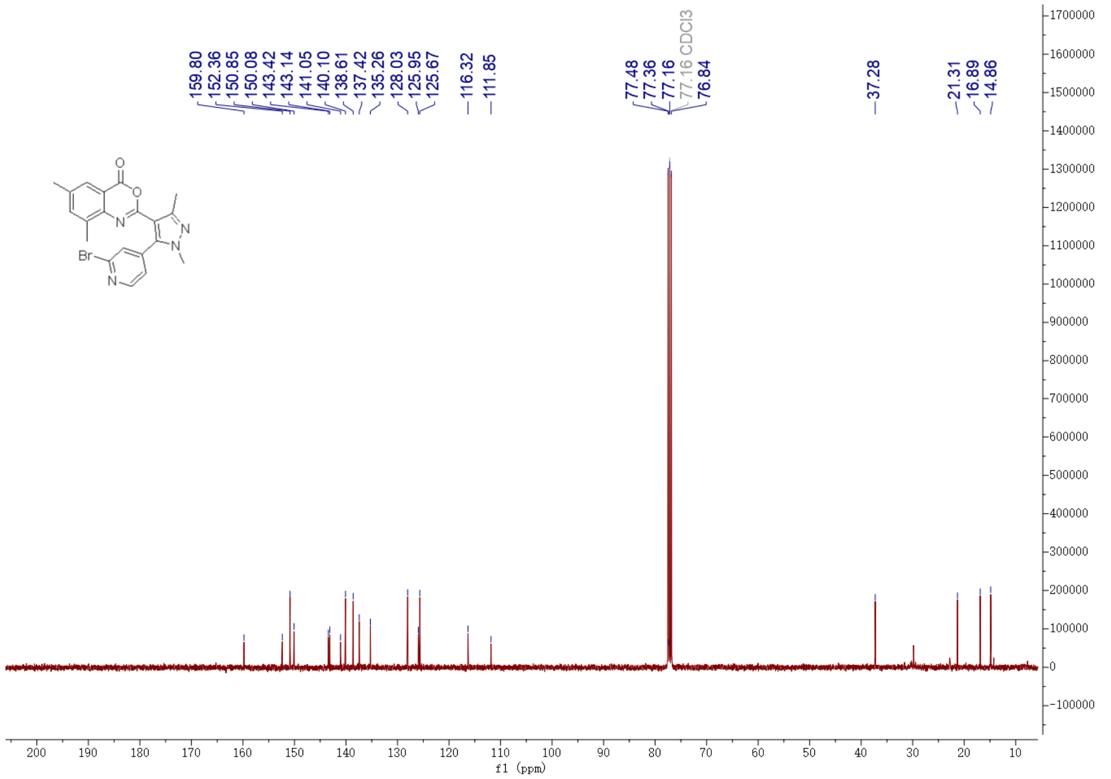

Figure S78. The ^{13}C NMR of **8v** (Chloroform-*d*)

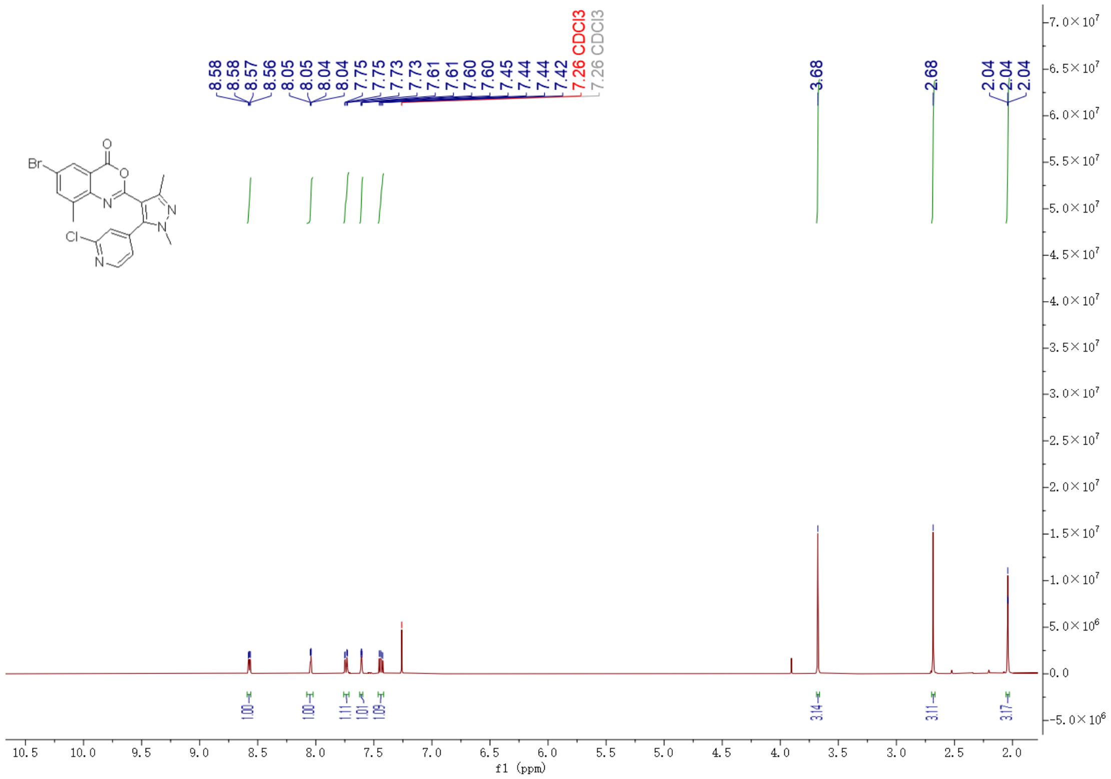

Figure S79. The ^{1}H NMR of **8w** (Chloroform-*d*).

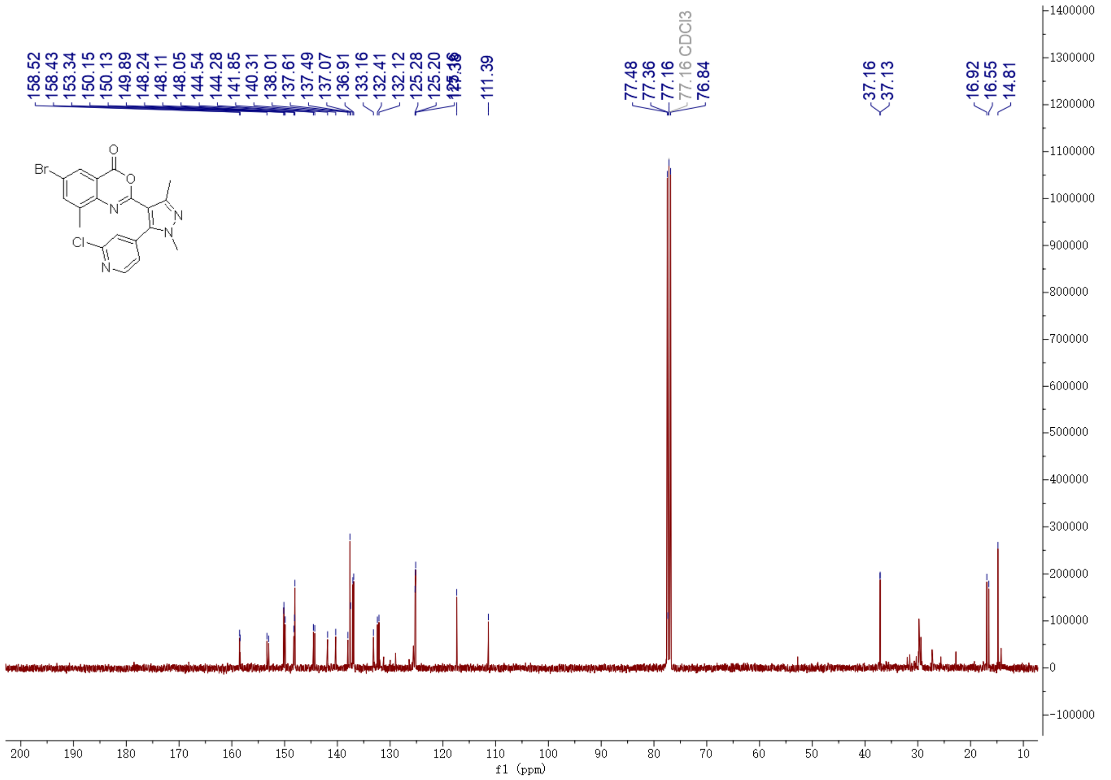

Figure S80. The ^{13}C NMR of **8w** (Chloroform-*d*)

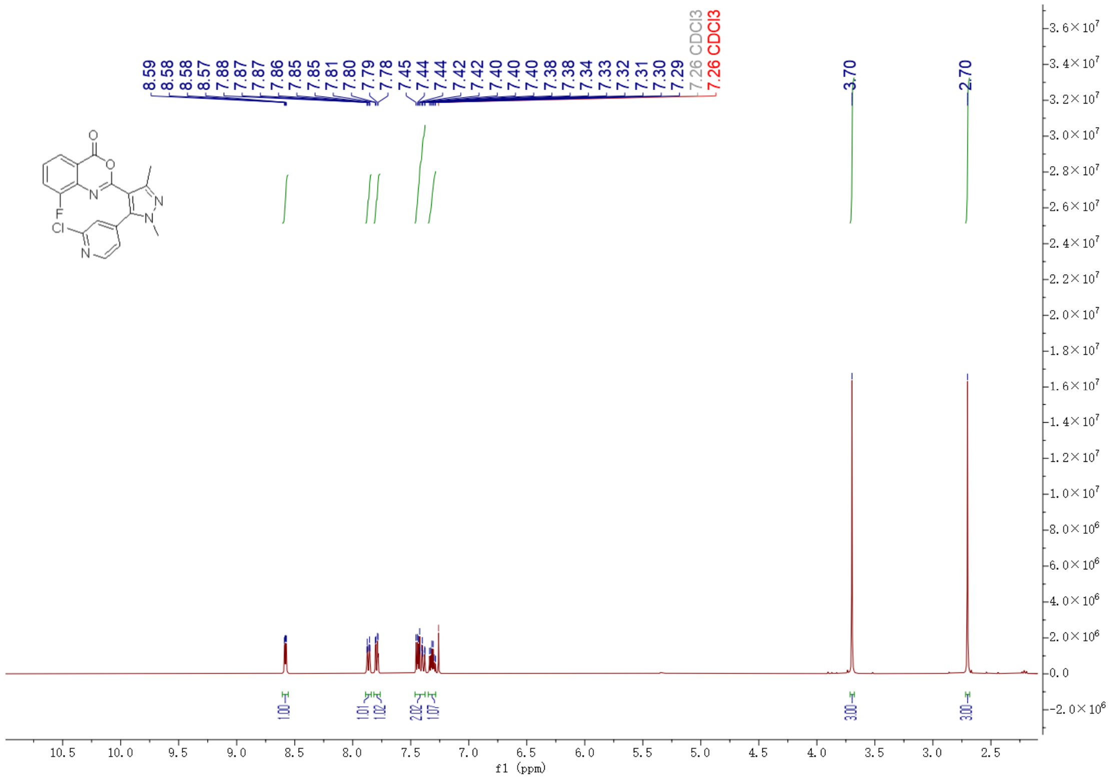

Figure S81. The ^1H NMR of **8x** (Chloroform-*d*).

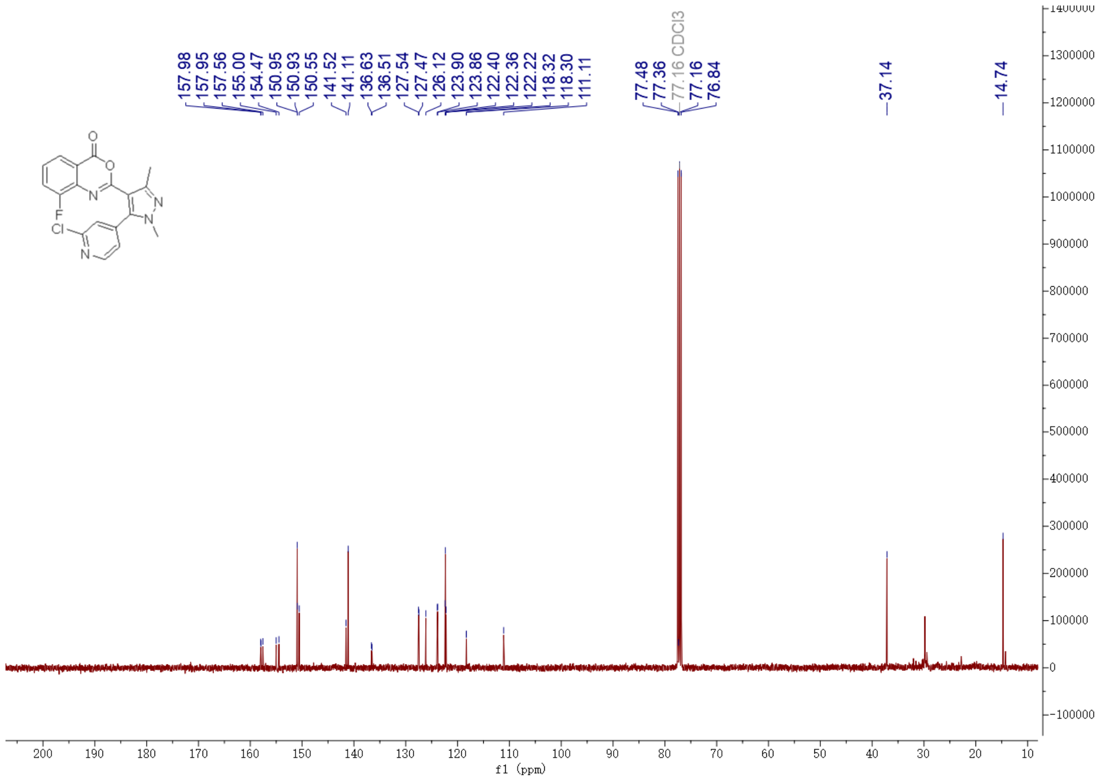

Figure S82. The ^{13}C NMR of **8x** (Chloroform-*d*)

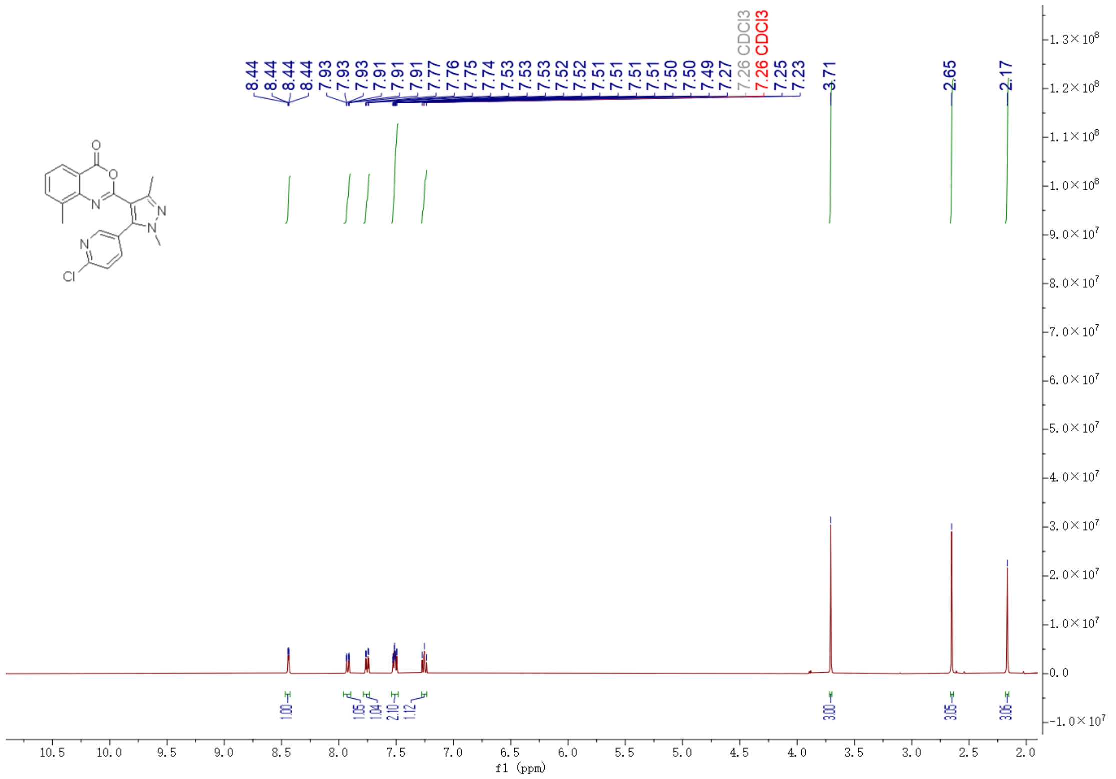

Figure S83. The ^{1}H NMR of **8y** (Chloroform-*d*).

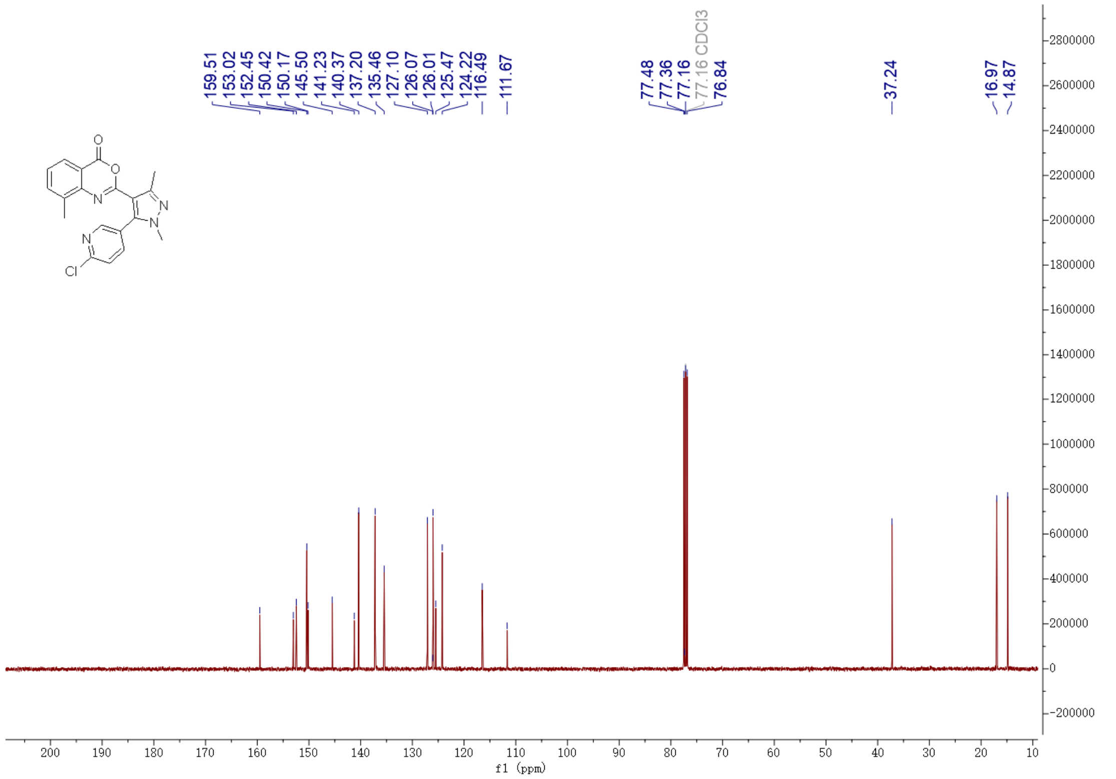

Figure S84. The ^{13}C NMR of **8y** (Chloroform-*d*)

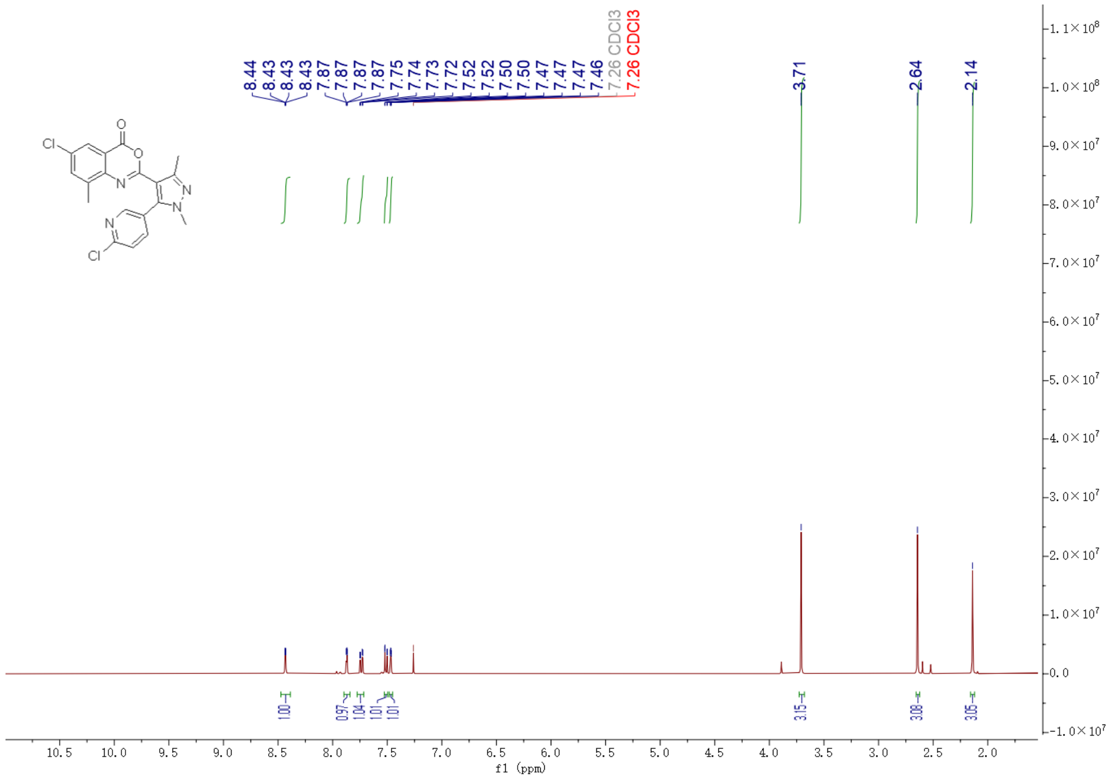

Figure S85. The ^{1}H NMR of **8z** (Chloroform-*d*).

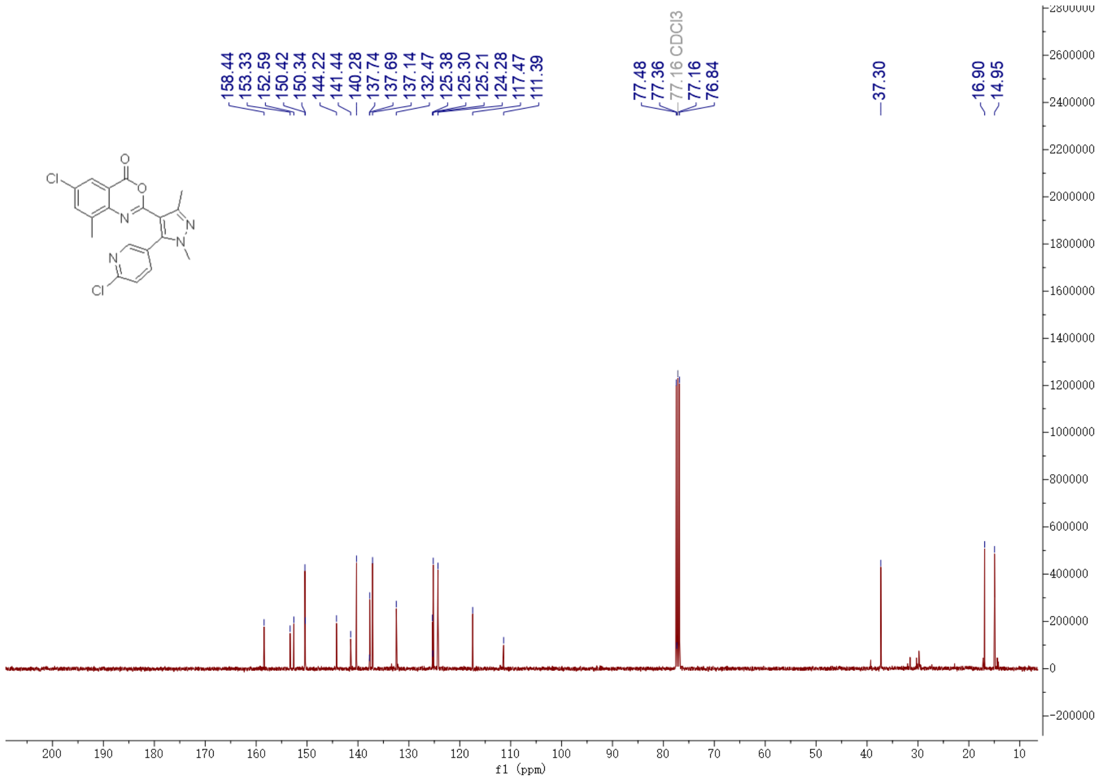

Figure S86. The ^{13}C NMR of **8z** (Chloroform-*d*).

6. Molecular docking binding energies of target compounds (8a–8z);

Table S1. Molecular docking binding energies of target compounds (**8a–8z**).

Docking Score (kcal/mol)			
**Compound**		**Compound**	
**8a**	-10.2	**8n**	-10.0
**8b**	-9.6	**8o**	-9.8
**8c**	-9.8	**8p**	-9.7
**8d**	-10.1	**8q**	-10.2
**8e**	-9.8	**8r**	-9.4
**8f**	-9.9	**8s**	-9.7
**8g**	-9.7	**8t**	-10.2
**8h**	-9.4	**8u**	-10.0
**8i**	-10.0	**8v**	-10.5
**8j**	-11.8	**8w**	-10.0
**8k**	-10.4	**8x**	-9.8
**8l**	-10.2	**8y**	-10.0
**8m**	-10.3	**8z**	-10.4

7.Inhibitory activities of potent compounds against maize rust across different concentrations;

Table S2. Inhibitory activities of potent compounds against maize rust across different concentrations

**Compound**	**Fungicidal activity(%)**					
	**200mg/L**	**100mg/L**	**50mg/L**	**25mg/L**	**10mg/L**	**2mg/L**
**8a**	87.0	/	84.4	51.0	35.0	/
**8j**	100	67.0	62.0	65.0	/	/
**8l**	90.0	/	82.0	45.0	31.0	18.5
**8p**	97.0	/	92.0	54.3	36.7	21.5

8.Synthetic Steps and Respective ^{1}H NMR of Diazonium Salts;

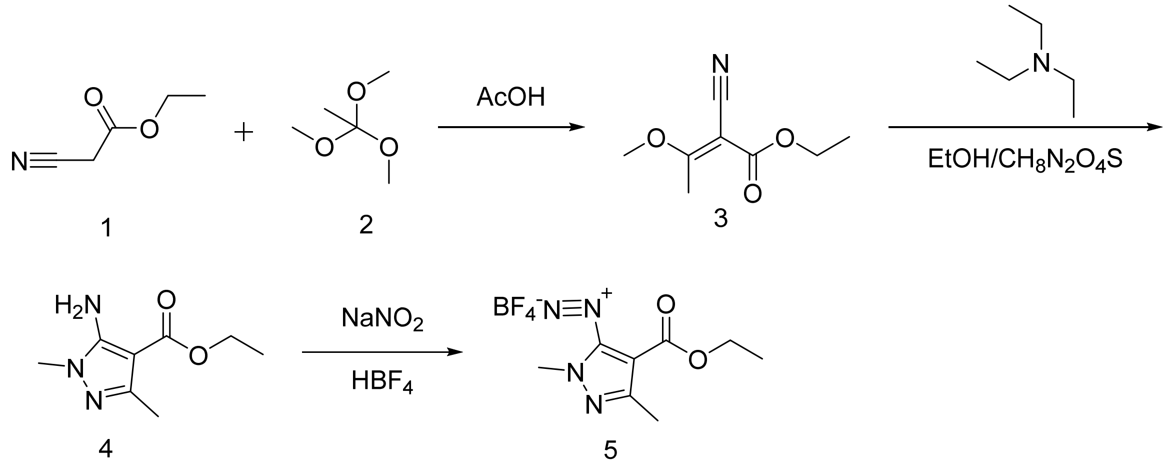

Figure S87. Synthetic Procedures of Diazonium Salts.

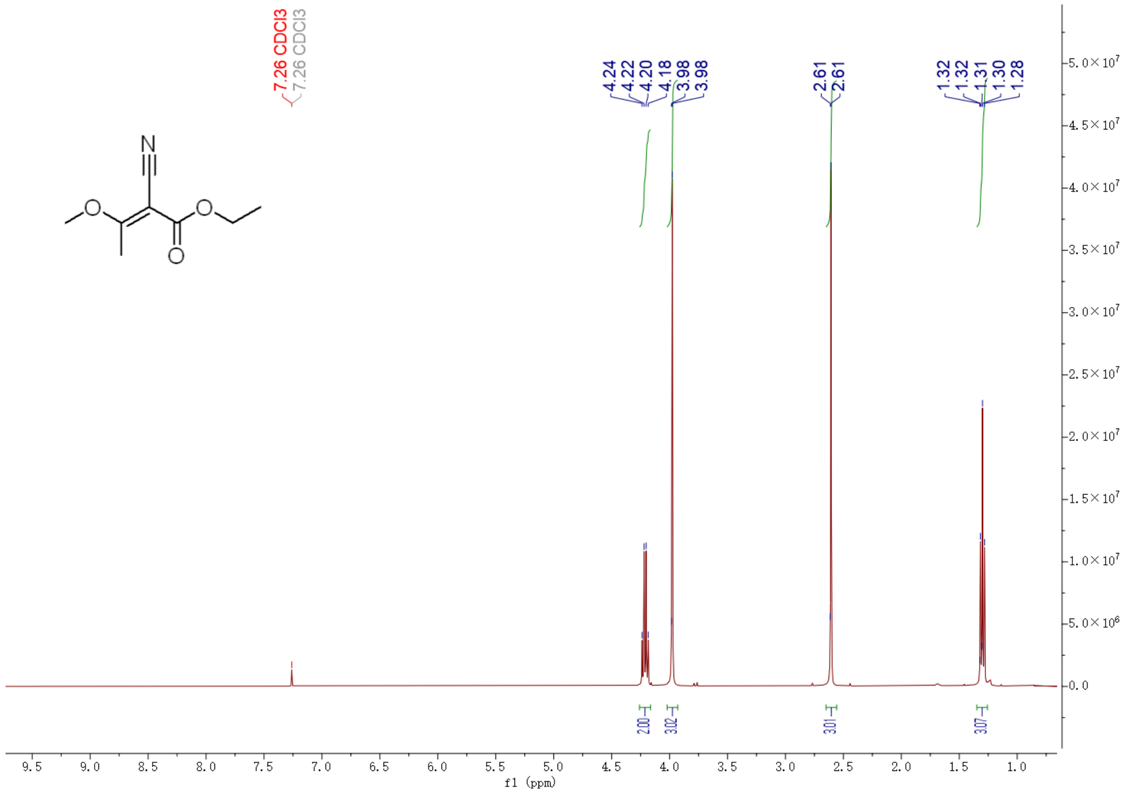

Figure S88. ^{1}H NMR Spectra of Diazonium Intermediate **3** (Chloroform-*d*).

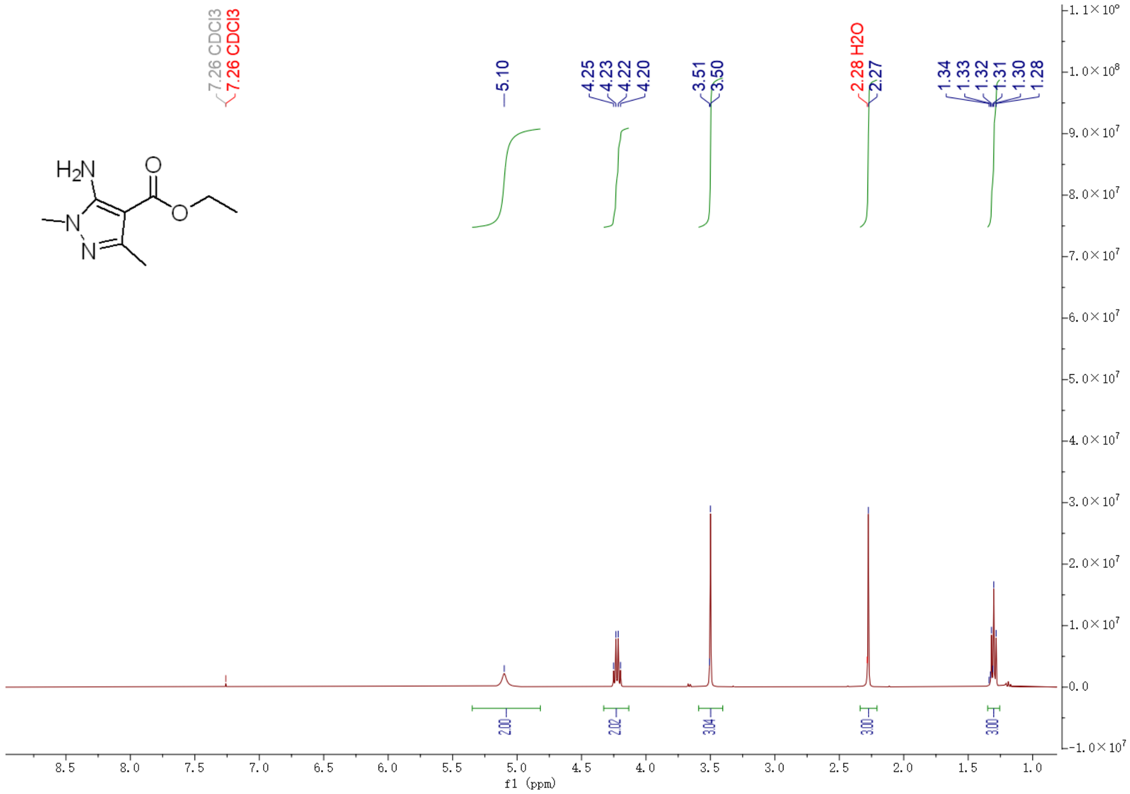

Figure S89. ^{1}H NMR Spectra of Diazonium Intermediate **4** (Chloroform-*d*).

9.HPLC-MS of Compounds with Potential Biological Activity.

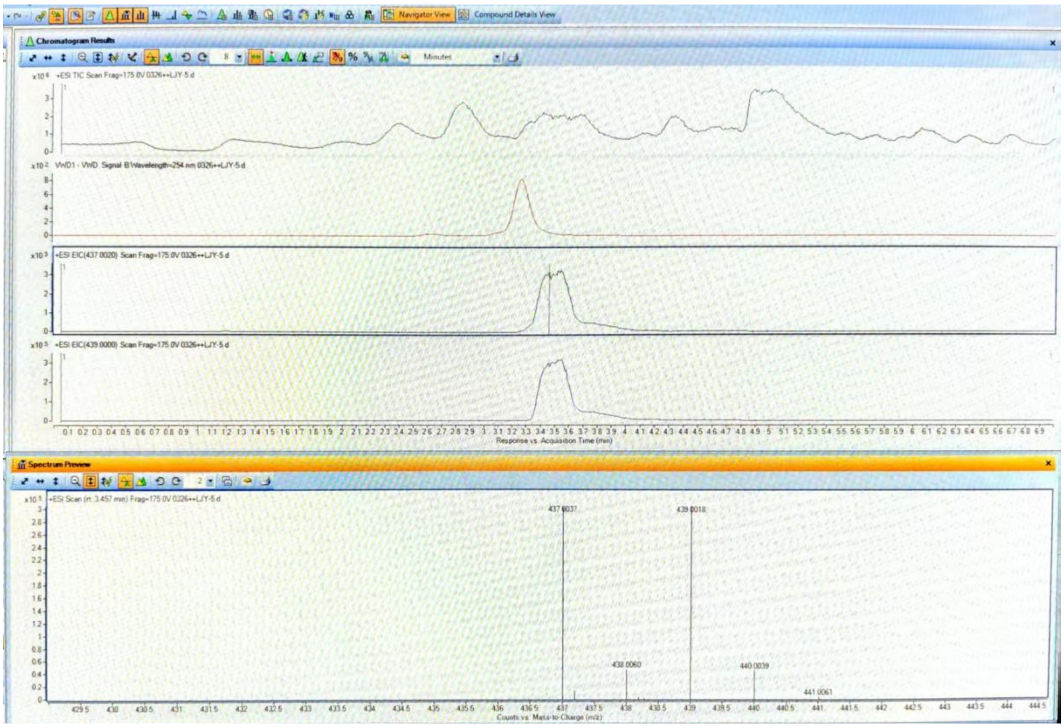

Figure S90. The HPLC-MS of Compound **8b.**

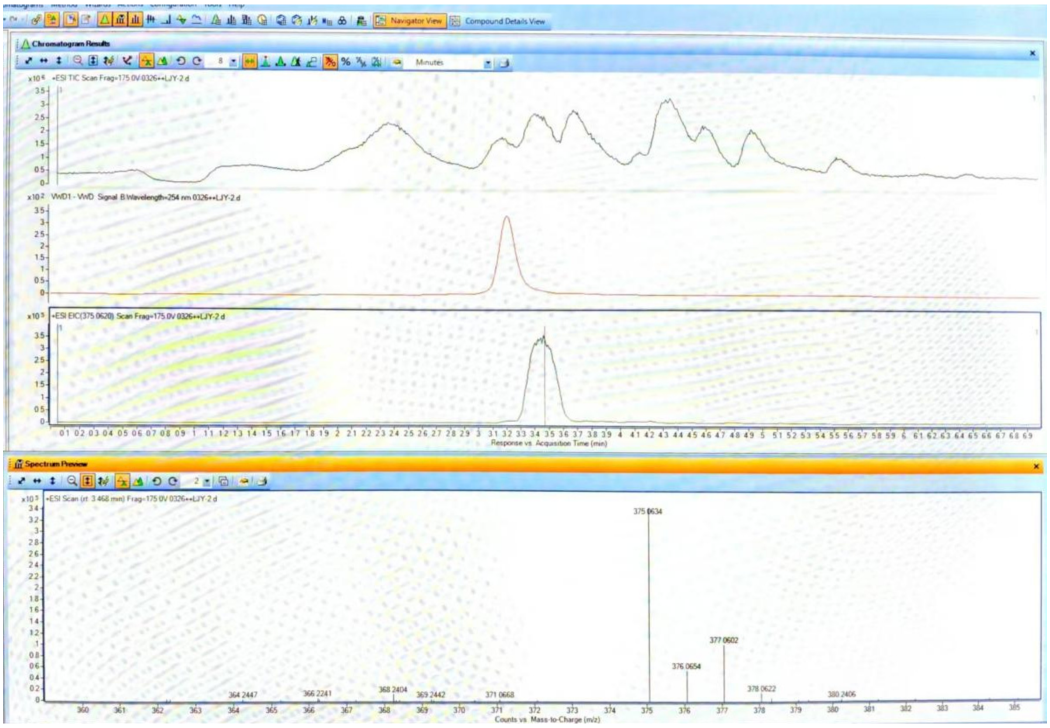

Figure S91. The HPLC-MS of Compound **8h.**

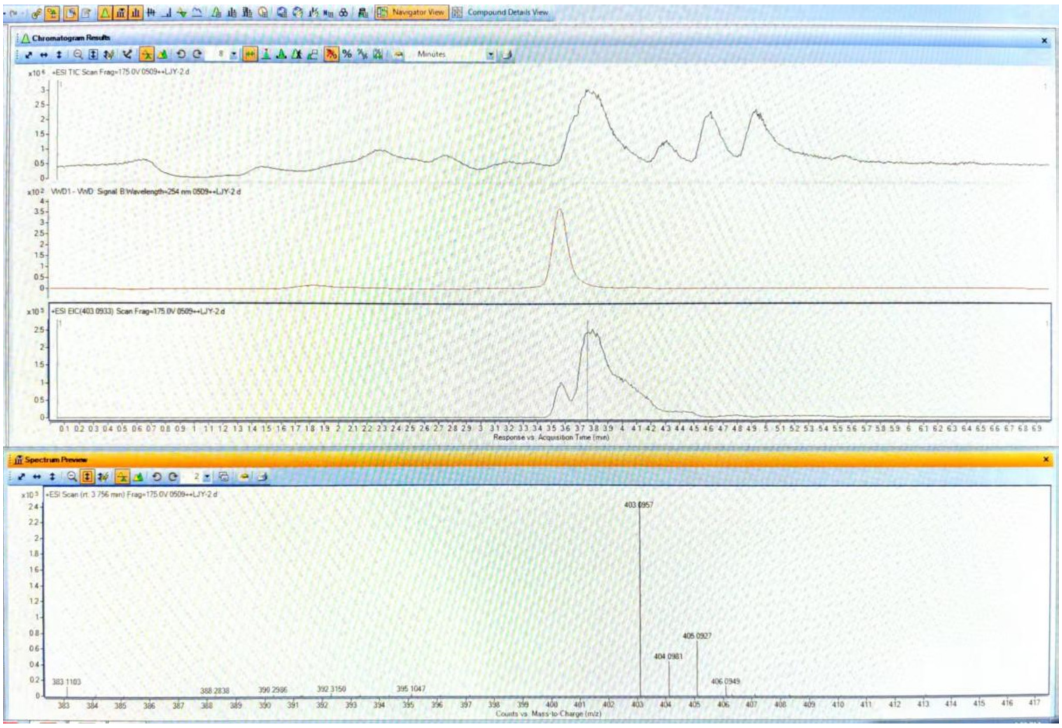

Figure S92. The HPLC-MS of Compound **8j.**

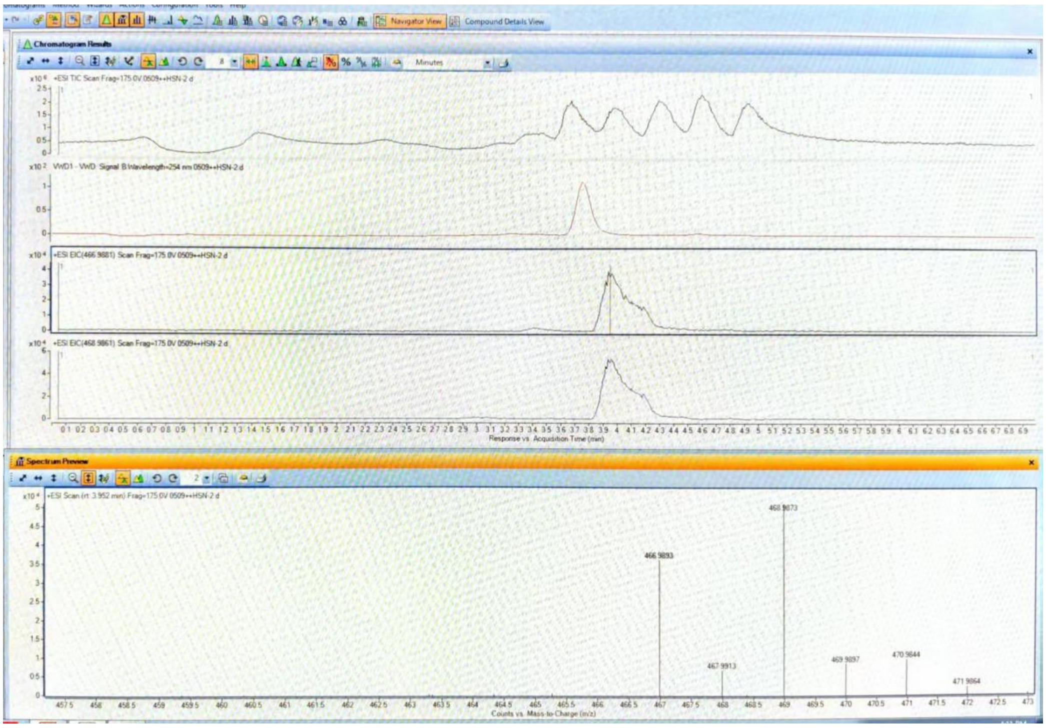

Figure S93. The HPLC-MS of Compound **8p.**

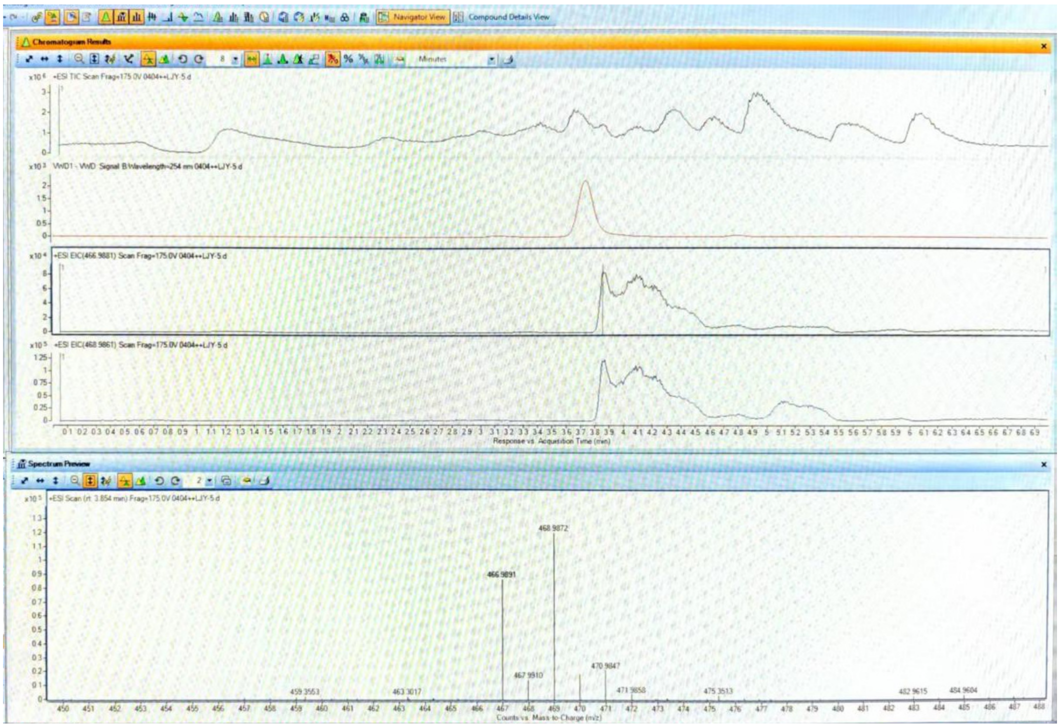

Figure S94. The HPLC-MS of Compound **8q.**

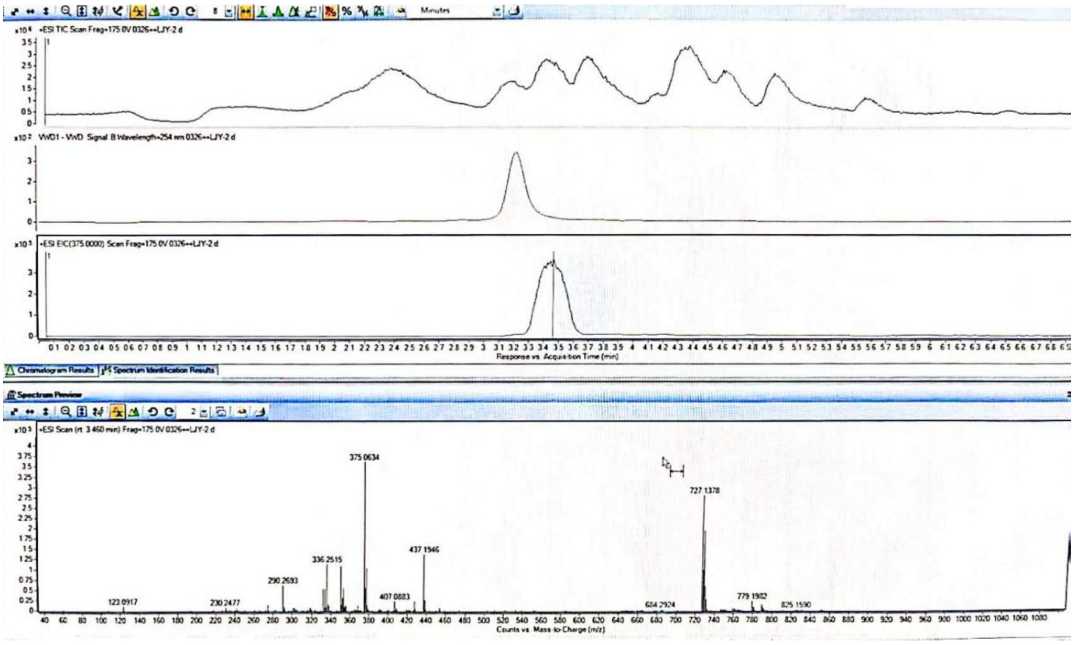

Figure S95. The HPLC-MS of Compound **8r.**

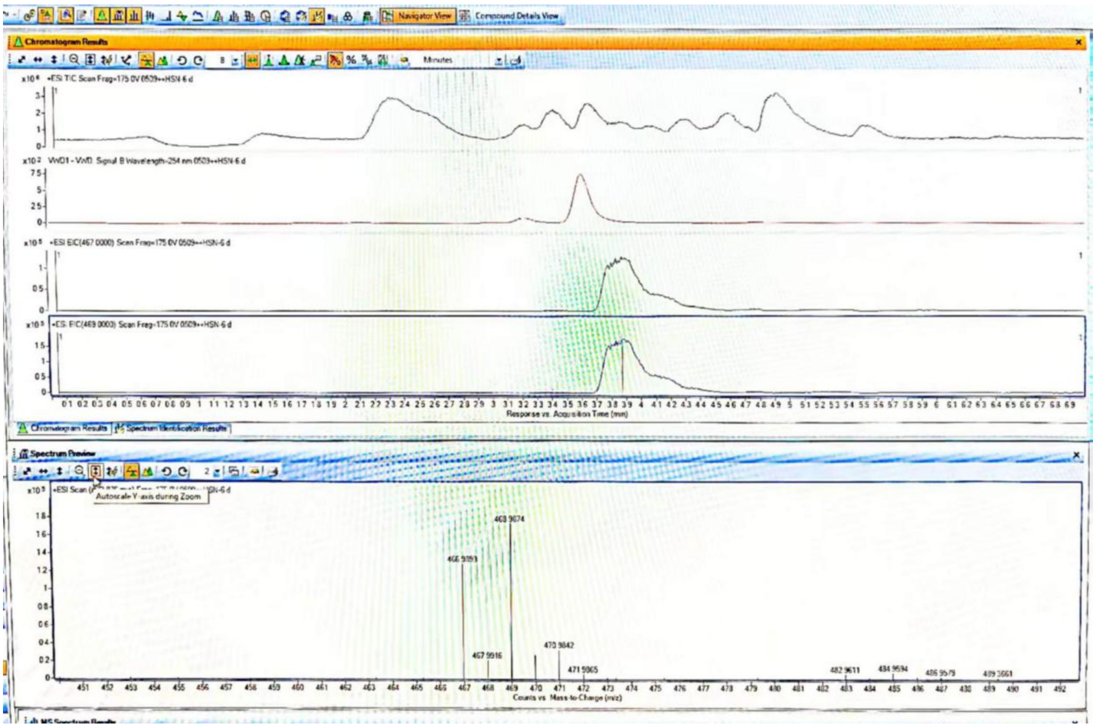

Figure S96. The HPLC-MS of Compound **8w.**
